# Multiple Small-Effect Alleles of *Indica* Origin Enhance High Iron-Associated Stress Tolerance in Rice Under Field Conditions in West Africa

**DOI:** 10.3389/fpls.2020.604938

**Published:** 2021-01-15

**Authors:** Giovanni Melandri, Mouritala Sikirou, Juan D. Arbelaez, Afeez Shittu, Vimal K. Semwal, Kadougoudiou A. Konaté, Alhassan T. Maji, Steven A. Ngaujah, Inoussa Akintayo, Vishnu Govindaraj, Yuxin Shi, Francisco J. Agosto-Peréz, Anthony J. Greenberg, Gary Atlin, Venuprasad Ramaiah, Susan R. McCouch

**Affiliations:** ^1^Plant Breeding and Genetics, Cornell University, Ithaca, NY, United States; ^2^Africa Rice Center, Ibadan, Nigeria; ^3^School of Horticulture and Green Landscaping, Kétou, Bénin; ^4^Environmental Institute for Agricultural Research, Ouagadougou, Burkina Faso; ^5^National Root Crops Research Institute, Umuahia, Nigeria; ^6^Sierra Leone Agricultural Research Institute, Rokupr, Sierra Leone; ^7^Central Agricultural Research Institute, Suakoko, Liberia; ^8^Africa Rice Center, Suakoko, Liberia; ^9^Bayesic Research, Ithaca, NY, United States; ^10^Bill & Melinda Gates Foundation, Seattle, WA, United States

**Keywords:** iron toxicity, high iron associated (HIA) stress, QTL mapping, genome wide association studies (GWAS), haplotype analysis, *Oryza sativa*, NERICA

## Abstract

Understanding the genetics of field-based tolerance to high iron-associated (HIA) stress in rice can accelerate the development of new varieties with enhanced yield performance in West African lowland ecosystems. To date, few field-based studies have been undertaken to rigorously evaluate rice yield performance under HIA stress conditions. In this study, two NERICA × *O. sativa* bi-parental rice populations and one *O.sativa* diversity panel consisting of 296 rice accessions were evaluated for grain yield and leaf bronzing symptoms over multiple years in four West African HIA stress and control sites. Mapping of these traits identified a large number of QTLs and single nucleotide polymorphisms (SNPs) associated with stress tolerance in the field. Favorable alleles associated with tolerance to high levels of iron in anaerobic rice soils were rare and almost exclusively derived from the *indica* subpopulation, including the most favorable alleles identified in NERICA varieties. These findings highlight the complex genetic architecture underlying rice response to HIA stress and suggest that a recurrent selection program focusing on an expanded *indica* genepool could be productively used in combination with genomic selection to increase the efficiency of selection in breeding programs designed to enhance tolerance to this prevalent abiotic stress in West Africa.

## Introduction

High iron-associated (HIA) stress, often referred to as iron (Fe)-toxicity in the literature, is a nutritional disorder that affects rice production in many cultivated areas of the world, including Africa, Asia and South America (Fageria et al., 2008). Critical to its occurrence are soil conditions of low pH (typical of acid soils), low cation exchange capacity (low clay content) and the presence of high amounts of Fe. HIA stress affects only lowland rice production (irrigated or rainfed) when prolonged waterlogging results in anaerobic conditions that promote the microbial reduction of insoluble ferric (Fe^3+^) into soluble ferrous (Fe^2+^) iron. When Fe^2+^ is absorbed by the roots, it readily reaches toxic levels (Becker and Asch, 2005; Sahrawat, 2005). In West Africa, HIA stress is a widespread nutritional disorder and it was recently estimated that rainfed lowland rice grown on Fe-rich soils represents approximately 20% of the total rice area of the region (van Oort, 2018). The typical visual symptom associated with HIA stress in rice is the appearance of necrotic brown spots on the leaves (bronzing), accompanied by broad variation in yield loss, ranging from 10% to 100%, depending on the sensitivity of the rice variety and the intensity of the stress (Audebert and Fofana, 2009).

Enhancing the abiotic stress tolerance of high-quality and high-yielding *O. sativa* varieties widely grown by farmers and accepted by consumers is a fundamental target of rice breeding programs (Mackill and Khush, 2018). Since the 1970s, HIA stress tolerant *O. sativa* varieties (mostly from Asia) have been evaluated by West African breeders and introduced for use as parents in the development of new tolerant lines that, in some cases (i.e., WITA 4), have been widely and successfully adopted by farmers (Sikirou et al., 2015). Many of the *O. sativa* lines exhibited site-specific tolerance to West African conditions, dependent on regional soil types and environmental conditions (Becker and Asch, 2005). As a consequence, the goal of developing new high-yielding varieties widely adapted to HIA stress environments in West Africa is still far from being achieved.

Another strategy to improve HIA stress tolerance in rice is to exploit the genetic diversity present in African rice, *O. glaberrima.* In general, *O. glaberrima* is lower-yielding than *O. sativa*, but it tends to be better adapted to the soil conditions and climate typical of African environments (Linares, 2002). A major obstacle in using *O. glaberrima* donors to improve HIA stress tolerance of elite *O. sativa* varieties is the hybrid sterility that occurs when these two species are crossed (Ghesquière et al., 1997). At the Africa Rice Center (AfricaRice), Jones et al. (1997) overcame this problem and obtained fertile interspecific progenies between the two species through backcrossing and double haploid breeding. The new varieties generated by this approach were named NERICAs (NEw RICe for Africa) and were initially developed for upland rice cultivation (Jones et al., 1997). Later, new NERICA varieties for rainfed lowland cultivation (NERICA-L) were developed from crosses between *O. sativa* and *O. glaberrima* (Heuer et al., 2004), followed by three or four generations of backcrosses to the *O. sativa* recurrent parents. Among the progeny of these crosses, NERICA-L-19 (TOG5681/3^∗^IR64) displayed good yield and HIA stress tolerance (Dramé et al., 2011) and was released and adopted in many West African countries (Sié, 2008). NERICA-L-43 (TOG5674/4^∗^IR 31785) was another promising Fe-tolerant derivative from this program (Sié, 2008; Ndjiondjop et al., 2018). Based on these successes, the entire *O. glaberrima* collection present in the AfricaRice genebank (more than 2,000 accessions) underwent a large multi-environment and multi-year screening for HIA stress tolerance in West Africa and promising lines were identified (Sikirou et al., 2018). These lines represent valuable donors to be used as parents in breeding for HIA stress-tolerant varieties, but nothing is known about the genetic basis of their tolerance. However, it is known that the NERICAs contain a relatively small number of *O. glaberrima* introgressions in a largely *O. sativa* genetic background (Ndjiondjop et al., 2018; Yamamoto et al., 2018) and in some cases, these introgressions are associated with tolerance to abiotic and biotic stresses. Identifying specific genes or QTLs within these introgressions would facilitate the transfer of *O. glaberrima-*derived tolerance to high-quality *O. sativa* varieties that possess the grain quality preferred by African consumers.

In this study, we used bi-parental and association mapping, together with sequencing, to identify QTLs (Quantitative Trait Locus) and SNP (Single Nucleotide Polymorphism) markers associated with useful levels of field-based HIA stress tolerance for use in the AfricaRice breeding program. The objective was to identify favorable QTL alleles that could be reliably targeted by marker-assisted selection (MAS) to improve the performance of popular *O. sativa* varieties under HIA stress conditions in West Africa. To address this objective, we analyzed two bi-parental mapping populations for QTLs, generated re-sequencing data for a collection of 32 breeding lines from the AfricaRice program, and undertook a genome-wide association study (GWAS) using an *O. sativa* diversity panel. The mapping populations were derived from crosses between two Fe-tolerant NERICA varieties, NERICA-L-19 and NERICA-L-43, and the popular, but Fe-susceptible, Asian variety IR64-Sub1. Both populations were grown in the field in two West African HIA stress hotspots and used to map large-effect QTLs associated with yield and HIA stress tolerance. The AfricaRice breeding lines consisted of a group of 11 *O. sativa* (including IR64-Sub1, NERICA-L-19, WITA 4) and 21 *O. glaberrima* accessions including TOG 5681, CG14 and the best lines selected by Sikirou et al. (2018) used for improving tolerance to multiple abiotic stresses. The Rice Diversity Panel (Zhao et al., 2011) consisted of a collection of 296 diverse *O. sativa* varieties that was evaluated in different West African HIA stress sites over two years for grain yield and leaf bronzing. Finally, we integrated our findings across populations and environments to identify QTLs detected in two or more populations under multiple field conditions, compared them with QTLs reported in the literature over the last 20 years, and used the resequencing information to determine whether the favorable alleles were present in the AfricaRice breeding lines.

## Materials and Methods

### NERICA-L-19 × IR64-Sub1 Population

A population consisting of 445 F_3_-derived F_5_ lines of the NERICA-L-19 (N-L-19; female parent) × IR64-Sub1 (male parent) cross was used in a field trial conducted in two different sites in Liberia during the 2016 wet season (WS). The first site, Lofa, is characterized as a control location because soil iron levels are low and plants show no symptoms of HIA stress. The second site, Suakoko, had sandy loam soil, a pH of 5.2, an estimated Fe content of 489 mg kg^–1^ and was used as an HIA stress location (Table 1). In each site, individual F_5_ lines were replicated three times in an alpha lattice design. Seedlings were raised in a nursery and at 3 weeks of age were transplanted in the field (one seedling per hill with 20 cm between hills). Each plot consisted of two rows (2 m-long) of 10 plants/row. Fertilization and trial management were conducted as described by Sikirou et al. (2018).

**TABLE 1:** Soil characteristics of the different sites with respective iron (Fe) content (source: Sikirou et al., 2018).

Locations	Condition	Texture	pH (H_2_O)	Fe content
Edozhigi, Nigeria	HIA stress	Clay loam	4.3	1230 mg kg^–1^
Suakoko, Liberia	HIA stress	Sandy loam	5.2	489 mg kg^–1^
Vallee du Kou, Burkina Faso	HIA stress	Silt loam	6.1	295 mg kg^–1^
Lofa, Liberia*	Non-stress	n/a	n/a	n/a
Ibadan, Nigeria	Non-stress	Clay	7.0	84 mg kg^–1^

*Soil characteristics not available (n/a) but the site is commonly used as non-stress site.

### NERICA-L-43 × IR64-Sub1 Population

A population consisting of 310 F_5_ lines of the NERICA-L-43 (N-L-43; female parent) × IR64-Sub1 (male parent) cross were used in a field trial conducted in two different sites in Nigeria during the 2017WS. The first site, Ibadan, is characterized as a control location. The second site, Edozhigi, was used as HIA stress location characterized by clay loam soil, pH 4.3 and an Fe content of 1230 mg kg^–1^ (Table 1). In each site, the lines were replicated two times in an alpha lattice design. Seedlings were raised in a nursery and at 3 weeks of age were transplanted in the field (one seedling per hill with 20 cm between hills). Each plot consisted of a single 3 m-long row of 16 plants. Fertilization and trial management were conducted as described by Sikirou et al. (2018).

### Rice Diversity Panel

A collection of 296 rice varieties from the Rice Diversity Panel (Zhao et al., 2011) was evaluated in different West African sites during the 2012 and 2013 wet seasons. As summarized in Supplementary Table S1, the panel includes varieties from five *O. sativa* subpopulations, including 65 *indica* (IND), 49 *aus* (AUS), 7 *aromatic* (ARO), 67 *tropical japonica* (TRJ), 73 *temperate japonica* (TEJ), and 35 accessions classified as *admixed* (ADM, ADM-IND, ADM-JAP) (McCouch et al., 2016). The trials were conducted in three HIA stress sites and one control location; HIA stress sites included two of the same sites as for the bi-parental populations: Edozhigi (Nigeria) 2012WS and Suakoko (Liberia) 2013WS, with a third site in Valle du Kou (Burkina Faso) 2013WS (Table 1). A lowland rice field in Ibadan (Nigeria) was used as the control environment during 2012WS and 2013WS. Each trial consisted of two replications per genotype arranged in an alpha lattice design. Seeds were sown in the nursery and transplanted at 3 weeks of age into a puddled field with plots consisting of a single 3 m-long row with 16 plants (one plant per hill with 20 cm between hills). Fertilization and trial management were conducted as described by Sikirou et al. (2018).

### Scored Traits

Four traits were evaluated in each trial according to IRRI’s Standard Evaluation System for Rice (IRRI, 2002). Days to flowering (FLW) was recorded when 50% of the plants in the plot started to flower. Plant height (PHT) was measured at harvest time as the average distance from the ground to the tip of the longest panicle of three randomly selected plants per plot. For the bi-parental populations, grain yield (GY) was determined for each plot and converted to the weight (kg) of filled grains per hectare (ha) adjusted to 14% moisture content. For RDP1, GY was calculated as the weight (grams) of filled grains per plot adjusted to 14% moisture content. Leaf bronzing score (LBS) was expressed on a 0-9 scale, where 0 = healthy green leaf, no visible symptoms, 1 = reddish-brown spots or orange discoloration on tips of older leaves, 3 = older leaves reddish-brown, purple, or orange yellow, 5 = many leaves discolored, 7 = most leaves discolored or dead, and 9 = all leaves dead. LBS was scored 63 (LBS63) and 84 (LBS84) days after seeding for the two populations derived from bi-parental crosses. For the Rice Diversity Panel, only LBS84 was scored. The percentage of grain yield loss (GY-loss) was calculated as (GY_CON_ – GY_FE_)/(GY_CON_)^∗^100, where GY_CON_ = GY in the control site, and GY_FE_ = GY in a HIA stress site(s).

### Statistical Analysis

Statistical analysis of the data was conducted using R statistical software (version 3.4.3; The R Foundation for Statistical Computing). For the bi-parental populations, the software “Breeding View” (The Breeding Management System) was used to generate a linear mixed model (with genotypes as fixed effect and replicate and the nested block as random effects) in order to calculate the best linear unbiased estimators (BLUEs) of phenotypic traits (FLW, PHT, GY, and LBS) for each line at each different site. For RDP1, adjusted means (Supplementary Table S1) of each genotype for each trait at each site were generated using a multi-trait Bayesian hierarchical model (Greenberg et al., 2011). Trait values exceeding ± (2.5^∗^St. Dev) from the mean were considered outliers and removed from the analysis. Correlation analysis and graphical matrices were produced using the “corrplot” R package. Box-Cox transformation of non-normally distributed traits was calculated using the “forecast” R package (Hyndman and Khandakar, 2008).

### Genotypic Data for QTL Mapping

The 445 F_5_ lines of the N-L-19 × IR64-Sub1 cross and the 310 F_5_ lines of the N-L-43 × IR64-Sub1 cross were genotyped, together with the parental lines, by Diversity Array Technology (DArT) based on next-generation sequencing (DArTseq^TM^). Briefly, young leaf tissue was harvested from a single parental plant or F_5_ line from screenhouse-grown seedlings at AfricaRice and lyophilized tissue was shipped to DArT Pty Ltd., Canberra, Australia. Genomic DNA was extracted from individual plant samples and genotyped using the DArTseq^TM^ technology (Sansaloni et al., 2010; Ren et al., 2015). Methodology on complexity reduction, cloning, library construction and cleaning are as described in Egea et al. (2017). Amplification fragments were sequenced at 96-plex on the Illumina Hiseq 2500^1^, short reads were aligned to the Nipponbare reference genome (MSUv7), and a total of 10,319 SNPs were called by Diversity Arrays Technology Pty Ltd^2^ for both crosses.

Single nucleotide polymorphisms markers with high missingness (NAs > 30%), low minor allele frequency (MAF < 20%), and that were monomorphic or heterozygous in the parental lines, were subsequently removed from the dataset. The final number of SNP markers available for mapping was 1,767 and 1,897 (Supplementary Dataset S1-S2) for the populations derived from the N-L-19 × IR64-Sub1 and N-L-43 × IR64-Sub1 crosses, respectively. The genome-wide distribution of SNPs is shown in Supplementary Figure S1A (N-L-19 × IR64-Sub1) and Supplementary Figure S1B (N-L-43 × IR64-Sub1). The number of markers per chromosome, together with the number of shared markers (SNPs with the same physical position) across the two populations, are summarized in Supplementary Table S2.

### QTL Mapping of the Bi-parental Populations

Boxplots and distribution density of residuals for FLW, PHT, GY, GY-loss, LBS63 and LBS84 in the two populations are reported in Supplementary Figure S2 (N-L-19 × IR64-Sub1) and in Supplementary Figure S3 (N-L-43 × IR64-Sub1). SNP maps were initially transformed by an R script from the nucleotide-based hapmap format to an ABH-based format, where “A” = IR64-Sub1 allele, “B” = NERICA allele, “H” = heterozygote, and N = missing. The “ABHgenotypeR” R package (Furuta et al., 2017) was then used for imputation of missing SNPs (function *imputeByFlanks*) and for correction of genotyping errors (functions *correctUnderCalledHets* and *correctStretches* with maxHapLength = 1). The same package was used to calculate and visualize the allele frequencies of SNPs per chromosome (Supplementary Figures S4, S5).

Quantitative trait locus analysis was conducted using the “R/qtl” R package (Broman et al., 2003). Before performing the QTL scans, we imputed the remaining missing SNPs using a hidden Markov model repeated 100 times (package function *sim.geno)*. Genetic maps were calculated by converting the marker physical distances into recombination fractions (Kosambi mapping function) using the package *est.map* function. Finally, the underlying genotype probabilities were calculated using the package *calc.genoprob* function (Kosambi mapping function). For each trait, QTL scans were conducted by composite interval mapping (*cim* function of the package) considering only markers on chromosomes different from the marker under test (window = Inf) as covariates (n.marcovar = 8). 1,000 randomized permutations were used to determine the LOD threshold for significance. For each significant QTL, position and percentage of phenotypic variance explained (PVE) by the top marker is reported along with the LOD ± 1.5 confidence interval (calculated using the package *lodint* function). If two or more significant QTLs were identified on a single chromosome by composite interval mapping, their interaction was tested by fixing the most significant marker as a covariate and re-running a QTL scan based on interval mapping (*scanone* function of the package with Haley-Knott regression) for that chromosome only.

### Phenotypic Data Analysis and Genome-Wide Association Mapping of the RDP1 Population

Grain yield, GY-loss and LBS84 were the traits used in association mapping. Trait values, cleaned of outliers, were Box-Cox transformed before being used in association mapping analysis. Boxplots (untransformed data) and distribution density (residuals of Box-Cox transformed data) for each trait in each site are reported in Supplementary Figures S6-S10. Genome-wide association studies (GWAS) were performed using a linear-mixed model in EMMAX (Kang et al., 2010) which corrects for population structure by including a kinship matrix (IBS matrix) as a covariate. EMMAX also provides an estimate of the PVE by the IBS matrix (marker-based heritability, *h*^2^). The 296 RDP1 accessions were previously genotyped using a 700K SNP High Density Rice Array (HDRA) (McCouch et al., 2016)^3^. GWA analyses were conducted using the entire RDP1 panel (*AllPOP*, 296 accessions) as well as subsets corresponding to the *INDICA* (*aus*, *ind*, *adm-ind*) and *JAPONICA* (*trj*, *tej*, *aro*, *adm-jap*) varietal groups, as previously described by McCouch et al. (2016). The genotype data was filtered to include only lines with phenotypes for the traits of interest, SNPs with MAF ≥ 0.05 for each of the four subpopulations (*aus*, *ind, trj* and *tej*), and 30% maximum missing data, following the approached used by McCouch et al. (2016). PCA (*prcomp* function in the “stats” R package) was conducted using 20% of randomly selected SNPs to quantify subpopulation structure in *AllPOP*, *INDICA* and *JAPONICA* (Supplementary Figure S11). Three PC covariates were added to the linear-mixed model when analyzing *AllPOP*, and one PC covariate was added when analyzing the *INDICA* and *JAPONICA* groups McCouch et al. (2016). GWAS results are presented as Manhattan and Quantile-Quantile plots using the “qqman” R package. A significance threshold of *p* < 0.00001 (i.e., -log_10_ *p* ≥ 5.0) was used to identify the most interesting marker-trait associations. A QTL region was defined by the presence of a SNP with significance of -log_10_ *p* ≥ 5.0 and at least two additional SNPs showing significance of -log10 *p* > 3.5 and located <200 kb from the most significant marker. For each QTL, the presence of multiple SNPs with high significance in a restricted region helped to control for Type 1 errors.

### Sequencing of AfricaRice Breeding Lines

Thirty two breeding lines (21 *O. glaberrima*, 10 *O. sativa*, and 1 NERICA) used to improve HIA stress, drought and submergence tolerance in the AfricaRice program were sequenced on an Illumina NextSeq500 sequencer at Cornell University to an average depth of ∼5x genome coverage (Supplementary Table S3). The Illumina sequencing reads were aligned to the Nipponbare reference genome (MSUv7) using BWA software (McKenna et al., 2010) and SNP calling was done with the GATK Unified HaplotypeCaller algorithm (DePristo et al., 2011; Van der Auwera et al., 2013; Poplin et al., 2018) using an in-house pipeline.

The sequencing dataset from the AfricaRice breeding lines was integrated with imputed data from the RDP1 using the Rice-RP dataset described by Wang et al. (2018). Annotation for the *DTH3* gene^4^ was accessed from The Rice Annotation Project Database (RAP-DB^5^).

### Re-evaluation of High Yielding RDP1 Accessions

The best yielding accessions of RDP1, based on evaluation of GY under conditions associated with HIA stress (Suakoko and Vallee du Kou) and non-stress (Ibadan) during the 2012-2013WS, along with the parents, NERICA-L-19, NERICA-L-43, and IR64-Sub1, and five lines from the collection of elite AfricaRice breeding materials, were grown again during the 2017WS in Edozhigi and Suakoko (HIA stress soils) as well as in Ibadan (control conditions), and evaluated for LBS84 and GY (calculated as kg/ha adjusted to 14% moisture content, as for the bi-parental field trials) using two replications per genotype in an alpha lattice design. Each plot consisted of a single 3 m-long row with 15 plants. Planting, transplanting, fertilization and trial management were conducted as described by Sikirou et al. (2018). Best linear unbiased estimators (BLUEs) of LBS84 and GY were calculated (following the same procedure described for the bi-parental field trials) for each line at each different site and are provided in Supplementary Dataset S3.

### Inventory of QTLs Related to HIA Stress Tolerance in Rice Reported in the Literature

An inventory of QTLs reported in 13 independent publications on rice tolerance to HIA stress (Wu et al., 1997, 1998, 2014; Wan et al., 2003; Ouyang et al., 2007; Dufey et al., 2009, 2012, 2015; Shimizu, 2009; Fukuda et al., 2012; Matthus et al., 2015; Zhang et al., 2017; Diop et al., 2020) was created. The integration of RFLP and SSR markers from older versions of rice linkage maps required that we determine the physical positions of these markers on the current rice genome assembly (MSUv7, equivalent to IRGSP v1.0). To do this, we used SSR primer and RFLP clone-sequence information (from the Gramene Markers Database^6^) to identify the physical position of these markers on the Nipponbare MSUv7 genome using the Gramene Blasting Tool^7^. In cases where a marker’s primers and/or sequence was not available, the closest flanking marker reported in the study was used to localize the QTL. An internal lift-over chain conversion algorithm was used to convert the previously reported positions of SNP markers aligned to the Nipponbare MSUv6 assembly to correctly locate them on the MSUv7 Nipponbare genome^8^.

## Results and Discussion

### Phenotypic Response and Relationships Among Traits

In this study, two bi-parental populations and one rice diversity panel were evaluated for phenotypic performance in a series of experiments conducted in different West African field environments. The environments included natural “hotspots” for HIA stress characterized by acidic soils with high concentrations of soil Fe and control (non-toxic) locations (Table 1).

#### Bi-parental Populations

**FIGURE 1:**
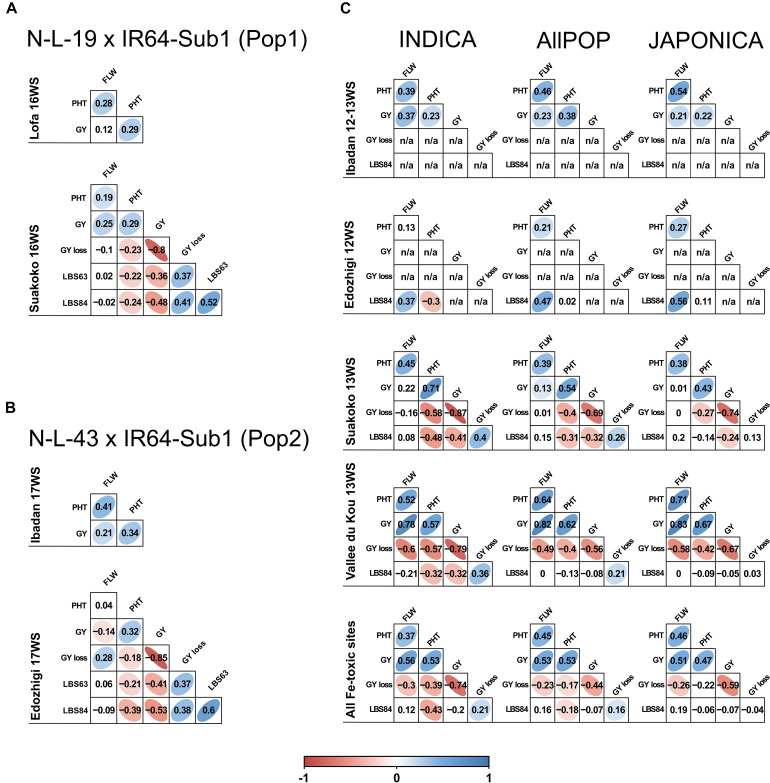
Correlations between traits in the N-L-19 × IR64-Sub1 (Pop1) and N-L-43 × IR64-Sub1 populations (Pop2) and RDP1 panel. Correlations between traits scored in the field trials conducted in different West African sites during the 2012, 2013, 2016 and 2017 for Pop1 **(A)**, Pop2 **(B)** and RDP1 **(C)**. For RDP1, correlations are displayed for all *AllPOP*, *INDICA* and *JAPONICA* varietal groups. Lofa and Ibadan: control sites; Suakoko, Edozhigi and Vallee du Kou: HIA stress sites. White cells indicate a non-significant (*p* > 0.01) correlation value. The strength of significant (*p* < 0.01) correlations is indicated by values and colors (red if negative and blue if positive). FLW: days to flowering; PHT: plant height; GY: grain yield; GY loss: grain yield loss; LBS63: leaf bronzing score at 63 days after sowing; LBS84: leaf bronzing score at 84 days after sowing; n/a: not available.

The bi-parental populations utilized for QTL mapping were derived from crosses between two HIA stress tolerant NERICA lines (female parents), N-L-19 (Pop1) and N-L-43 (Pop2), and IR64-Sub1, an HIA stress susceptible *indica* parent (male). Pop1 was evaluated in Liberia, where Suakoko represented the HIA stress site and Lofa represented the control location, while Pop2 was grown in Nigeria, with Edozhigi representing the HIA stress site and Ibadan the control location. Under HIA stress conditions, stress-induced leaf bronzing (LBS) symptoms were always negatively correlated with grain yield (GY), positively correlated with GY-loss, and similar values were observed in the two populations (Figures 1A,B). Under stress, Pop1 and Pop2 also shared similar negative correlations between LBS and plant height (PHT), indicating greater tolerance (lower stress symptoms) in taller plants. The two populations also displayed a similar, positive correlation between PHT and GY which was evident under both control and stress conditions, indicating higher grain yield in taller plants, regardless of stress. The only marked difference between the two populations was that Pop 2 displayed a negative correlation between FLW and GY under stress (Figure 1B), while the correlation was positive in Pop1 (Figure 1A). This difference is likely associated with the fact that Pop2 exhibited early flowering in response to stress, which was not observed in Pop1.

Despite the similarity of the correlation coefficients between the two populations, they displayed very different mean yield reductions under stress; 63% GY-loss in Pop1 versus 19.3% GY-loss in Pop2, compared to their respective control locations (Table 2). This may be related to the fact that N-L-19 (parent of Pop1) is a taller plant than N-L-43 (parent of Pop2) (Venuprasad, pers. comm.). The strong GY-loss in Pop1 was not accompanied by a reduction in PHT, and by only a marginal reduction in FLW (∼5 days). In contrast, Pop2 displayed a marked stress-induced reduction in both PHT (∼10 cm) and FLW (∼24 days). These results suggest that the two populations had very different responses to stress in the two locations. Pop2 demonstrated a stress-escape strategy based on an early shift of phenology (earlier FLW and shorter PHT) that, in turn, was associated with relatively low LBS symptoms (LBS63 = ∼2 and LBS84 = ∼3) and loss of GY. In contrast, the phenology of Pop1 was only marginally altered by the stress, resulting in greater yield loss and more severe LBS symptoms (LBS63 = 4 and LBS84 = ∼5) (Table 2).

**TABLE 2:** Trait performance of N-L-19 × IR64-Sub1 (Pop1) and N-L-43 × IR64-Sub1 (Pop2) populations and RDP1 in the West African sites.

Population	Varietal group	Condition	Site	Country	Wet season	FLW (days)^a^	PHT (cm)^b^	GY (kg/ha or grams/plot)^c^	GY loss (%)^d^	LBS63 (score)^e^	LBS84 (score)^e^
N-L-19 × IR64-Sub1 (Pop1)	n/a	Control	Lofa	Liberia	2016	97.6 ± 5.7	91.4 ± 11.9	2354 ± 536	n/a	n/a	n/a
	n/a	HIA stress	Suakoko	Liberia	2016	93.0 ± 5.2	91.1 ± 10.6	839 ± 348	63.0 ± 16.2	4.0 ± 0.7	5.1 ± 0.8
N-L-43 × IR64-Sub1 (Pop2)	n/a	Control	Ibadan	Nigeria	2017	115.6 ± 5.2	98.8 ± 6.9	3221 ± 421	n/a	n/a	n/a
	n/a	HIA stress	Edozhigi	Nigeria	2017	91.5 ± 5.9	88.5 ± 6.3	2580 ± 348	19.3 ± 14.4	1.8 ± 0.8	2.9 ± 1.3
RDP1	All POP	Control	Ibadan	Nigeria	2012-13	83.6 ± 8.1	116.6 ± 15.3	158.25 ± 69.10	n/a	n/a	n/a
	INDICA	Control	Ibadan	Nigeria	2012-13	84.2 ± 7.1	121.8 ± 13.9	212.81 ± 56.85	n/a	n/a	n/a
	JAPONICA	Control	Ibadan	Nigeria	2012-13	83.3 ± 8.8	113.2 ± 15.0	117.39 ± 43.48	n/a	n/a	n/a
RDP1	All POP	HIA stress	Edozhigi	Nigeria	2012	84.7 ± 9.0	99.7 ± 9.5	n/a	n/a	n/a	1.5 ± 0.2
	INDICA	HIA stress	Edozhigi	Nigeria	2012	85.5 ± 7.8	104.6 ± 7.1	n/a	n/a	n/a	1.6 ± 0.2
	JAPONICA	HIA stress	Edozhigi	Nigeria	2012	84.4 ± 9.8	96.3 ± 9.3	n/a	n/a	n/a	1.5 ± 0.2
RDP1	All POP	HIA stress	Suakoko	Liberia	2013	82.4 ± 11.2	75.7 ± 7.1	9.15 ± 5.69	93.5 ± 4.1	n/a	4.4 ± 0.6
	INDICA	HIA stress	Suakoko	Liberia	2013	84.5 ± 10.2	75.2 ± 7.5	10.49 ± 6.78	95.1 ± 3.1	n/a	4.5 ± 0.6
	JAPONICA	HIA stress	Suakoko	Liberia	2013	80.9 ± 11.8	76.0 ± 6.9	8.27 ± 4.50	92.5 ± 4.1	n/a	4.5 ± 0.5
RDP1	All POP	HIA stress	Vallee du Kou	Burkina Faso	2013	84.8 ± 6.8	71.5 ± 6.7	17.23 ± 8.05	88.2 ± 5.4	n/a	3.1 ± 0.5
	INDICA	HIA stress	Vallee du Kou	Burkina Faso	2013	86.5 ± 5.4	72.1 ± 6.4	20.40 ± 8.42	89.8 ± 4.3	n/a	3.3 ± 0.5
	JAPONICA	HIA stress	Vallee du Kou	Burkina Faso	2013	83.1 ± 7.1	70.6 ± 6.6	14.46 ± 6.65	87.3 ± 5.6	n/a	3.0 ± 0.4
RDP1	All POP	HIA stress	All sites	n/a	2012-13	84.3 ± 6.8	82.6 ± 5.3	13.47 ± 5.46	90.8 ± 3.8	n/a	3.1 ± 0.3
	INDICA	HIA stress	All sites	n/a	2012-13	85.3 ± 6.6	84.1 ± 5.0	15.80 ± 5.97	92.3 ± 2.9	n/a	3.1 ± 0.4
	JAPONICA	HIA stress	All sites	n/a	2012-13	83.5 ± 6.9	81.4 ± 5.0	11.62 ± 4.20	89.5 ± 4.3	n/a	3.0 ± 0.2

*^a^Days to flowering. ^b^Plant height. ^c^Grain yield in kg/ha for the bi-parental populations and in grams/plot for the RDP1. ^d^Grain yield loss. ^e^Leaf Bronzing Score at 63 and 84 days after seeding.*

#### RDP1

The 296 accessions of the *O. sativa* GWAS panel (RDP1) were grown for one season in Ibadan (in 2011) under favorable conditions to amplify seed and all performed reasonably well. The panel was subsequently grown in three HIA stress hotspots, in Nigeria (Edozhigi, 2012WS), Liberia (Suakoko, 2013WS) and Burkina Faso (Vallee du Kou, 2013WS). In Edozhigi, GY and GY-loss could not be determined because the highly stressful conditions (Table 1) were responsible for complete yield loss for almost all lines, such that the population was not harvested (Table 2). HIA stress greatly affected yield performance with a mean GY-loss of ∼90% or higher in HIA stress sites compared to control (Table 2). Overall, HIA stress did not affect the mean FLW phenology of the panel, but it strongly reduced PHT. Despite the high Fe content of the soil, PHT reduction was less marked in Edozhigi, and LBS symptoms were less severe than in Suakoko and Vallee du Kou. These observations, combined with the extreme yield loss during the 2012WS in Edozhigi, suggested an unusual source of acute stress, rather than HIA stress alone, at that site. Additionaly, these results further highlight the complexity of the syndrome associated with anaerobic conditions in acid soils which is likely determined by a wide range of nutritional disorders (toxicities and deficiencies) driven by site specific soil levels of a variety of micronutrients, in addition to high Fe levels (Kochian et al., 2004; Mahender et al., 2019). It is mainly for this reason that Diop et al. (2020) proposed the terminology “high iron-associated (HIA)” stress as more appropriate for describing the syndrome under field conditions.

When RDP1 varieties representing the two main varietal groups of *O. sativa*, *INDICA* and *JAPONICA*, were compared, they showed similar results for GY-loss, reduced PHT and LBS symptoms in Suakoko and Vallee du Kou, but displayed very different GY performance. *INDICA* always showed higher GY than *JAPONICA*, and better GY values were observed in Vallee du Kou compared to Suakoko (Table 2). A one-way ANOVA indicated that varietal group explained 41.3% of GY variation in the control site (Ibadan) and 14.4% of GY variation in the HIA stress sites (4.7% in Suakoko and 14.1% in Vallee du Kou). The presence of a marked GY difference, especially in the control site (*INDICA* GY = 212.81 grams/plot versus *JAPONICA* GY = 113.2 grams/plot), indicates that *INDICA* varieties are generally better adapted to West African lowland environments than are *JAPONICA* varieties.

The 296 accessions of the panel displayed a similar overall trend in terms of trait correlations under HIA stress conditions, but with site- and varietal group-specific differences (Figure 1C). While the lack of yield in Edozhigi eliminated that site from consideration, PHT was positively correlated with GY (negatively with GY-loss) in all the varietal groups at the other two sites. The positive correlations between PHT and GY were stronger in the HIA stress sites than in control conditions. This suggests that better GY performance is associated with taller plants. PHT was also negatively correlated with LBS84 symptoms in both Suakoko and Vallee du Kou in the *INDICA* varietal group (though the difference was not significant in *JAPONICA*). In Suakoko, LBS84 was negatively correlated with GY (and positively with GY-loss) for both *INDICA* and *JAPONICA*, but the negative correlation was significant for only *INDICA* in Vallee du Kou. In the latter location, FLW was strongly and positively correlated with GY (negatively with GY-loss) while the same correlations were not significant in Suakoko. Taken together, these results confirm the overall detrimental effect of typical HIA stress symptoms (measured as LBS) on GY (Audebert and Fofana, 2009; Diop et al., 2020), and highlight the marked differences in performance between varietal groups (*INDICA* more tolerant than *JAPONICA*) and West African HIA stress sites (Sikirou et al., 2015, 2016).

### QTL Analysis of the Two Bi-parental Populations

**FIGURE 2:**
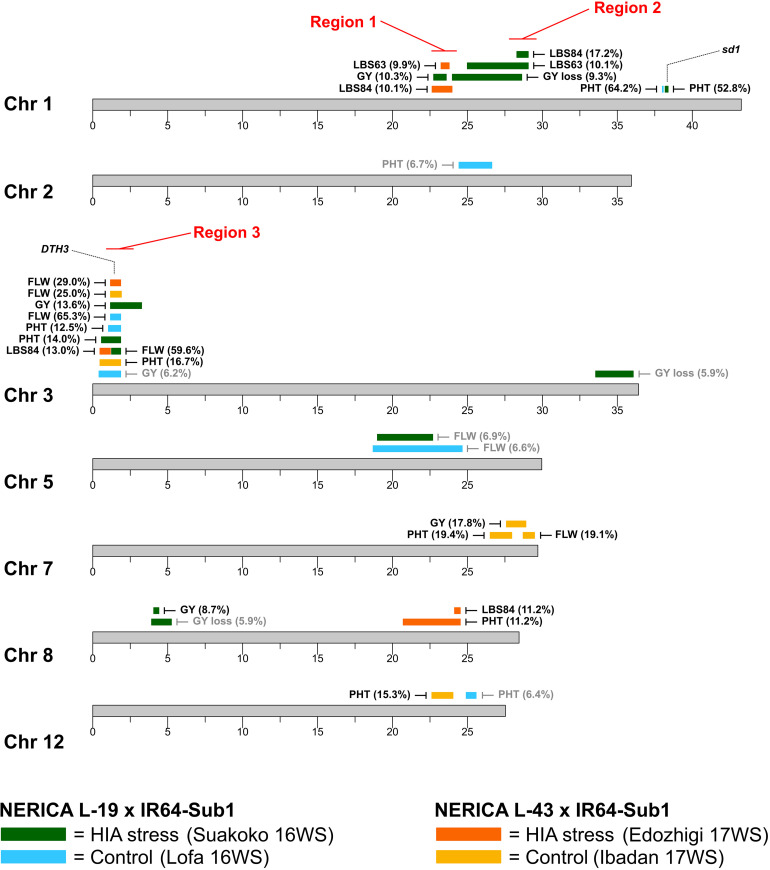
Location of QTLs mapped in N-L-19 × IR64-Sub1 (Pop1) and N-L-43 × IR64-Sub1 populations (Pop2). Percentage of phenotypic variance explained (PVE) by the top marker of each QTL is reported in brackets (gray color if PVE < 7.5%). Positions of the semi-dwarf gene on chromosome 1 (*sd1*) and of the heading date gene on chromosome 3 (*DTH3*) are displayed. Chromosome regions showing high density of overlapping QTLs (≥3) related to HIA stress tolerance are indicated in red. FLW: days to flowering; PHT: plant height; GY: grain yield; GY loss: grain yield loss; LBS63: leaf bronzing score at 63 days after sowing; LBS84: leaf bronzing score at 84 days after sowing.

A total of 31 significant QTLs were detected for the six traits evaluated under control and HIA stress locations for Pop1 (19 QTLs) and Pop2 (12 QTLs), as summarized in Figure 2 and Table 3.

**TABLE 3:**
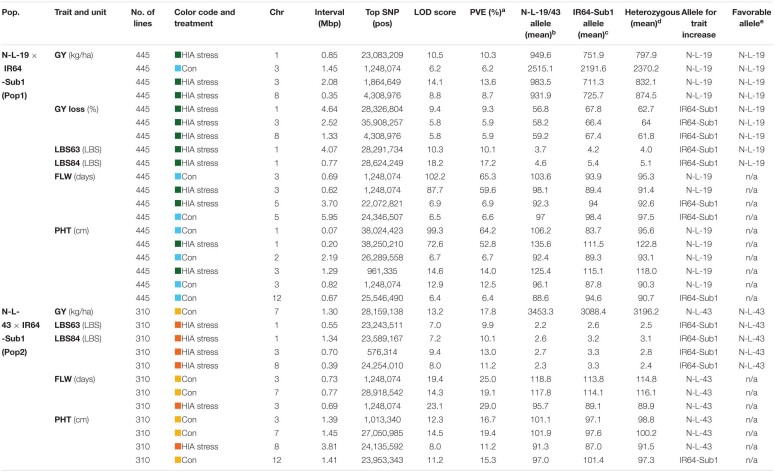
Significant QTLs identified for the traits scored in N-L-19 × IR64-Sub1 (Pop1) and N-L-43 × IR64-Sub1 (Pop2) populations.

*^a^Phenotypic variance explained (PVE) by the top marker of the QTL. ^b^Trait mean value for the group of accessions carrying both NERICA alleles at the locus of the top marker of the QTL. ^c^Trait mean value for the group of accessions carrying both IR64-Sub1 alleles at the locus of the top marker of the QTL. ^d^Trait mean value for the group of accessions carrying heterozygous alleles at the locus of the top marker of the QTL. ^e^Parent allele conferring tolerance to HIA stress. The favorable allele is associated to higher GY and lower GY loss, LBS63 and LBS84. For FLW and PHT it is not possible to determine (n/a: not applicable) the favorable allele.*

Three main chromosomal regions (Regions 1, 2, and 3) carried overlapping QTLs (≥3 QTLs) associated with plant response to stress (Figure 2 and Supplementary Figure S12). Regions 1 and 2 are linked on chromosome 1, mapping between ∼22.6 Mbp and ∼28.6 Mbp. Region 1 encompasses three QTLs identified in HIA stress sites: one for grain yield (Pop1), and two for LBS63 and LBS84 (Pop2). The NERICA alleles (N-L-19 for Pop1 and N-L-43 for Pop2) are favorable in both cases (lower LBS, higher yield) (Table 3), though the most significant markers for each trait explain a relatively low percent of the observed phenotypic variation (PVE = ∼10%).

Region 2 is comprised of three overlapping QTLs, all identified in the HIA stress site (Pop1), one for GY-loss and the other two for LBS63 and LBS84 (Figure 2 and Supplementary Figure S12). The PVE of the GY-loss and LBS63 QTLs is ∼10% while the most significant marker for the LBS84 QTL explains 17.2% of the phenotypic variation. The NERICA alleles (N-L-19) at these the QTLs in Region 2 are again responsible for better trait performance (lower GY-loss and LBS) (Table 3). Interestingly, two major-effect QTLs for PHT were identified in both the HIA stress and control sites on chromosome 1 (Pop1), with the most significant markers explaining 52.8% and 64.2% of trait variation, respectively, and the NERICA allele (N-L-19) conferring greater PHT in both environments (Table 3). These PHT QTLs co-localize with the *SD1* gene (*OsGA20ox2*, Os01g0883800); a recessive allele at this locus is responsible for the semi-dwarf phenotype of modern rice varieties (Monna et al., 2002; Sasaki et al., 2002; Spielmeyer et al., 2002), including IR64.

Region 3 is located on the short arm of chromosome 3 and displays ten overlapping QTLs (Figure 2). Among them two FLW QTLs were detected in each of the two populations in both HIA stress and control sites. The top markers for these FLW QTLs explain ∼60-65% of the phenotypic variation for Pop1, and ∼25-30% for Pop2, with the NERICA alleles conferring later FLW in all cases (Table 2). The four overlapping FLW QTLs co-localize with *DTH3* (Os03g01122600; also known as *OsMADS50*), a gene known as an important flowering activator in rice (Lee et al., 2004), and previously associated with variation in flowering time in *O. glaberrima* (Bian et al., 2011). Region 3 also includes three PHT QTLs (two in Pop1 and one in Pop2; ∼13-16% PVE), where the NERICA alleles are again associated with greater PHT (Table 3). Apart from the FLW and PHT QTLs, Region 3 also includes two GY QTLs, one identified under HIA stress and one under control conditions in Pop1, and one LBS84 QTL identified in Pop2 (Figure 2 and Supplementary Figure S12). The NERICA alleles were favorable in all cases. N-L-19 (Pop1) alleles conferred higher GY values in both HIA stress and control sites, with higher PVE (13.6%) under HIA stress than under control conditions, and N-L-43 (Pop2) alleles were associated with lower LBS symptoms (PVE = 13%) (Table 3).

Detailed information on the QTL mapping results for the 31 QTLs are provided in Supplementary Figures S13-S21 (Pop1) and Supplementary Figures S22-S28 (Pop2).

### Genome-Wide Association (GWA) Mapping of HIA Stress Tolerance in the RDP1 Panel

A total of 68 significant (-log_10_ *p* ≥ 5.0) QTLs were identified by GWA mapping of GY, GY-loss and LBS84 performance in the *O. sativa* RDP1 across three HIA stress sites (Edozhigi, Suakoko, and Valle du Kou) and the control site (Ibadan) during 2012WS and 2013WS. QTL locations are summarized in Figure 3 and further details are provided in Supplementary Table S4. Manhattan and Quantile-Quantile plots of the GWA mapping Supplementary Figures S29-S39. Allele frequencies in the panel as a whole and in each of the *O. sativa* subpopu results are provided in lations are provided in Supplementary Figures S40-S42 for GY QTLs, Supplementary Figures S43-S45 for GY-loss QTLs, and Supplementary Figures S46-S49 for LBS84 QTLs.

**FIGURE 3:**
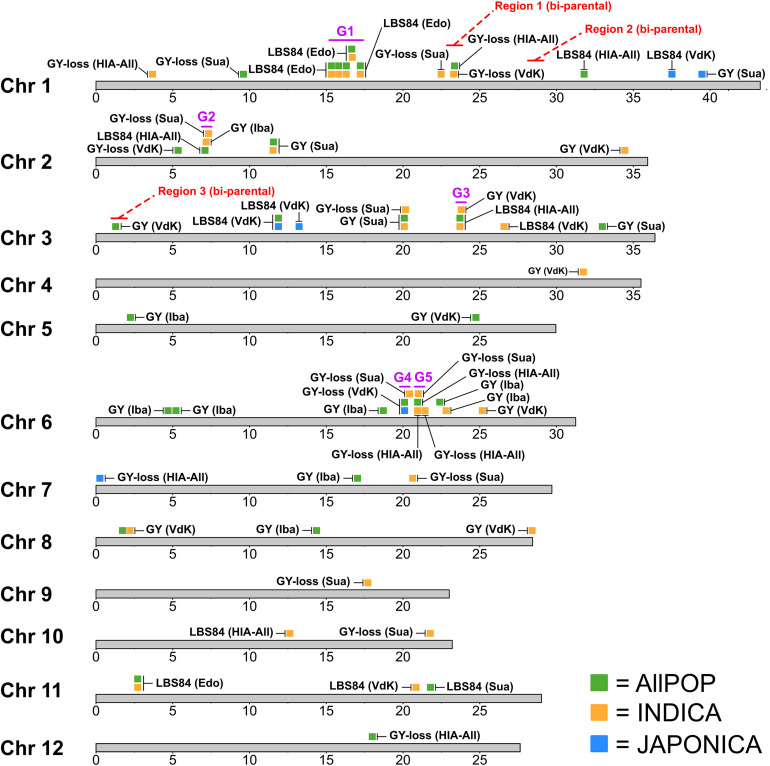
Location of GWA-QTLs mapped in RDP1. QTL positions indicated based on the bp coordinates of the most significant SNP (msSNP ± 200 kb). Red letters showing “Region 1-3 (bi-parental)” refer to the bi-parental QTL regions indicated in Figure 2. Purple letters showing “G1-G5” refer to the regions with clusters of GWA-QTLs in HIA stress sites. GY: grain yield; GY-loss: grain yield loss; LBS84: leaf bronzing score at 84 days after seeding. Iba: Ibadan; Edo: Edozhigi; Sua: Suakoko; VdK: Vallee du Kou; HIA-All: all HIA stress sites. QTLs detected in *AllPOP* shown in green; in *INDICA* shown in orange; in *JAPONICA* shown in blue.

Thirty QTLs were mapped in *AllPOP*, 32 were mapped in the *INDICA* varietal group, and only six in *JAPONICA* (Figure 3). In the control site (Ibadan), a total of nine QTLs were mapped for GY; seven were detected in *AllPOP*, two in *INDICA* and none in *JAPONICA* (Figure 3 and Supplementary Table S4). The difference in the number of QTLs detected under both stress and control conditions in the two varietal groups suggests that *INDICA* has more genetic variation associated with yield performance in West African environments, and/or a higher frequency of alleles with large effect, than *JAPONICA*. This hypothesis is further supported by the higher marker-based heritability in *INDICA* (av. *h*^2^: 0.61) than in *JAPONICA* (av. *h*^2^: 0.12) (Supplementary Table S4). Further, the deleterious alleles at the *AllPOP* GY QTLs in Ibadan were almost exclusively associated with *JAPONICA* varieties (Supplementary Figure S40), consistent with the previously described higher GY performance of *INDICA* than *JAPONICA* varieties in West African lowland environments (Table 2).

Fifty-nine QTLs (87%) were mapped in HIA stress sites (12 in Edozhigi, 16 in Suakoko, 19 in Vallee du Kou, and 12 in All HIA stress sites). While GWA-QTLs were found distributed across the genome, we identified five chromosomal regions containing clusters of QTLs (G1-G5 in Figure 3).

The first region (G1) contains 10 Edozhigi QTLs clustered on chromosome 1 (15.1-17.5 Mbp). The Edozhigi site was problematic because virtually all plants failed to yield, and marker-based heritability for the QTLs detected in that location (all for LBS84) was extremely low (*h^2^* = 0.06) (Supplementary Table S4). Furthermore, there was no signal from any of the other HIA stress sites in that genomic region. Thus, we concluded that the Edozhigi-specific QTL cluster region on chromosome 1 was not robust, and we eliminated it from further consideration as a potential breeding target.

The other four regions all contained ≥ 3 GWA-QTLs that overlapped for different traits and sites, and we examined them further to explore their potential as future breeding targets. G2 is on chromosome 2 (6.9-7.5 Mbp) and is comprised of three QTLs, one for LBS84 (mapped in *AllPOP* across HIA stress sites), one for GY (in *INDICA* in Ibadan), and one for GY-loss (mapped in *INDICA* in Suakoko) (Figure 3). All three QTLs were detected at -log_10_ *p* = 5.21-5.74 (Supplementary Table S4). The favorable allele of the LBS84 QTL on chromosome 2 was rare overall and found exclusively in twelve *indica* accessions which represents a frequency of 5.4% in *AllPOP* (Supplementary Figure S49). The favorable allele at the GY (Ibadan) QTL was at highest frequency in the *aus* subpopulation (37.1%), but was also detected in 11.2% of *indica* varieties (Supplementary Figure S40), while the favorable allele at the GY-loss (Suakoko) QTL was predominant in *indica* (52.2%) (Supplementary Figure S43).

The third QTL region (G3) is on chromosome 3 (23.5-24.0 Mbp) and, like the region on chromosome 2, includes three QTLs (Figure 3). The same SNP (pos: 23,709,502) was most significantly associated with two QTLs for LBS84, one detected in *AllPOP* and one in *INDICA* across all HIA stress sites (-log_10_ *p* = 5.82 and 5.11, respectively). The third QTL was mapped for GY in *INDICA* in Vallee du Kou (Supplementary Table S4). The favorable alleles at the two LBS84 QTLs on chromosome 3 (in *INDICA* and in *AllPOP*) were rare and found exclusively in nine *indica* accessions (Supplementary Figure S49). At the GY QTL mapped in *INDICA* in Vallee du Kou, the favorable allele was present in both the *aus* (43.3%) and the *indica* (32.7%) subpopulations (Supplementary Figure S42).

The fourth and fifth QTL regions (G4 and G5) are linked on chromosome 6, mapping only 500 kbp apart, and each is comprised of three overlapping QTLs, all mapped for GY-loss (Figure 3). Despite the fact that these two regions did not include LBS84 QTLs, which would link them directly to the HIA stress conditions of the experimental sites, we considered the G4 and G5 regions to be interesting breeding targets because of the positive correlation that RDP1 accessions showed between LBS84 and GY-loss in all the HIA stress sites (Figure 1). This correlation suggests that the GY-loss observed in the RDP1 panel is largely due to the effect of HIA stress conditions.

G4 (19.9-20.6 Mbp) includes two QTLs mapped for GY-loss in *AllPOP* and *JAPONICA* in Vallee du Kou (same msSNP with pos: 20,102,783 and -log_10_ *p* = 5.57 and 5.38, respectively), and a third QTL mapped for GY-loss in *INDICA* in Suakoko (Supplementary Table S4). The favorable allele at the GY-loss QTL mapped in *JAPONICA* in Valle du Kou is more commonly detected in the *tropical japonica* subpopulation (7.7%), and mapping results for GY-loss in *AllPOP* in the same location show that the same (favorable) allele is also present in the *indica* subpopulation, but at a very low frequency (1.4%) (Supplementary Figures S43, S44). The favorable allele at the QTL for GY-loss in Suakoko is found at highest frequency in *indica* (7.8%) (Supplementary Figure S43).

G5 (20.7-21.2 Mbp) includes three QTLs for GY-loss. The first two were mapped in *AllPOP* and in *INDICA* across HIA stress sites with the same most significant SNP (pos: 20,953,952 and -log_10_ *p* = 5.16 and 5.17, respectively), and a third was mapped in *INDICA* in Suakoko (Supplementary Table S4). The favorable alleles at these QTLs were found at low frequencies in both *indica* and *aus* subpopulations (3.5-3.9% in *aus;* 4.9-5.3% in *indica*) (Supplementary Figures S43, S45).

Given that the favorable QTL alleles across regions G2-G5 are all found at highest frequencies in the *INDICA* group, we checked to see which *INDICA* accessions carried the highest number of favorable alleles (Supplementary Table S5). The variety showing the largest number of favorable SNPs across the nine QTLs was Seratoes Hari (*indica* from Indonesia), which carried six favorable alleles. The other accessions displayed mixtures of favorable and unfavorable alleles across the G2-G5 QTL regions. These results highlight the potential to recombine alleles within the *indica* gene pool in order to develop new varieties with improved HIA stress tolerance across West African environments.

### Sequencing of AfricaRice Breeding Lines and Haplotype Analysis of Bi-parental Regions 1, 2, and 3

In both Pop1 and Pop2, the favorable alleles in Regions 1, 2 and 3 were all derived from the NERICA parents (Figure 2 and Table 3). This finding suggests that they were likely inherited from *O. glaberrima* donors. We were interested to explore this hypothesis and to determine whether any of the favorable NERICA alleles were already present in AfricaRice breeding lines and/or in the group of high performing INDICA accessions from the RDP1 panel.

To address these questions, we sequenced 32 AfricaRice breeding lines (which included the Pop1 parents, IR64-Sub-1 and N-L-19, as well as 21 *O. glaberrima* and 9 *O. sativa* varieties) (Supplementary Table S3). The average sequencing coverage of the 32 AfricaRice breeding lines was ∼5x, which made it possible to integrate the SNP genotyping datasets (McCouch et al., 2016^9^) for the RDP1 INDICA varieties (65 *indica*, 6 *admixed-indica*, and 49 *aus*) with the AfricaRice breeding lines. As a result, we were able to examine haplotype structure across the high-density QTL Regions 1, 2, and 3 in both the RDP1 and the AfricaRice breeding lines, and to make inferences about the ancestry of NERICA-derived haplotypes across the three target regions (Supplementary Figure S12). We identified sets of informative SNPs that defined each regional haplotype using the following criteria: (i) clearly differentiated the N-L-19 (HIA stress tolerant) and IR64-Sub1 (susceptible) haplotypes, and (ii) uniformly covered (> 1 SNP per 10kbp) the three QTL regions. We then selected a set of 15 informative markers to graphically summarize the haplotypes. SNP identities and flanking sequences (50bp before and after) are provided in Supplementary Table S6.

**FIGURE 4:**
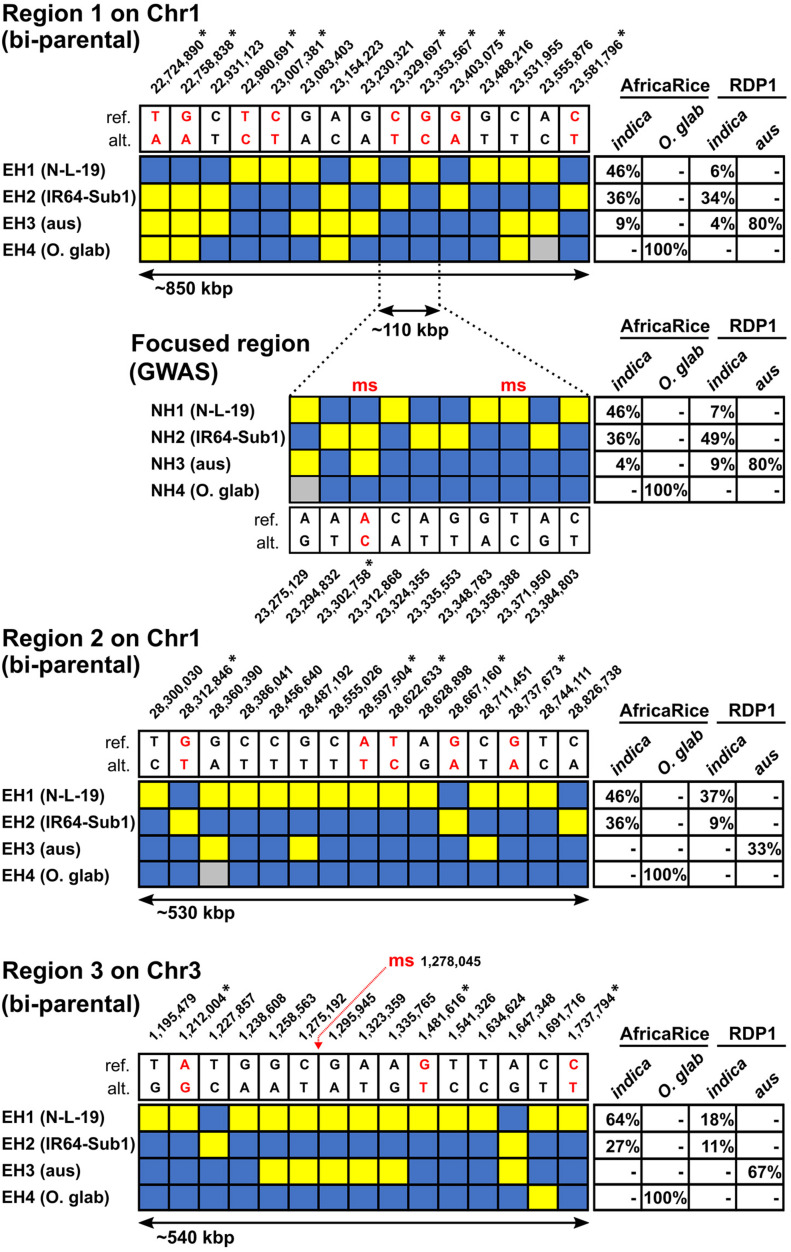
Main haplotypes in the high-density bi-parental QTL regions related to HIA stress tolerance in AfricaRice and RDP1 germplasm. Haplotypes found in Regions 1, 2, and 3 (see Figure 2) constructed based on sets of 15 SNPs each; For Region 1, the haplotypes found in the nested GWAS region based on a set of 10 SNPs are shown; GWA-QTL-msSNPs overlapping with the bi-parental regions are marked by “ms” in red; blue rectangles = reference (cv Nipponbare) allele; yellow = alternate allele; gray = missing data. SNP positions (bp) shown next to reference (ref.) and alternative (alt.) alleles; SNPs predicted to cause non-synonymous substitutions highlighted in red and indicated with an asterisk (^∗^). In Regions 1, 2, and 3, the main “extended” haplotypes found are named EH1 = N-L-19 (female parent), EH2 = IR64-Sub1 (male parent), EH3 = *aus*-like, EH4 = *O. glaberrima*-like. In the nested GWA region, the main “nested” haplotypes found are named NH1 = N-L-19 (female parent), NH2 = IR64-Sub1 (male parent), NH3 = *aus*-like, NH4 = *O. glaberrima*-like. Frequencies of each haplotype in different groups of accessions are indicated in tables where *indica* - AfricaRice group = 10 *indica* and 1 NERICA*; indica* - RDP1 group = 65 *indica* and 6 *admixed-indica*; *O. glab* – AfricaRice group = 21 *O. glaberrima*; *aus* - RDP1 group = 49 *aus* lines (see Supplementary Tables S1, S3). The complete absence of a haplotype in a group is indicated by (-).

Figure 4 summarizes the results of haplotype analysis across the three high-density QTL regions identified in the bi-parental populations; the regions ranged in size between 530 – 850 kbp. Four main haplotypes were identified in each region. EH1 is carried by N-L-19 and is referred to as the “N-L-19 haplotype,” EH2 is carried by IR64-Sub1 at each QTL, and is named as such, EH3 is carried by the majority of RDP1 *aus* lines and is referred to as “*aus*,” and EH4 is an *O. glaberrima-*specific haplotype carried by all 21 sequenced *O. glaberrima* lines from the AfricaRice breeding program, referred to as *O. glab* (Figure 4). Based on these well differentiated haplotypes, genomic regions introgressed from either *O. glaberrima* or *aus* into an *indica* background (i.e., IR64-Sub1) could be easily recognized.

Using this approach, we discovered that EH1, the N-L-19 haplotype associated with HIA stress tolerance in Regions 1, 2, and 3, was not inherited from the *O. glaberrima* parent, TOG5681 (included in the sequenced AfricaRice breeding lines, Supplementary Table S7), but rather traces its ancestry to an unusual group of *indica* varieties. This was surprising considering that the known pedigree of N-L-19 derives from the cross TOG5681/3^∗^IR64 (Sié, 2008). We also examined the three regions to determine whether there was any evidence of an *aus* introgression in the IR64 background, given that the *Sub1* gene originated in an *aus* landrace, FR13A (Septiningsih et al., 2009). We were able to discard this hypothesis, as EH2 perfectly matched genomic information that we had for IR64 across all three QTL regions, based on several independent samples of IR64, including one in the RDP1 panel (Supplementary Table S7). Upon close examination, we discovered that EH1 is well represented in the AfricaRice breeding lines, though it represents a rare haplotype among *indicas* in the *O. sativa* RDP1 panel, while EH2 is a common *indica-*specific haplotype. Neither EH1 nor EH2 were present in any *aus* or *O. glaberrima* accessions (Figure 4 and Supplementary Table S7), nor were they ever found in *JAPONICA* accessions from the RDP1 diversity panel (data not shown).

This discovery led to the conclusion that an undocumented outcross must have occurred during the development of N-L-19, presumably with one (or more than one) *indica* variety that is genetically differentiated from IR64 and considerably more tolerant to HIA stress than IR64 (as it is the source of the HIA stress resistant alleles in all three regions). The unidentified *indica* parent likely represents a variety that was planted in close proximity during development of the NERICA lines at AfricaRice, similar to the situation reported by Asante et al. (2010) related to the aromatic phenotype in NERICA1. In keeping with this hypothesis, Ndjiondjop et al. (2008) observed that N-L-19 displayed one of the highest percentages (24.3%) of non-parental alleles among the NERICA lowland lines. In this context, while the presence of non-parental alleles in NERICA lines has been documented for more than a decade (Semagn et al., 2007; Ndjiondjop et al., 2008), this is the first time that non-parental NERICA introgressions (Regions 1, 2 and 3) associated with an important phenotype (HIA stress tolerance) are discovered and characterized by comparing them to haplotypes present in *O. sativa* and *O. glaberrima* lines.

As summarized in Figure 4, the N-L-19 *indica* haplotypes associated with genetic tolerance (EH1) are found at higher frequencies in AfricaRice *indica* accessions for all the three regions, compared to the Asian *indica* accessions represented in the RDP1 (46% vs 6% in Region 1, 46% vs 37% in Region 2; 64% vs 18% in Region 3, respectively). The difference was particularly striking for Regions 1 and 3 and is consistent with positive selection for HIA stress tolerance by AfricaRice breeders.

### Integration of Bi-parental and GWAS Mapping Results

To investigate the relationship between the genetic determinants of HIA stress tolerance identified in the bi-parental populations, the RDP1, and the AfricaRice breeding lines, we compared the location of the extensive QTL regions 1, 2, and 3 identified in the two bi-parental populations with the better resolved GWA-QTLs identified in the RDP1 (Figure 3).

Region 1 (chromosome 1) harbored two, well-defined GWA-QTLs associated with GY-loss (Figure 3). One was identified in *INDICA* in Vallee du Kou (msSNP with pos: 23,302,758 and -log_10_ *p* = 5.21) and the other was identified in *AllPOP* in All HIA stress sites (msSNP with pos: 23,358,388 and -log_10_ *p* = 5.24). We examined haplotype variation in Region 1, an extended ∼110 kb region encompassing the two GWA-QTLs (referred to as Nested Haplotypes, NH, in Figure 4). A set of 10 informative SNPs was identified that distinguished the nested haplotypes and included the two msSNPs (pos: 23,302,758 and 23,358,388) defining the GWA-QTLs. The favorable alleles at these SNPs (A for SNP_23,302,758 and C for SNP_23,358,388) were both carried by NH1, the N-L-19-like nested haplotype (Figure 4), but were rare in the RDP1, found at MAF = 0.09 and MAF = 0.05, respectively (Supplementary Table S4). Based on this evidence, we conclude that NH1 represents the HIA stress tolerant haplotype at Region 1 and that it is carried by the most resistant lines in the bi-parental crosses, and the most resistant *indica* lines in RDP1.

The frequency of NH1 among AfricaRice *indicas* and RDP1 *indicas* (46% and 7%, respectively) mirrors the frequency of the extended N-L-19-like haplotype (EH1) originally identified with bi-parental Region 1 (Figure 4). We also found that four *indica* RDP1 accessions (Ming Hui, Rathuwee, Binulawan and SL 22-613) carried both EH1 and NH1 (Supplementary Table S7). One additional RDP1 *indica* variety, Seratoes Hari, carried the smaller NH1 but not the extended EH1; this variety was previously described as having the best combination of favorable alleles across the GWA-QTL regions G2-G5 (Supplementary Table S5). The IR64-Sub1-like nested haplotype (NH2) carrying both unfavorable alleles at the GWA-QTL msSNPs, was found at the same frequency as EH2 among the *indica* AfricaRice accessions (36% for both) but at a higher frequency among *indica* RDP1 accessions (49% vs 34%) (Figure 4). Neither NH1 nor NH2 were found in *aus* or *O. glaberrima* lines in this study. Similar to what was observed in the extended Region 1, all 21 *O. glaberrima* lines shared a common *O. glaberrima-*specific haplotype (NH4) in the nested GWA region. Eighty percent of *aus* lines carried both an *aus-*specific nested haplotype (NH3) and the *aus*-specific extended haplotype (EH3) in Region 1 (Figure 4). These observations suggest that common genetic determinants segregating in both the bi-parental populations and in the RDP1 and AfricaRice *indica* varieties are responsible for HIA stress tolerance associated with Region 1. In addition, the co-localization of the GWA-QTLs, identified in different sites and using different mapping populations and sources of germplasm, together with the similar frequencies of the tolerant extended and nested haplotypes (EH1 and NH1) among *indica* accessions, strongly supports Region 1 as a robust and conserved source of genes conferring HIA stress tolerance in West Africa.

No GWA-QTLs co-localized with bi-parental Region 2.

One GWA-QTL co-localized with Region 3 on chromosome 3 (Figure 3). This GWA signal was associated with GY identified in *AllPOP* in Valle du Kou (msSNP with pos: 1,278,045 and -log_10_ *p* = 5.18). To determine whether the biparental QTL for GY in Suakoko, which is among the many overlapping QTLs identified in Region 3 (Figure 2), detected the same genetic determinants as the GWA-QTL for GY in Valle du Kou (Figure 3), we examined haplotype variation in the extended region (∼540 kbp) surrounding the GWA-QTL msSNP (Figure 4). The minor allele for the msSNP (MAF = 0.39 in *AllPOP)* at this GY GWA-QTL is widely distributed across all RDP1 subpopulations but was not segregating in the bi-parental crosses. In the RDP1, it is fixed in *indica* and almost fixed in *aus* (Supplementary Figure S42), which explains why the GY QTL was mapped only in *AllPOP*, and not in the *INDICA* varietal group. The haplotypes observed in the N-L-19 and IR64-Sub1 parents were found at frequencies between 10-20% among *indica* accessions of the RDP1 (EH1 = 18%; EH2 = 11%) (Figure 4). Using EH1 (NL-L-19) and EH2 (IR64-Sub1) to define genotypic groups within RDP1 *indica* accessions, we compared GY for the two groups observed in Valle du Kou and found no significant difference (*t*-test, *p* > 0.05). This suggests that the genetic determinants of the GWA-QTL for GY in Valle du Kou and the Region 3 bi-parental QTL for GY in Suakoko are not the same. Thus, unlike in Region 1, we did not uncover evidence of common genetic determinants in the bi-parental populations and in the RDP1 accessions associated with GY under stress in Region 3.

As previously discussed, the majority of bi-parental QTLs detected in Region 3 were associated with flowering time under both control and HIA stress conditions (Figure 2) and the regon co-localized with *DTH3*, a gene involved in flowering induction in both Asian and African rice (Lee et al., 2004; Bian et al., 2011). To determine whether variation within the flowering time gene itself is more predictive of grain yield under stress in Valle du Kou than was the extended haplotype, we undertook a more in-depth examination of *DTH3* variation among the *INDICA* RDP1 accessions. The results of this examination are provided in Supplementary Analysis S1 and dismiss the possibility that variation in the *DTH3* haplotypes among the *INDICA* RDP1 accessions is associated with their GY variation in Valle du Kou.

### Re-evaluation of High Yielding RDP1 Accessions

In 2017WS, a second field trial was undertaken to determine whether the best yielding RDP1 accessions identified based on 2012-2013WS evaluation data would again display superior GY performance. The accessions for this field trial were selected based on their combined GY performance in two HIA stress sites, Suakoko (Liberia) and Vallee du Kou (Burkina Faso), as well as in the control location, Ibadan (Nigeria) during the 2012-2013WS. These criteria were designed to identify promising accessions that showed both high-yield potential (in the control location) and consistently high levels of “yield under stress” and tolerance to HIA stress (in terms of LBS) in two different stress locations in West Africa. We identified nine accessions that met these requirements: six *indica* varieties (Seratoes Hari, Rathuwee, IR 36, Chiem Chanh, Pao-Tou-Hung and JM70), one *admixed-indica* (Tsipala 421) and, surprisingly, two *temperate japonica* varieties (Lusitano and Bahia, from Portugal and Spain, respectively) (Supplementary Figure S50A). Because of their exceptionally high GY performance under HIA stress conditions, four of these lines (JM70, Tsipala 421, Lusitano and Bahia) were considered outliers and had been removed from the GWA analysis. Nonetheless, we were interested to re-evaluate them in the 2017WS trial. We also included two additional *indica* varieties that did not fulfill the yield criteria mentioned above. The first, Ming Hui, is often used as a parent in hybrid rice breeding, and among the RDP1 accessions, performed well under HIA stress conditions but not particularly well under control conditions (Supplementary Figure S50A). The second, Sigadis, was among the fifty top-yielding varieties in Ibadan and showed very high yield in Suakoko but did not germinate in Vallee du Kou (Supplementary Table S1). Eight of the superior RDP1 lines (with the exception of IR36) displayed not only higher yield, but also lower LBS symptoms, compared to the majority of lower yielding RDP1 varieties (Supplementary Figure S50A). This observation is consistent with the negative correlation between LBS and GY present in the RDP1 panel and described above (Figure 1C), and with the hypothesis that lower LBS leaf symptoms are associated with better GY performance in HIA stress sites (Audebert and Fofana, 2009).

The eleven high-yielding accessions from the RDP1 were re-evaluated during the 2017WS in two HIA stress sites, Suakoko and Edozhigi, as well as in Ibadan (control). The 2017 trial also included eight varieties used in the AfricaRice breeding program: IR64-Sub1, N-L-19 and N-L-43 (parents of the two bi-parental populations), WITA 4, FARO 57 and Taichung Native 1 (*indica*), and TOG 7250-A and TOG 14367 (*O. glaberrima*). Of the eleven RDP1 accessions, three *indica* varieties, Pao-Tou-Hung, Chiem Chanh, and Seratoes Hari, and the *temperate japonica* variety Bahia displayed low LBS symptoms and intermediate to high GY under both control and HIA stress conditions again in 2017WS (Supplementary Figure S50B). These four varieties outperformed most of the elite AfricaRice breeding material, with the exception of WITA 4 (*indica*) and TOG 14367 (*O. glaberrima*), both known for their high levels of HIA stress tolerance (Sikirou et al., 2015, 2018). Of the three RDP1 *indica* varieties, Pao-Tou-Hung and Chiem Chanh carry favorable alleles across the high-density GWA-QTL regions G2 and either G3 (Pao-Tou-Hung) or G5 (Chiem Chanh) (Figure 3 and Supplementary Table S5). The third RDP1 *indica* variety, Seratoes Hari carries favorable alleles across all four (G2-G5) high-density GWA-QTL regions (Supplementary Table S5) and also for the tolerant nested haplotype (NH1) in bi-parental Region 1 (Figure 4 and Supplementary Table S7). This highly favorable allelic combination, together with a consistent grain yield performance across years and locations, makes Seratoes Hari the top-ranked RDP1 *indica* variety for inclusion in a recurrent selection program designed to enhance HIA stress tolerance in West African rice.

The presence of a *temperate japonica* variety (Bahia) among the best performing RDP1 lines was surprising, considering that almost all varieties showing tolerance to HIA stress in this study were *indica* varieties. While Bahia may not be directly useful to the AfricaRice breeding program, it stands out as an interesting source of tolerance to Fe-associated stress. Finally, despite the fact that we did not identify specific QTLs associated with favorable alleles coming from *O. glaberrima* in the NERICA parents examined here, our work nonetheless confirms the long-standing interest in *O. glaberrima* as a potential source of HIA stress tolerance (Linares, 2002), given the fact that varieties such as TOG 14367 consistently rank among the best performing lines in HIA stress environments in West Africa (Sikirou et al., 2018).

### Inventory of HIA Stress Tolerance QTLs Reported in the Literature

The genetic bases of HIA stress tolerance in rice have been investigated by the scientific community for more than 20 years. Here we report an inventory of QTLs mapped in 13 independent publications (from 1997 to 2020) which used different rice mapping populations/diversity panels exposed to HIA stress conditions (Wu et al., 1997, 1998, 2014; Wan et al., 2003; Ouyang et al., 2007; Dufey et al., 2009, 2012, 2015; Shimizu, 2009; Fukuda et al., 2012; Matthus et al., 2015; Zhang et al., 2017; Diop et al., 2020).

A total of 212 QTLs were identified using a set of morpho-physiological, phenology and yield component traits (Supplementary Table S8). The large number and genome-wide distribution of QTLs highlights the complexity of HIA stress tolerance in rice, a trait influenced by many genetic loci and multiple mechanisms (Becker and Asch, 2005; Engel et al., 2012; Sikirou et al., 2015; Mahender et al., 2019; Diop et al., 2020). Supplementary Figure S51 shows the distribution of the 212 QTLs on the twelve chromosomes of the rice genome. A majority of these QTLs (84%) were detected in controlled experiments (orange in Supplementary Figure S51) while only 34 QTLs (16%) were detected in field experiments (red in Supplementary Figure S51). QTLs for HIA stress tolerance identified in field experiments are considered more relevant for breeding applications than those detected under controlled conditions (mainly hydroponics in growth chamber or greenhouse). This is mainly due to the simplified forms of stress imposed under controlled environments compared with field situations where large, discontinuous concentrations of reduced iron (Fe^2+^) interact with multiple other ions including P, K, and Zn, causing numerous nutritional disorders due to both deficiencies and toxicities (Becker and Asch, 2005; Fageria et al., 2008). Nevertheless, a closer look into QTLs detected in more “simplified” controlled experiments, where it is possible to perform more accurate phenotyping due to lower environmental noise, might help identify specific genetic determinants of plant response to HIA stress soils, otherwise masked by the complexity of field environments.

In the present study, we identified QTL regions characterized by multiple, overlapping QTLs (Regions 1-3 and G2-G5) associated with field-based HIA stress tolerance. These regions were detected in different populations and in different West African HIA stress hotspot sites. We compared the locations of the high-density regions identified in this study (purple in Supplementary Figure S51) with those reported in the literature to determine whether their detection in independent studies further validates the robustness of our findings. Interestingly, bi-parental Regions 2 and 3 co-localized with many QTLs found in the literature inventory (blue density curve in Supplementary Figure S51).

Region 2, which showed no overlap with GWAS QTLs reported in this study, overlapped with 13 previously reported QTLs on chromosome 1. Ten out of 13 of these QTLs where detected in hydroponics experiments using populations derived from a cross between the *indica* variety IR64 (susceptible) and the *japonica* Azucena (moderately tolerant) for traits such as leaf bronzing, ROS scavenging antioxidant enzyme activity and stomatal conductance (Supplementary Table S8). These observations further validate our findings for bi-parental Region 2, that comprised one GY loss and two LBS QTLs (Figure 2), and for which the susceptible regional haplotype (EH2) was associated with the IR64-Sub1 parent (Figure 4). The remaining three QTLs overlapping with Region 2 were identified by GWA mapping of RDP1 accessions for LBS in a hydroponics experiment (Matthus et al., 2015). Of these three QTLs, two were detected using *AllPOP* of RDP1 (including *indica* and *japonica* accessions) and one using only *indicas* (Supplementary Table S8). Our haplotype analysis of Region 2 also identified the tolerant haplotype (EH1) of the NERICA parent as of *indica* origin (Figure 4). These observations reinforce our finding that the introgression of tolerant haplotypes (either from *indica* or *japonica* donors) for Region 2 into the susceptible IR64 genetic background can help reduce LBS symptoms and GY loss of this accession under field HIA stress conditions in West Africa.

Region 3 overlapped with 7 QTLs on chromosome 3. Interestingly, four of these QTLs were identified using a population of recombinant inbred lines derived from the IR64 × Azucena cross in a field experiment conducted in Valle du Kou (Dufey et al., 2012), one of the West African hotspot sites considered also in the present study. The four QTLs were mapped for LBS, panicle weight, spikelets per panicle and length of growth cycle (Supplementary Table S8). Region 3 also comprised bi-parental QTLs mapped for LBS, GY, and flowering time (Figure 2). In both Region 2 and Region 3 the parental haplotype of IR64-Sub1 (EH2) was associated with lower GY performance and higher LBS symptoms (Figure 4 and Table 3). The co-location of Region 3 with the QTLs determined in a similar field study, and using an almost identical susceptible donor parent, strongly reinforces the role of Region 3 as an important breeding target for the control of flowering time differences in IR64 which, in turn, improves LBS and GY performance of this genotype under West African field HIA stress conditions.

Unlike Regions 2 and 3, our bi-parental Region 1 (with its GWA nested haplotypes, Figure 4) displayed overlap with only a single QTL in the literature inventory (Supplementary Figure S51). The overlapping QTL was associated with non-photochemical quenching in a hydroponic study (Dufey et al., 2015). Similarly, the high-density GWA QTL regions G2-G5 showed few overlaps with QTLs from the literature inventory (Supplementary Figure S51). This is not entirely surprising considering that the genetic diversity associated with HIA stress tolerance in Region 1 (in Pop1 and Pop2) and with regions G2-G5 (in the RDP1) derives from the *INDICA* (*indica* and *aus*) gene pool based on field evaluation. Only one study in the literature had a similar design. In that study, a set of diverse landraces traditionally grown by farmers in the Casamance region of Senegal was evaluated under HIA stress conditions in the field in southern Senegal, and the authors report that the best-performing lines were predominantly *indica* varieties (Diop et al., 2020). A few QTLs were detected, and though none overlapped with those reported here, it would be interesting to determine whether any of the top-performing Senegalese varieties carried the favorable *indica* QTL haplotypes identified in this study.

## Conclusion

Our observations about the genetic architecture underlying rice response to HIA stress are consistent with the conclusions of Zhang et al. (2017), Diop et al. (2020), and Karavolias et al. (2020) who observe that in inbreeding species such as rice, local adaptation to abiotic stress, including Fe- and Al-toxicity, is often characterized by the accumulation of small-effect and/or rare alleles distributed throughout the genome. We also report the discovery that favorable NERICA alleles for least three QTLs are associated with higher tolerance to iron-associated stress are derived from an *indica* ancestor rather than from the expected *O. glaberrima* progenitor. While the exact source of the *indica* ancestor of N-L-19 remains a mystery, our findings suggest that the tolerant alleles likely evolved in response to HIA stress stress conditions in a very different part of the world (most likely Asia). These results have immediate implications for rice breeding programs in West Africa. The abundance of small-effect loci, predominantly from the *indica* subpopulation, contributing to HIA stress tolerance suggests that a recurrent selection program focusing on an expanded *indica* genepool could be used in combination with genomic selection (GS) to increase the efficiency of the breeding process (Begum et al., 2015; Grenier et al., 2015; Cobb et al., 2019; Frouin et al., 2019). The accuracy of GS models can often be improved by differentially weighting SNPs known to be associated with favorable GWA-QTL alleles, such as those reported here (Spindel et al., 2016; Bhandari et al., 2019; Li et al., 2019; Liu et al., 2019; Rice and Lipka, 2019). Thus, our results support the use of recurrent selection guided by GS and the targeting of favorable SNP alleles as an effective strategy for genetic improvement of rice under high HIA stress conditions in the AfricaRice breeding program.

## Data Availability Statement

The Illumina NGS data for the AfricaRice breeding lines are accessible in the Sequence Read Archive (SRA) at NCBI as Accession Number PRJNA657887 under the Project Title “Cornell University Sequencing of 32 AfricaRice breeding lines” at https://www.ncbi.nlm.nih.gov/sra/PRJNA657887. The 700K SNP High Density Rice Array (HDRA) genotyping dataset for the RDP1 is available at the McCouch lab web-site (http://ricediversity.org/data).

## Author Contributions

AS and VR developed bi-parental populations, and selected and purified lines for sequencing. MS, AS, VS, KK, AM, SN, IA, and VR phenotyped the RDP1 and bi-parental populations in West Africa. GM, JA, VG, YS, FA-P, and SM performed sequencing and haplotype analysis, data curation, and genotypic database development. GM, MS, JA, VS, AG, and VR performed statistical analysis. VR, GA, and SM conceptualized and designed the study. GM, MS, and SM wrote the manuscript. All authors contributed to the article and approved the submitted version.

## Conflict of Interest

The authors declare that the research was conducted in the absence of any commercial or financial relationships that could be construed as a potential conflict of interest.

## Footnotes

1 www.illumina.com

2 http://www.diversityarrays.com/

3 http://ricediversity.org/data

4 https://rapdb.dna.affrc.go.jp/viewer/gbrowse_details/irgsp1?name=Os03g0122600;feature_id=38703

5 http://rapdb.dna.affrc.go.jp/

6 http://archive.gramene.org/markers/

7 http://ensembl.gramene.org/Tools/Blast

8 http://www.ricediversity.org/data/sets/44kgwas/RiceDiversity.44K.MSU6.SNP_Information.MSU7.txt

9 http://ricediversity.org/data

## Supplementary Material

Supplementary Figure 1 Chromosomal distribution per chromosome of the N-L-19 × IR64-Sub1 and N-L-43 × IR64-Sub1 SNP maps.

Supplementary Figure 2 Boxplots and distribution density (residuals) of traits scored for the N-L-19 × IR64-Sub1 population during the 2016 wet season.

Supplementary Figure 3 Boxplots and distribution density (residuals) of traits scored for the N-L-43 × IR64-Sub1 population during the 2017 wet season.

Supplementary Figure 4 Allele frequencies per chromosome of the 1,767 SNP markers identified for the N-L-19 × IR64-Sub1 population.

Supplementary Figure 5 Allele frequencies per chromosome of the 1,897 SNP markers identified for the N-L-43 × IR64-Sub1 population.

Supplementary Figure 6 Boxplots (untransformed data) and distribution density (residuals of Box-Cox transformed data) of the traits scored for the *INDICA*, *AllPOP* and *JAPONICA* groups of the RDP1 accessions in the different West African sites.

Supplementary Figure 7 Boxplots (untransformed data) and distribution density (residuals of Box-Cox transformed data) of the traits scored for the *INDICA*, *AllPOP* and *JAPONICA* groups of the RDP1 accessions in the different West African sites.

Supplementary Figure 8 Boxplots (untransformed data) and distribution density (residuals of Box-Cox transformed data) of the traits scored for the *INDICA*, *AllPOP* and *JAPONICA* groups of the RDP1 accessions in the different West African sites.

Supplementary Figure 9 Boxplots (untransformed data) and distribution density (residuals of Box-Cox transformed data) of the traits scored for the *INDICA*, *AllPOP* and *JAPONICA* groups of the RDP1 accessions in the different West African sites.

Supplementary Figure 10 Boxplots (untransformed data) and distribution density (residuals of Box-Cox transformed data) of the traits scored for the *INDICA*, *AllPOP* and *JAPONICA* groups of the RDP1 accessions in the different West African sites.

Supplementary Figure 11 Principal component analysis (PCA) plots based on SNPs for the *AllPOP*, *INDICA* and *JAPONICA* groups of the RDP1 accessions.

Supplementary Figure 12 Zoom-in of bi-parental high-density QTL Regions 1, 2, and 3 for N-L-19 × IR64-Sub1 and N-L-43 × IR64-Sub1 populations.

Supplementary Figure 13 QTL mapping results of traits scored in Lofa (control) and Suakoko (HIA stress) for the N-L-19 × IR64-Sub1 population during the 2016 wet season.

Supplementary Figure 14 QTL mapping results of traits scored in Lofa (control) and Suakoko (HIA stress) for the N-L-19 × IR64-Sub1 population during the 2016 wet season.

Supplementary Figure 15 QTL mapping results of traits scored in Lofa (control) and Suakoko (HIA stress) for the N-L-19 × IR64-Sub1 population during the 2016 wet season.

Supplementary Figure 16 QTL mapping results of traits scored in Lofa (control) and Suakoko (HIA stress) for the N-L-19 × IR64-Sub1 population during the 2016 wet season.

Supplementary Figure 17 QTL mapping results of traits scored in Lofa (control) and Suakoko (HIA stress) for the N-L-19 × IR64-Sub1 population during the 2016 wet season.

Supplementary Figure 18 QTL mapping results of traits scored in Lofa (control) and Suakoko (HIA stress) for the N-L-19 × IR64-Sub1 population during the 2016 wet season.

Supplementary Figure 19 QTL mapping results of traits scored in Lofa (control) and Suakoko (HIA stress) for the N-L-19 × IR64-Sub1 population during the 2016 wet season.

Supplementary Figure 20 QTL mapping results of traits scored in Lofa (control) and Suakoko (HIA stress) for the N-L-19 × IR64-Sub1 population during the 2016 wet season.

Supplementary Figure 21 QTL mapping results of traits scored in Lofa (control) and Suakoko (HIA stress) for the N-L-19 × IR64-Sub1 population during the 2016 wet season.

Supplementary Figure 22 QTL mapping results of traits scored in Ibadan (control) and Edozhigi (HIA stress) for the N-L-43 × IR64-Sub1 population during the 2017 wet season.

Supplementary Figure 23 QTL mapping results of traits scored in Ibadan (control) and Edozhigi (HIA stress) for the N-L-43 × IR64-Sub1 population during the 2017 wet season.

Supplementary Figure 24 QTL mapping results of traits scored in Ibadan (control) and Edozhigi (HIA stress) for the N-L-43 × IR64-Sub1 population during the 2017 wet season.

Supplementary Figure 25 QTL mapping results of traits scored in Ibadan (control) and Edozhigi (HIA stress) for the N-L-43 × IR64-Sub1 population during the 2017 wet season.

Supplementary Figure 26 QTL mapping results of traits scored in Ibadan (control) and Edozhigi (HIA stress) for the N-L-43 × IR64-Sub1 population during the 2017 wet season.

Supplementary Figure 27 QTL mapping results of traits scored in Ibadan (control) and Edozhigi (HIA stress) for the N-L-43 × IR64-Sub1 population during the 2017 wet season.

Supplementary Figure 28 QTL mapping results of traits scored in Ibadan (control) and Edozhigi (HIA stress) for the N-L-43 × IR64-Sub1 population during the 2017 wet season.

Supplementary Figure 29 Manhattan plots and Quantile-Quantile plots of the GWA mapping results for the traits scored in the different West African sites during the 2012-2013 wet seasons in the *AllPOP*, *INDICA* and *JAPONICA* groups of the RDP1 panel.

Supplementary Figure 30 Manhattan plots and Quantile-Quantile plots of the GWA mapping results for the traits scored in the different West African sites during the 2012-2013 wet seasons in the *AllPOP*, *INDICA* and *JAPONICA* groups of the RDP1 panel.

Supplementary Figure 31 Manhattan plots and Quantile-Quantile plots of the GWA mapping results for the traits scored in the different West African sites during the 2012-2013 wet seasons in the *AllPOP*, *INDICA* and *JAPONICA* groups of the RDP1 panel.

Supplementary Figure 32 Manhattan plots and Quantile-Quantile plots of the GWA mapping results for the traits scored in the different West African sites during the 2012-2013 wet seasons in the *AllPOP*, *INDICA* and *JAPONICA* groups of the RDP1 panel.

Supplementary Figure 33 Manhattan plots and Quantile-Quantile plots of the GWA mapping results for the traits scored in the different West African sites during the 2012-2013 wet seasons in the *AllPOP*, *INDICA* and *JAPONICA* groups of the RDP1 panel.

Supplementary Figure 34 Manhattan plots and Quantile-Quantile plots of the GWA mapping results for the traits scored in the different West African sites during the 2012-2013 wet seasons in the *AllPOP*, *INDICA* and *JAPONICA* groups of the RDP1 panel.

Supplementary Figure 35 Manhattan plots and Quantile-Quantile plots of the GWA mapping results for the traits scored in the different West African sites during the 2012-2013 wet seasons in the *AllPOP*, *INDICA* and *JAPONICA* groups of the RDP1 panel.

Supplementary Figure 36 Manhattan plots and Quantile-Quantile plots of the GWA mapping results for the traits scored in the different West African sites during the 2012-2013 wet seasons in the *AllPOP*, *INDICA* and *JAPONICA* groups of the RDP1 panel.

Supplementary Figure 37 Manhattan plots and Quantile-Quantile plots of the GWA mapping results for the traits scored in the different West African sites during the 2012-2013 wet seasons in the *AllPOP*, *INDICA* and *JAPONICA* groups of the RDP1 panel.

Supplementary Figure 38 Manhattan plots and Quantile-Quantile plots of the GWA mapping results for the traits scored in the different West African sites during the 2012-2013 wet seasons in the *AllPOP*, *INDICA* and *JAPONICA* groups of the RDP1 panel.

Supplementary Figure 39 Manhattan plots and Quantile-Quantile plots of the GWA mapping results for the traits scored in the different West African sites during the 2012-2013 wet seasons in the *AllPOP*, *INDICA* and *JAPONICA* groups of the RDP1 panel.

Supplementary Figure 40 Frequencies (%) of favorable and deleterious alleles for the most significant SNPs of the QTLs identified in *AllPOP*, *INDICA* and *JAPONICA* groups of the RDP1 panel.

Supplementary Figure 41 Frequencies (%) of favorable and deleterious alleles for the most significant SNPs of the QTLs identified in *AllPOP*, *INDICA* and *JAPONICA* groups of the RDP1 panel.

Supplementary Figure 42 Frequencies (%) of favorable and deleterious alleles for the most significant SNPs of the QTLs identified in *AllPOP*, *INDICA* and *JAPONICA* groups of the RDP1 panel.

Supplementary Figure 43 Frequencies (%) of favorable and deleterious alleles for the most significant SNPs of the QTLs identified in *AllPOP*, *INDICA* and *JAPONICA* groups of the RDP1 panel.

Supplementary Figure 44 Frequencies (%) of favorable and deleterious alleles for the most significant SNPs of the QTLs identified in *AllPOP*, *INDICA* and *JAPONICA* groups of the RDP1 panel.

Supplementary Figure 45 Frequencies (%) of favorable and deleterious alleles for the most significant SNPs of the QTLs identified in *AllPOP*, *INDICA* and *JAPONICA* groups of the RDP1 panel.

Supplementary Figure 46 Frequencies (%) of favorable and deleterious alleles for the most significant SNPs of the QTLs identified in *AllPOP*, *INDICA* and *JAPONICA* groups of the RDP1 panel.

Supplementary Figure 47 Frequencies (%) of favorable and deleterious alleles for the most significant SNPs of the QTLs identified in *AllPOP*, *INDICA* and *JAPONICA* groups of the RDP1 panel.

Supplementary Figure 48 Frequencies (%) of favorable and deleterious alleles for the most significant SNPs of the QTLs identified in *AllPOP*, *INDICA* and *JAPONICA* groups of the RDP1 panel.

Supplementary Figure 49 Frequencies (%) of favorable and deleterious alleles for the most significant SNPs of the QTLs identified in *AllPOP*, *INDICA* and *JAPONICA* groups of the RDP1 panel.

Supplementary Figure 50 Grain yield performance of high yielding RDP1 accessions (based on the 2012-2013 data) during the 2017 wet season in Ibadan (control), Suakoko and Edozhigi (HIA stress).

Supplementary Figure 51 Chromosomal distribution and density of QTLs for HIA stress tolerance in rice reported in the literature during the last 20 years.

Supplementary Table 1 List of the 296 *O. sativa* accessions of the RDP1 panel and phenotypes (adjusted means) used in this study.

Supplementary Table 2 Number of SNPs per chromosome of the N-L-19 × IR64-Sub1 and N-L-43 × IR64-Sub1 SNP maps.

Supplementary Table 3 Summary information about 32 sequenced AfricaRice breeding lines.

Supplementary Table 4 Significant GWA-QTLs identified for the traits scored in RDP1.

Supplementary Table 5 Allelic combinations of the high-density GWA-QTL regions (G2, G3, G4, G5) in the accessions of the RDP1 *INDICA* group.

Supplementary Table 6 Flanking sequences for the 15 SNPs of bi-parental Regions 1, 2 and 3.

Supplementary Table 7 Combination of the extended, nested and gene haplotypes relative to bi-parental Regions 1, 2 and 3 in the accessions of the RDP1 *INDICA* varietal group and in the AfricaRice breeding lines.

Supplementary Table 8 Inventory of QTLs associated with HIA stress tolerance in rice collected from 13 papers published over the last 20 years.

Supplementary Dataset 1 1,767 SNP markers used for mapping the 445 lines of the N-L-19 × IR64-Sub1 cross.

Supplementary Dataset 2 1,897 SNP markers used for mapping the 310 lines of the N-L-43 × IR64-Sub1 cross.

Supplementary Dataset 3 BLUEs of grain yield (GY) and leaf bronzing score 84 days after seeding (LBS84) for selected accessions (species/subpop in brackets) evaluated in Ibadan (control), Suakoko and Edozhigi (HIA stress) during the 2017 wet season (WS).

Supplementary Analysis 1 In-depth examination of *DTH3* variation among the AfricaRice and RDP1 *INDICA* accessions.

## Abbreviations

ADM: admixed
ADM-IND: admixed *Indica*
ADM-JAP: admixed *Japonica*
BLUE: best linear unbiased estimator
DArT: diversity array technology
FLW: days to flowering
GWA: genome wide association
GY: grain yield
HDRA: high density rice array
LBS: leaf bronzing score
MAF: minor allele frequency
NERICA: NEw RICe for Africa
PHT: plant height
PVE: phenotypic variance explained
QTL: quantitative trait locus
RDP: rice diversity panel
RFLP: restriction fragment length polymorphism
SNP: single nucleotide polymorphism.
