# Supplementary material for: Multiple Small-Effect Alleles of Indica Origin Enhance High Iron-Associated Stress Tolerance in Rice Under Field Conditions in West Africa
Source: Front Plant Sci. 2021 Jan 15;11:604938. doi: 10.3389/fpls.2020.604938 (PMC7874229; doi:10.3389/fpls.2020.604938)
Supplement: Supplementary Figure 1 — Chromosomal distribution per chromosome of the N-L-19 × IR64-Sub1 and N-L-43 × IR64-Sub1 SNP maps. [file Data_Sheet_5.pdf]

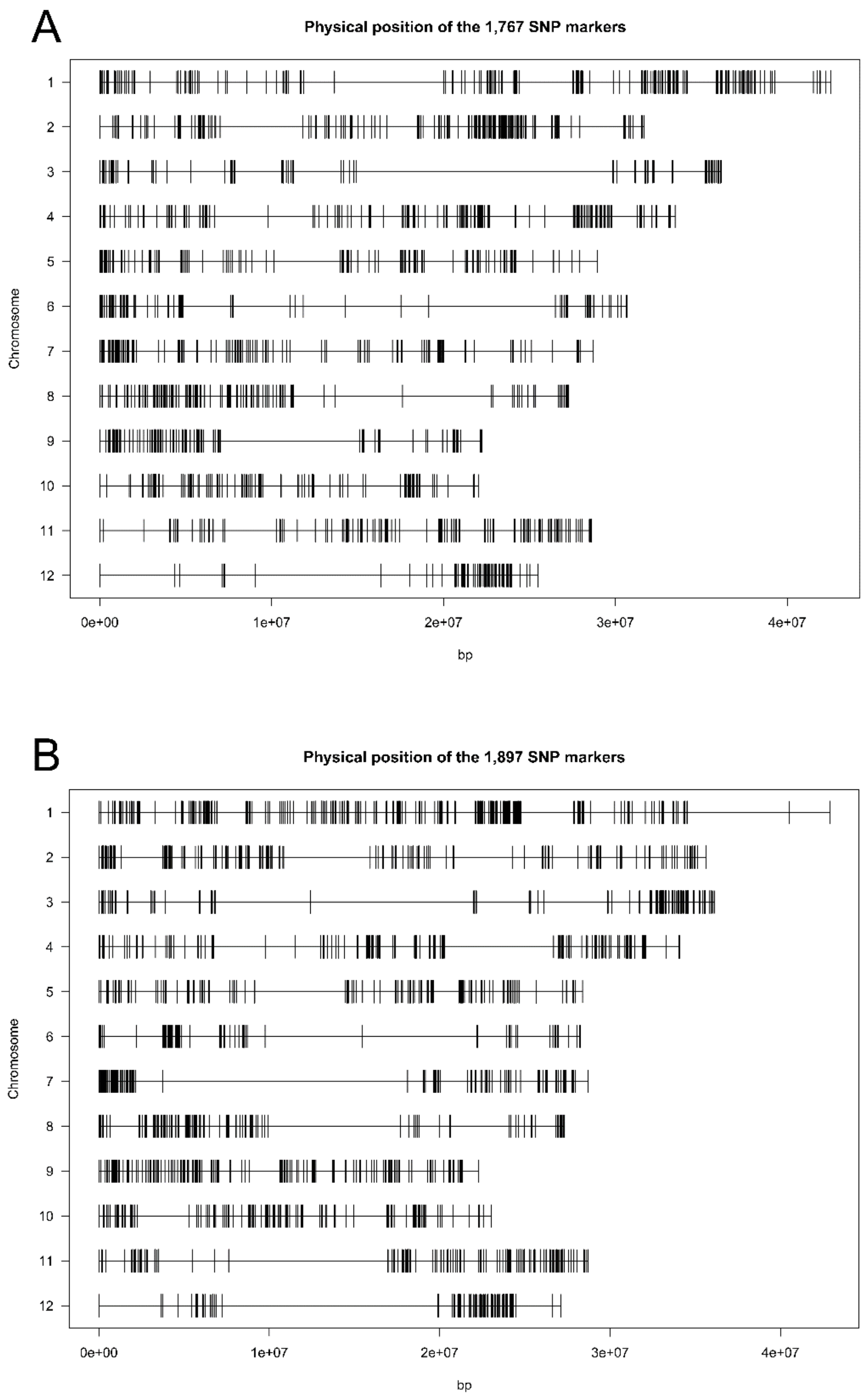

Supplementary Fig. S1. Chromosomal distribution of the 1,767 SNP markers of the F_3-derived F_5 445 lines of the N-L-19 x IR64-Sub1 population (A) and of the 1,897 SNP markers of the F_5 310 lines of the N-L-43 x IR64-Sub1 population (B). SNPs were generated using the DArTseq™ platform and are represented based on their physical position in base pairs (bp).

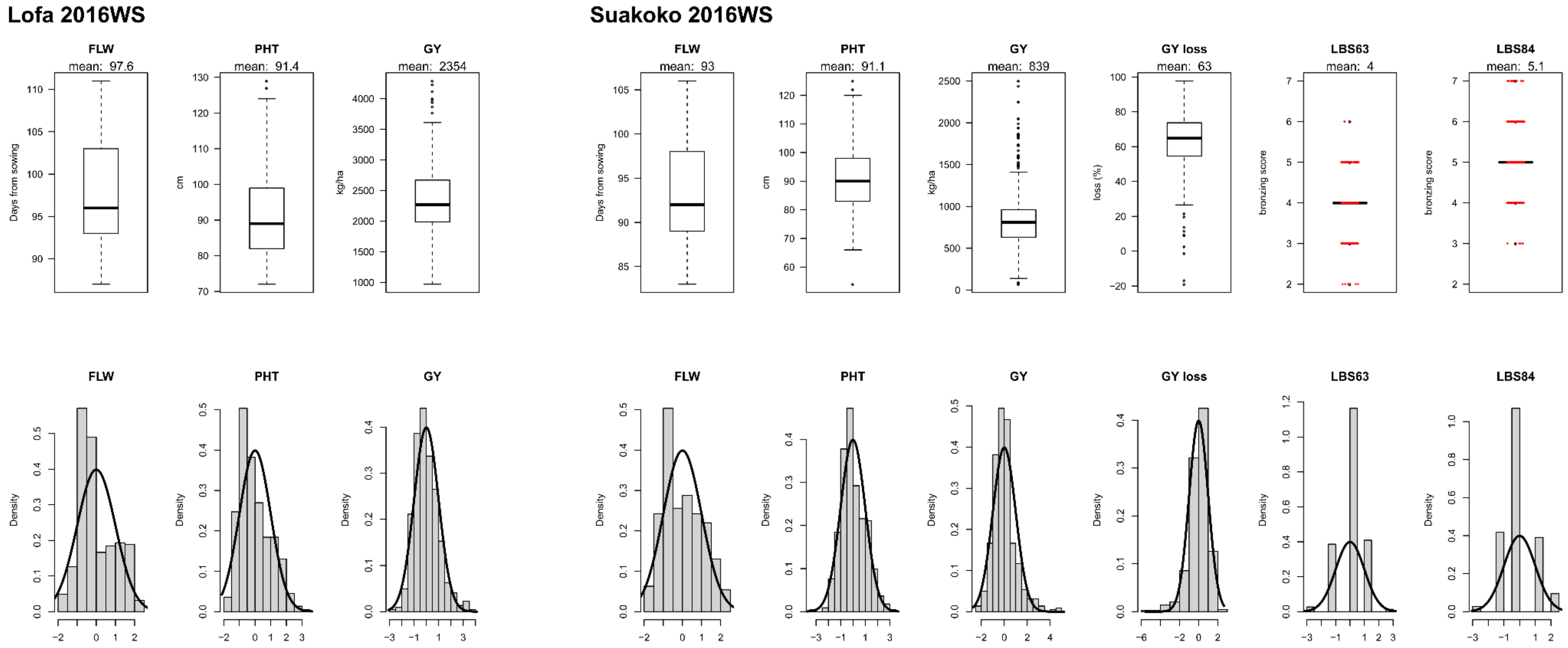

Supplementary Fig. S2. Boxplots and distribution density (residuals) of traits scored for the 445 lines of the N-L-19 x IR64-Sub1 population under control conditions in Lofa (Liberia) and under HIA stress conditions in Suakoko (Liberia), during the 2016 wet season (WS). FLW: days to flowering; PHT: plant height; GY: grain yield; GY-loss: percentage of grain yield loss; LBS63: leaf bronzing score at 63 days after sowing; LBS84: leaf bronzing score at 84 days after sowing. Red overlay on LBS63 and LBS84 represents the number of accessions in each class of this categorical trait.

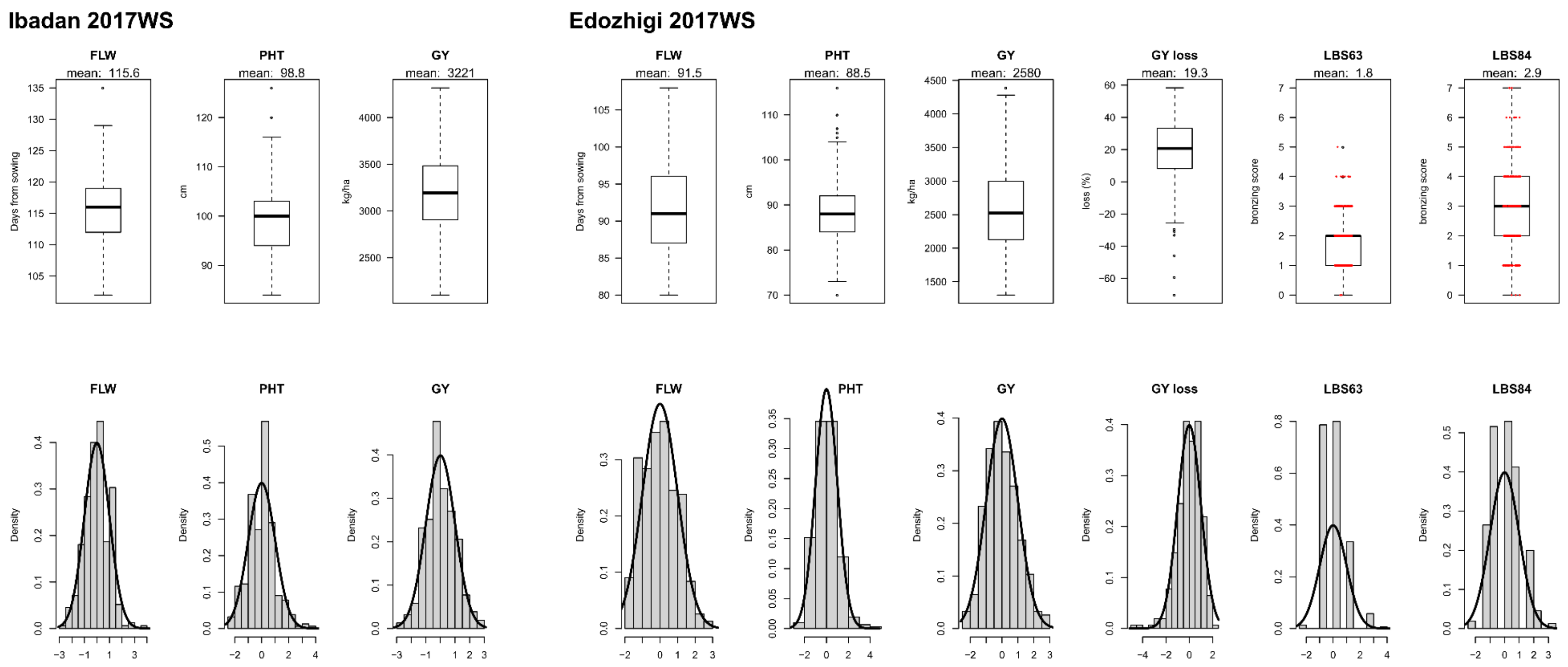

Supplementary Fig. S3. Boxplots and distribution density (residuals) of traits scored for the 310 lines of the N-L-43 x IR64-Sub1 population under control conditions in Ibadan (Nigeria) and under HIA stress conditions in Edozhigi (Nigeria), during the 2017 wet season (WS). FLW: days to flowering; PHT: plant height; GY: grain yield; GY-loss: percentage of grain yield loss; LBS63: leaf bronzing score at 63 days after sowing; LBS84: leaf bronzing score at 84 days after sowing. Red overlay on LBS63 and LBS84 represents the number of accessions in each class of this categorical trait.

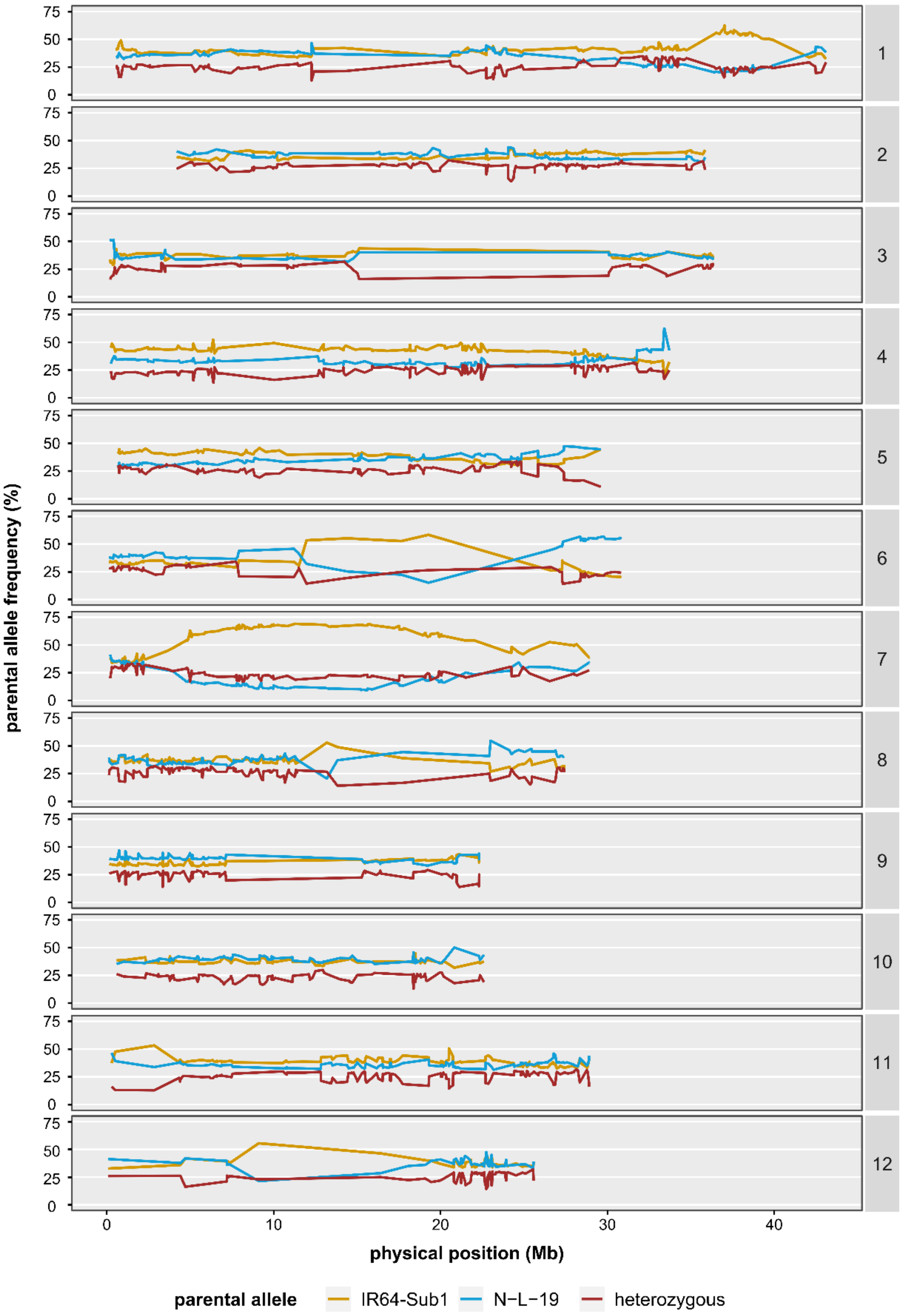

Supplementary Fig. S4. Allele frequencies per chromosome of the 1,767 SNP (DArTseq™) markers identified for the 445 lines of the N-L-19 x IR64-Sub1 population.

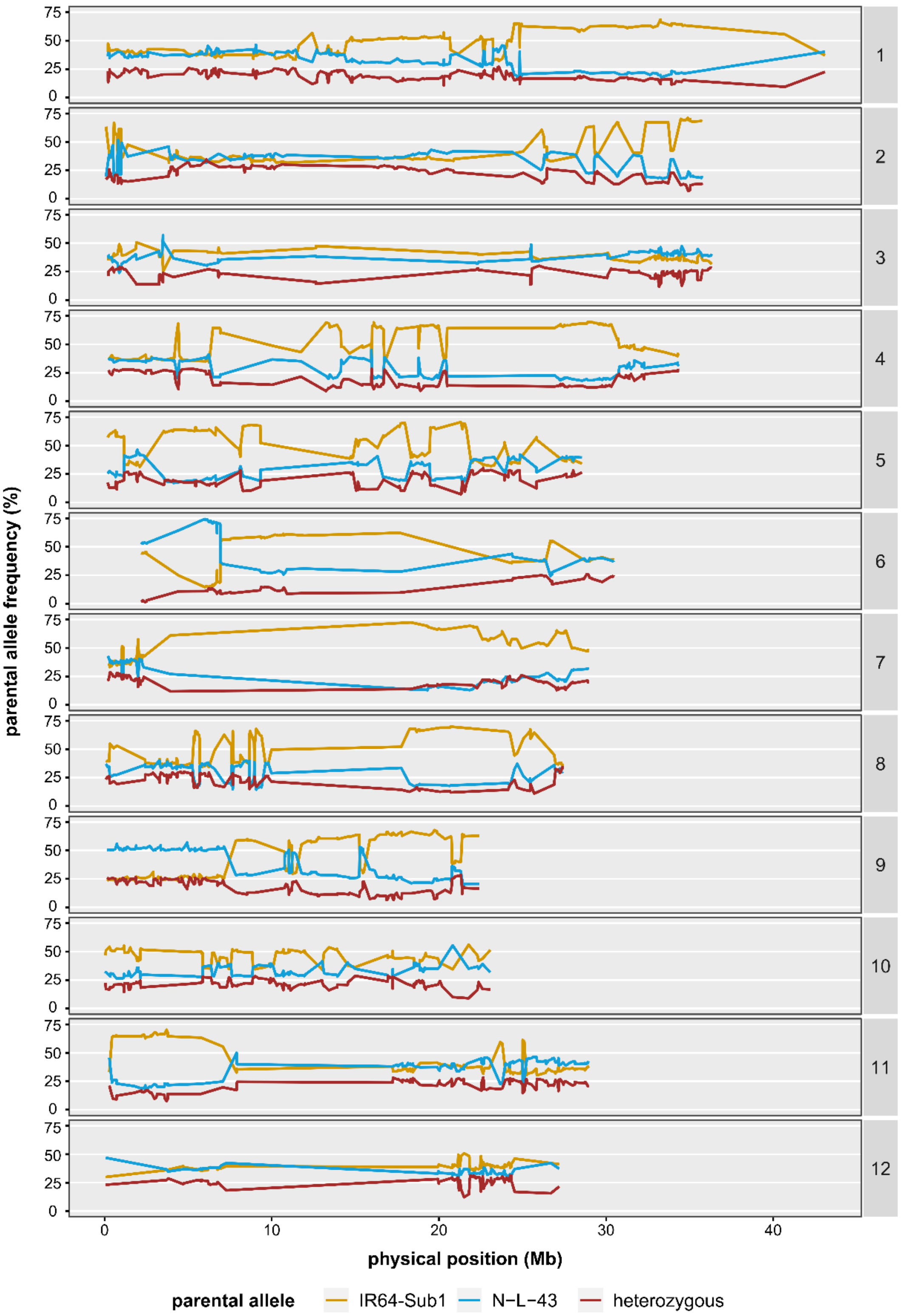

Supplementary Fig. S5. Allele frequencies per chromosome of the 1,897 SNP (DArTseq™) markers identified for the 310 lines of the N-L-43 x IR64-Sub1 population.

Ibadan 12-13WS

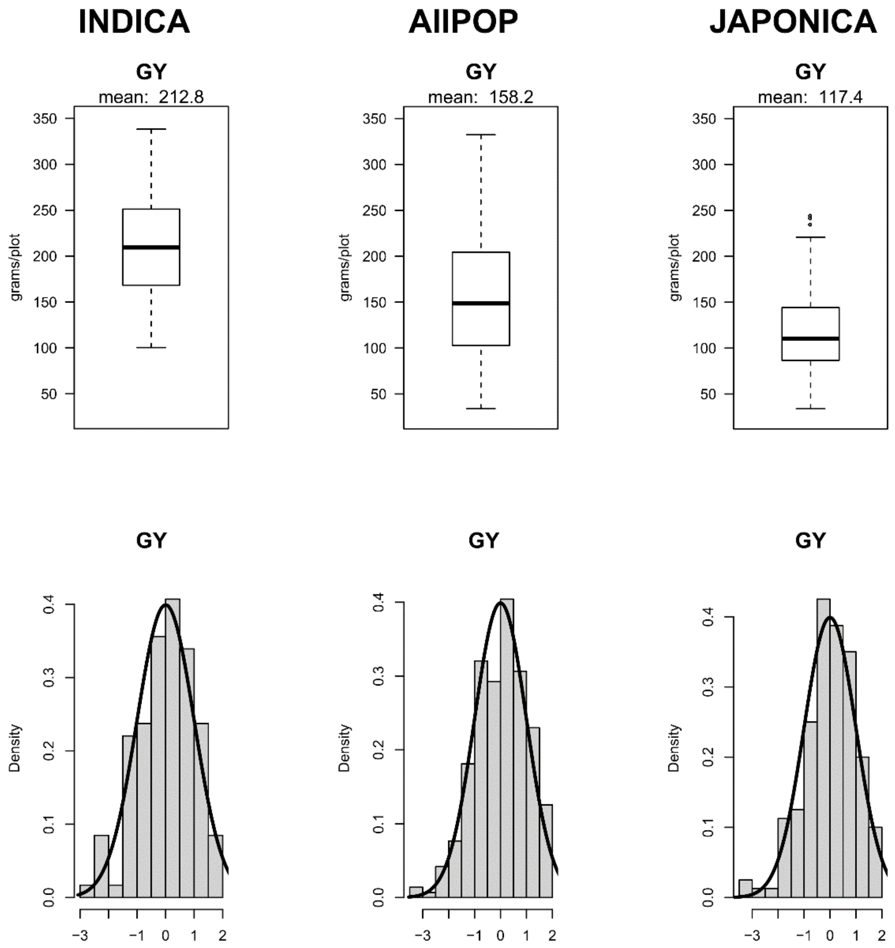

Supplementary Fig. S6. Boxplots (untransformed data) and distribution density (residuals of Box-Cox transformed data) of grain yield (GY) scored for the *INDICA*, *AllPOP* and *JAPONICA* varietal groups of the RDP1 accessions in Ibadan (Nigeria) under optimal conditions during the 2012 and 2013 wet seasons (WS).

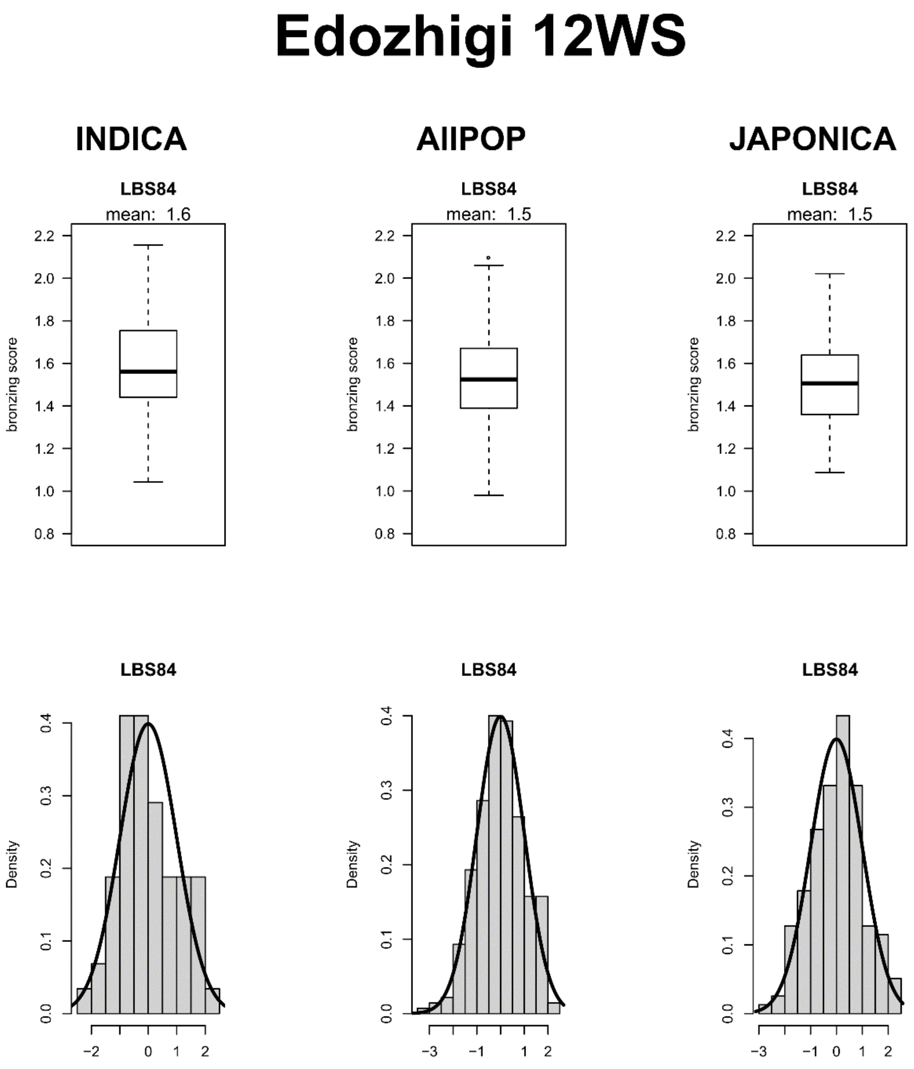

Supplementary Fig. S7. Boxplots (untransformed data) and distribution density (residuals of Box-Cox transformed data) of leaf bronzing (LBS84) scored for the *INDICA*, *AllPOP* and *JAPONICA* varietal groups of the RDP1 accessions in Edozhigi (Nigeria) under HIA stress conditions during the 2012 wet season (WS).

Suakoko 13WS

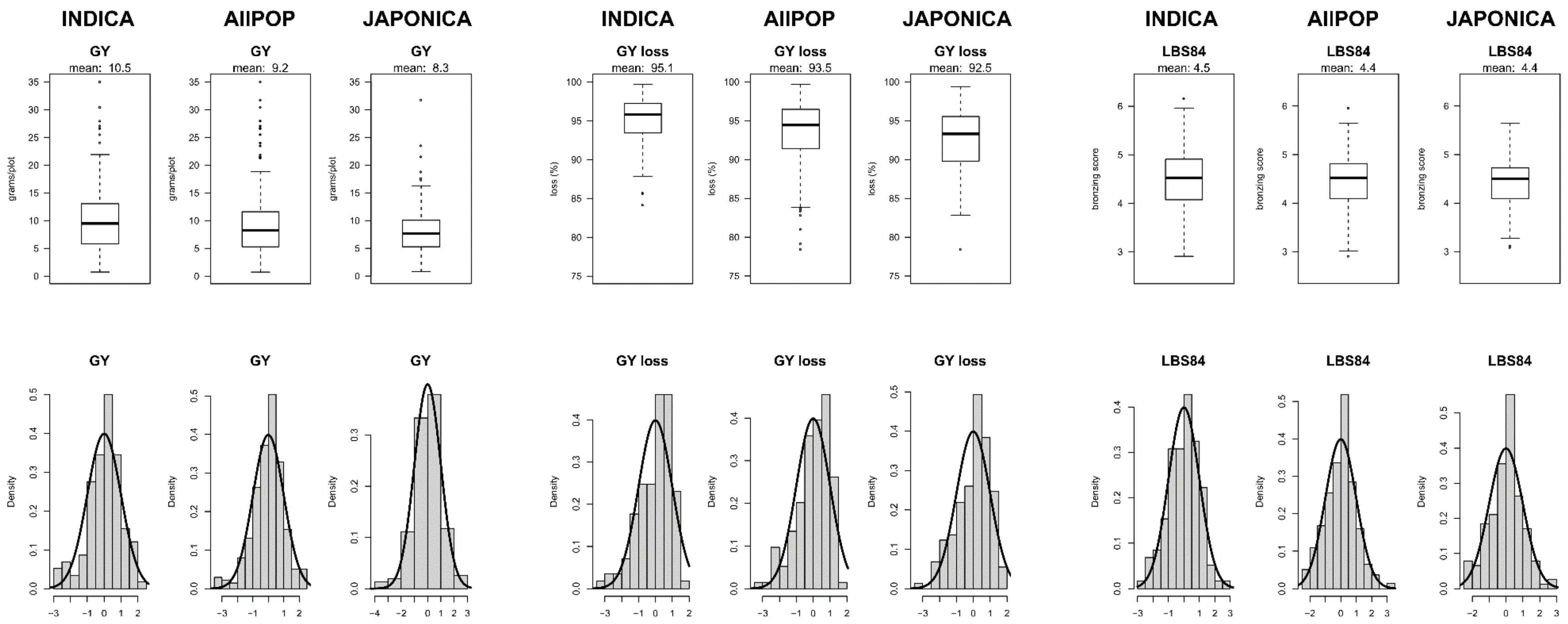

Supplementary Fig. S8. Boxplots (untransformed data) and distribution density (residuals of Box-Cox transformed data) of grain yield (GY), grain yield loss (GY loss) and leaf bronzing (LBS84) scored for the *INDICA*, *AllPOP* and *JAPONICA* varietal groups of the RDP1 accessions in Suakoko (Liberia) under HIA stress conditions during the 2013 wet season (WS).

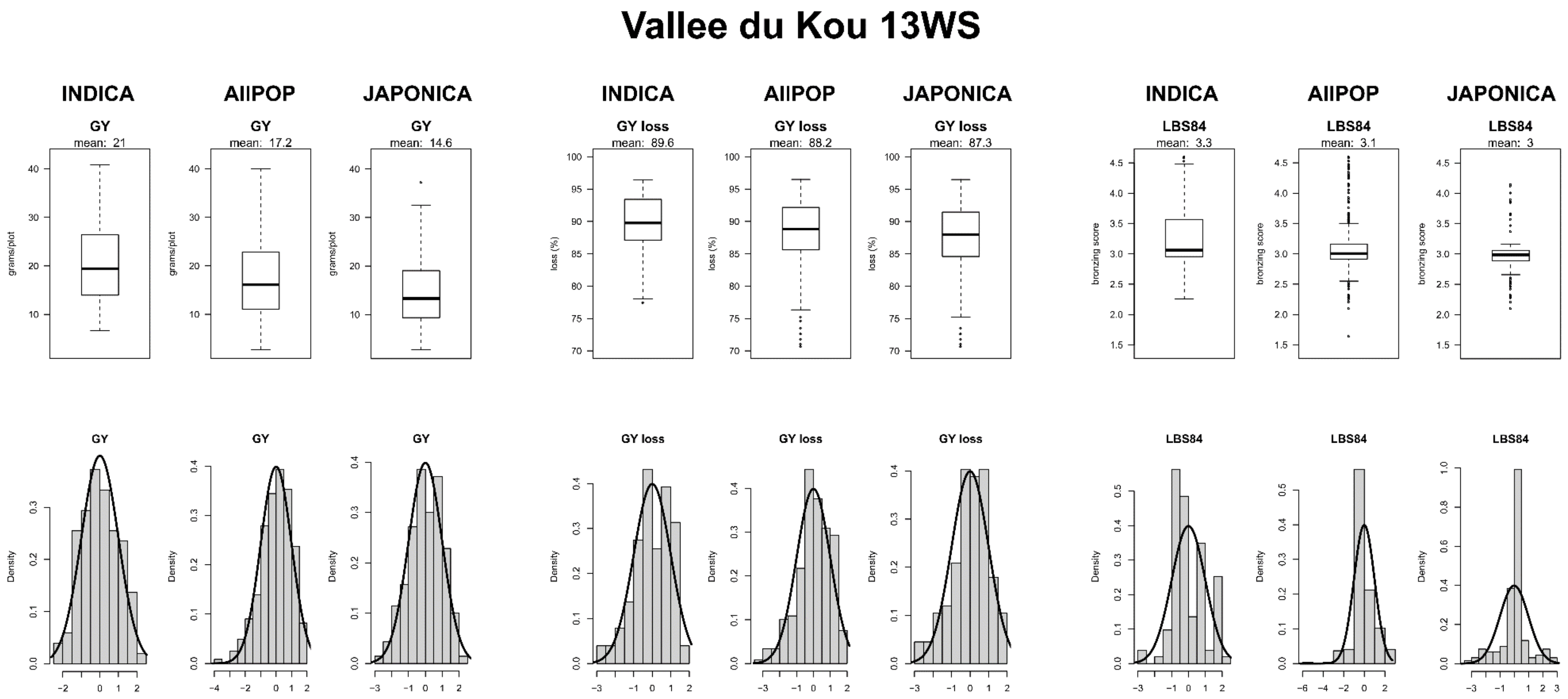

Supplementary Fig. S9. Boxplots (untransformed data) and distribution density (residuals of Box-Cox transformed data) of grain yield (GY), grain yield loss (GY loss) and leaf bronzing (LBS84) scored for the *INDICA*, *AllPOP* and *JAPONICA* varietal groups of the RDP1 accessions in Vallee du Kou (Burkina Faso) under HIA stress conditions during the 2013 wet season (WS).

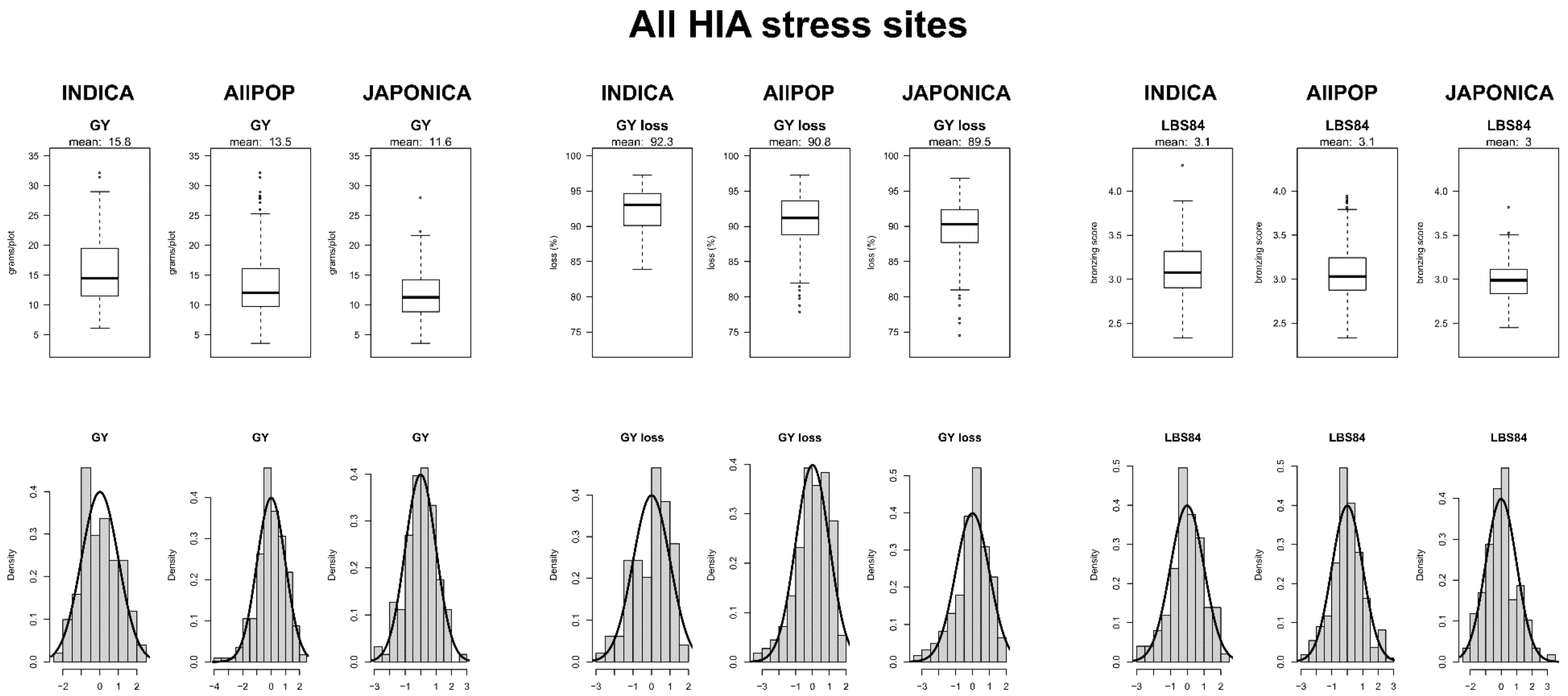

Supplementary Fig. S10. Boxplots (untransformed data) and distribution density (residuals of Box-Cox transformed data) of grain yield (GY), grain yield loss (GY loss) and leaf bronzing (LBS84) scored for the *INDICA*, *AllPOP* and *JAPONICA* varietal groups of the RDP1 accessions in all HIA stress sites (Suakoko and Vallee du Kou for GY and GY loss; Suakoko, Vallee du Kou and Edozhigi for LBS84).

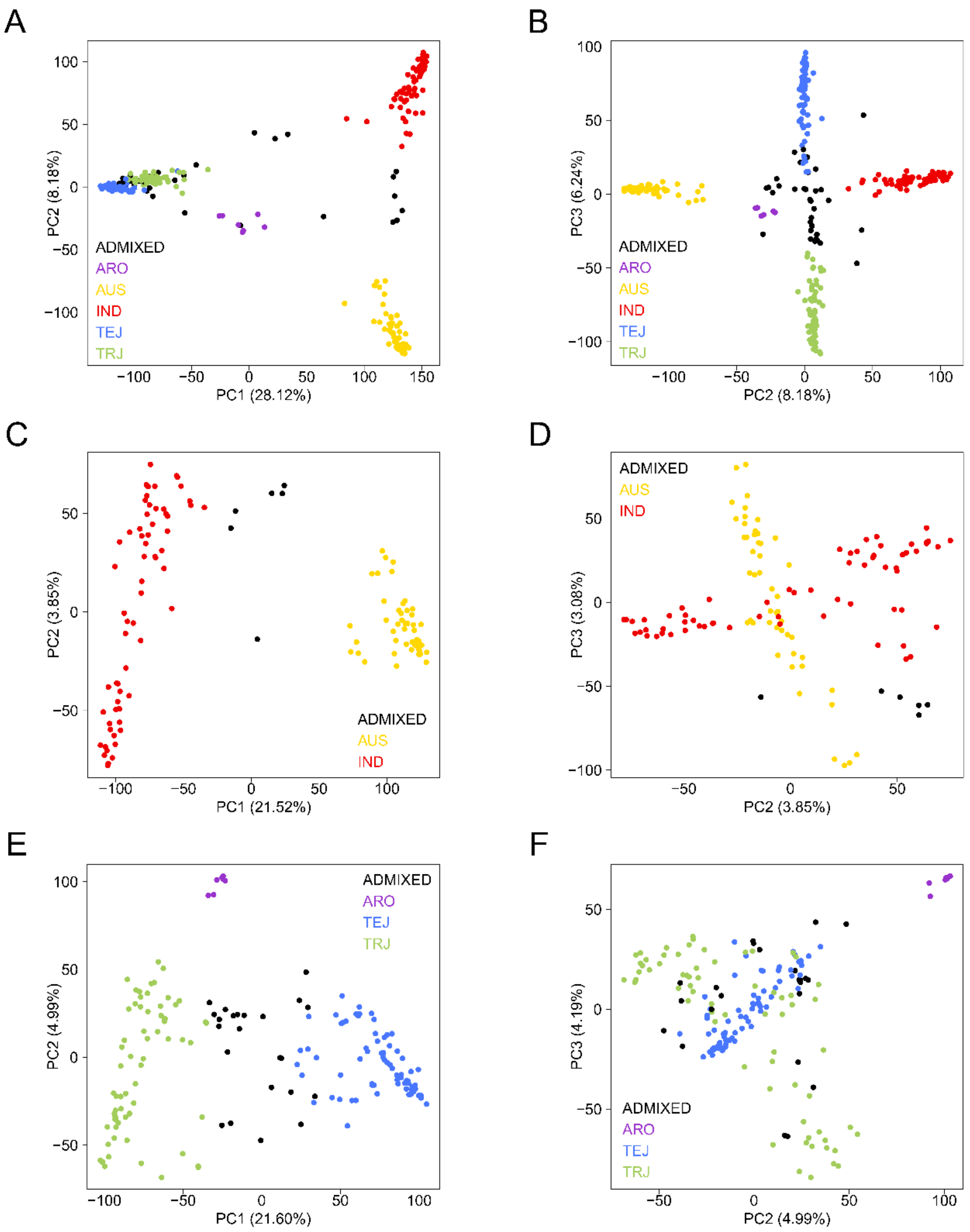

Supplementary Fig. S11. Principal component analysis (PCA) plots, based on 20% randomly selected SNPs for the *AllPOP* (A-B), *INDICA* (C-D) and *JAPONICA* (E-F) varietal groups of the RDP1 accessions. The plots display all possible combinations of the first 3 principal components (PCs). The percentage of variance explained by each PC is displayed in brackets.

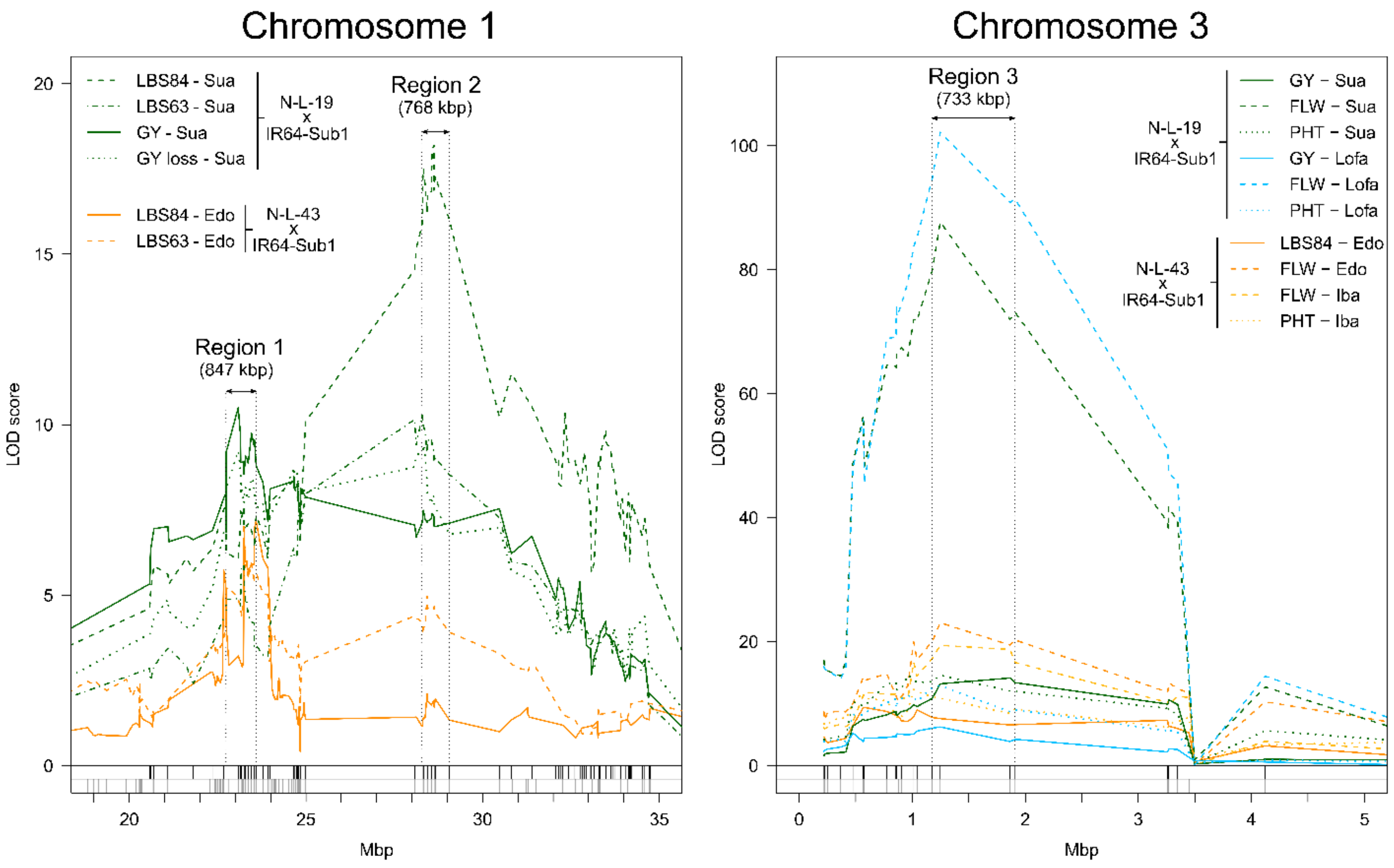

Supplementary Fig. S12. Zoom-in of QTL mapping results of regions showing high density of overlapping QTLs related to HIA-stress response for N-L-19 x IR64-Sub1 and N-L-43 x IR64-Sub1 populations. LOD curve graphs of mapping results for different traits in the three regions (Region 1, 2 and 3) of chromosomes 1 and 3 showing high density of overlapping QTLs. The size of each region (kbp) is reported in brackets. At the bottom of the graphs are displayed the SNP markers used for mapping the two areas (~5Mbp) of chromosome 1 and 3 for N-L-19 x IR64-Sub1 (black markers) and N-L-43 x IR64-Sub1 (gray markers) populations. FLW: days to flowering; PHT: plant height; GY: grain yield; GY loss: grain yield loss; LBS63: leaf bronzing score at 63 days after sowing; LBS84: leaf bronzing score at 84 days after sowing.

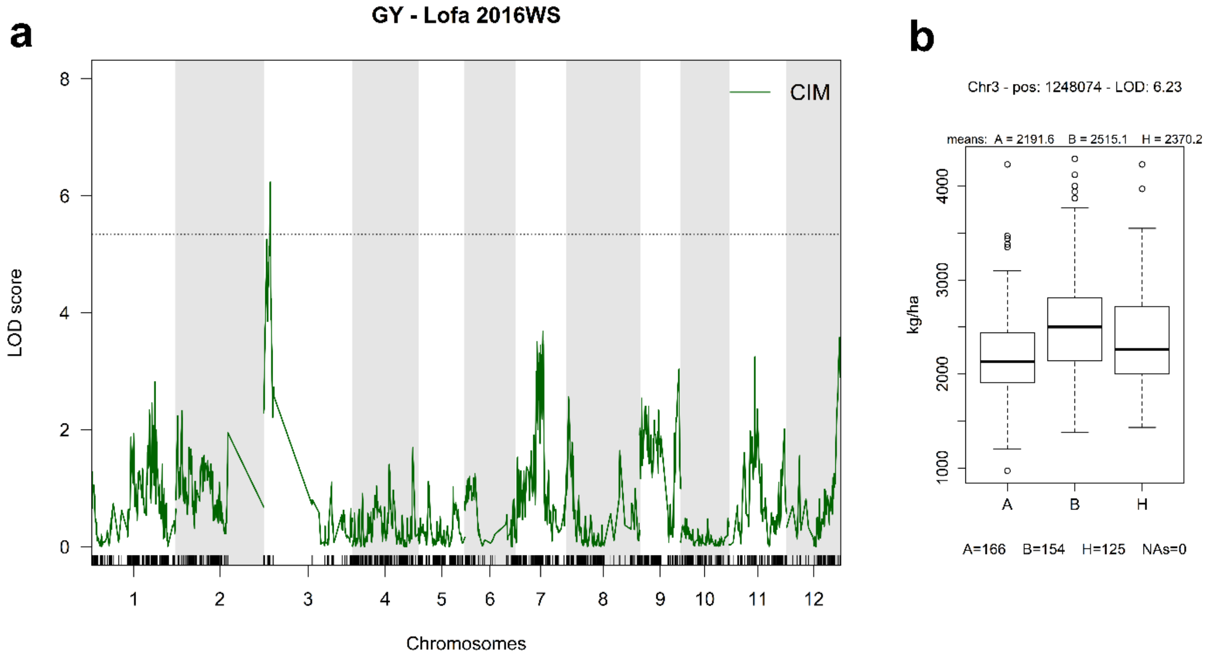

Supplementary Fig. S13. QTL mapping results of GY for the N-L-19 x IR64-Sub1 population in Lofa (control) 2016WS. (a) LOD curve graph (green line) of composite interval mapping (CIM). Black-dotted line: significance LOD threshold (based on 1,000 permutations). (b) Boxplots representing the GY performance of lines carrying IR64-Sub1 (A), NERICA (B) or heterozygous (H) alleles at the locus (SNP-pos) of the most significant (LOD) marker of the chromosome 3 QTL. Number of lines carrying A, B, H and missing (NA) alleles for the marker are reported under the boxplot graph. mean: mean phenotypic value of the A, B and H allelic groups.

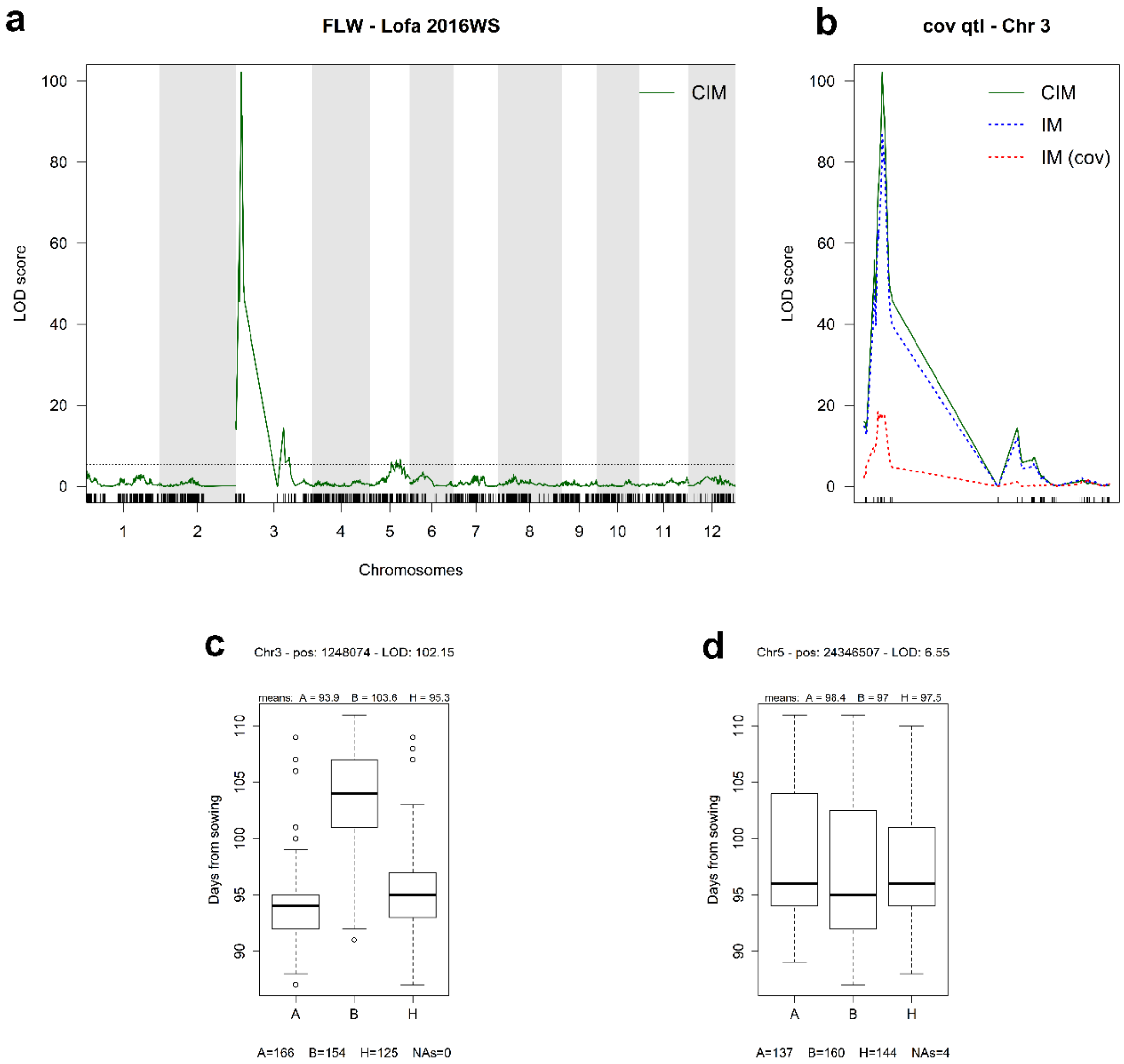

Supplementary Fig. S14. QTL mapping results of FLW for the N-L-19 x IR64-Sub1 population in Lofa (control) 2016WS. (a) LOD curve graph (green line) of composite interval mapping (CIM). Black-dotted line: significance LOD threshold (based on 1,000 permutations). (b) LOD curve graph comparison between CIM (green line), interval mapping (IM, blue line) and interval mapping by fixing the most significant marker (IM cov, red line) for the significant QTL on chromosome 3. (c-d) Boxplots representing the FLW performance of the lines carrying IR64-Sub1 (A), NERICA (B) or heterozygous (H) alleles at the locus (SNP-pos) of the most significant (LOD) marker of the chromosome 3 and 5 QTLs. Number of lines carrying A, B, H and missing (NA) alleles for the marker are reported under each boxplot graph. mean: mean phenotypic value of the A, B and H allelic groups.

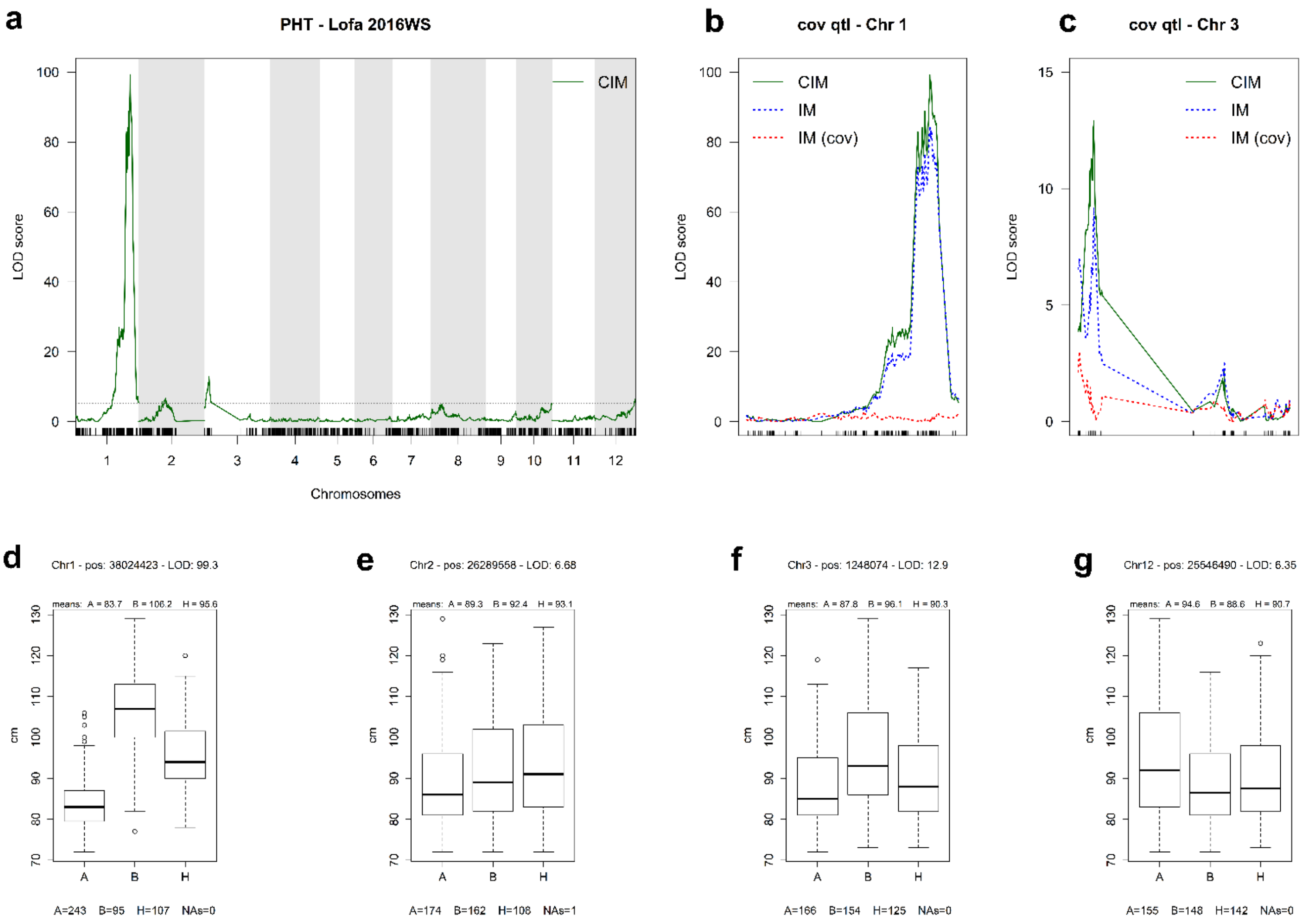

Supplementary Fig. S15. QTL mapping results of PHT for the N-L-19 x IR64-Sub1 population in Lofa (control) 2016WS. (a) LOD curve graph (green line) of composite interval mapping (CIM). Black-dotted line: significance LOD threshold (based on 1,000 permutations). (b-c) LOD curve graph comparison between CIM (green line), interval mapping (IM, blue line) and interval mapping by fixing the most significant marker (IM cov, red line) for the significant QTL on chromosome 1 and 3. (d-g) Boxplots representing the PHT performance of the lines carrying IR64-Sub1 (A), NERICA (B) or heterozygous (H) alleles at the locus (SNP-pos) of the most significant (LOD) marker of the chromosome 1, 2, 3, and 12 QTLs. Number of lines carrying A, B, H and missing (NA) alleles for the marker are reported under each boxplot graph. mean: mean phenotypic value of the A, B and H allelic groups.

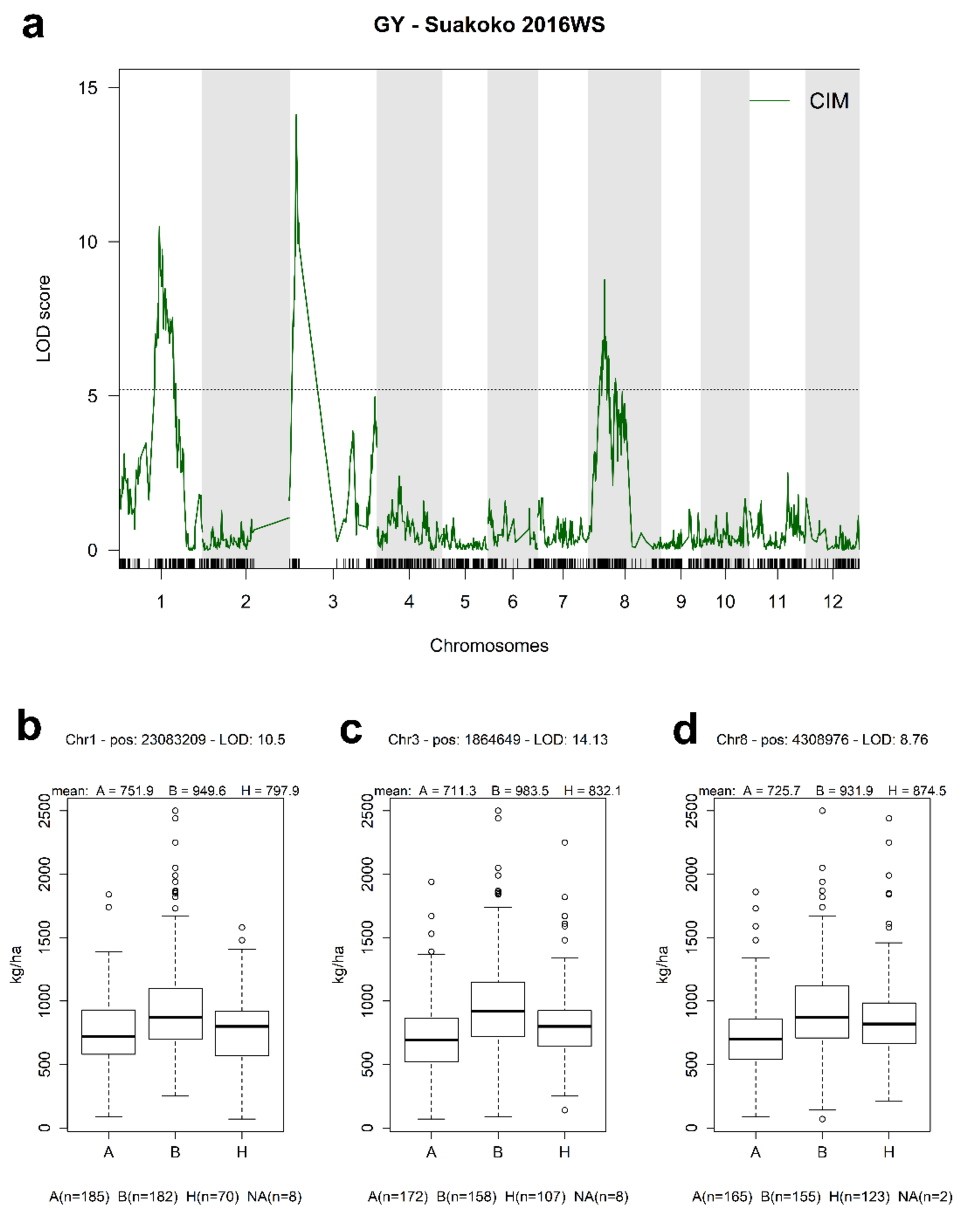

Supplementary Fig. S16. QTL mapping results of GY for the N-L-19 x IR64-Sub1 population in Suakoko (HIA stress site) 2016WS. (a) LOD curve graph (green line) of composite interval mapping (CIM). Black-dotted line: significance LOD threshold (based on 1,000 permutations). (b-d) Boxplots representing the GY performance of the lines carrying IR64-Sub1 (A), NERICA (B) or heterozygous (H) alleles at the locus (SNP-pos) of the most significant (LOD) marker of the chromosome 1, 3, and 8 QTLs. Number of lines carrying A, B, H and missing (NA) alleles for the marker are reported under each boxplot graph. mean: mean phenotypic value of the A, B and H allelic groups.

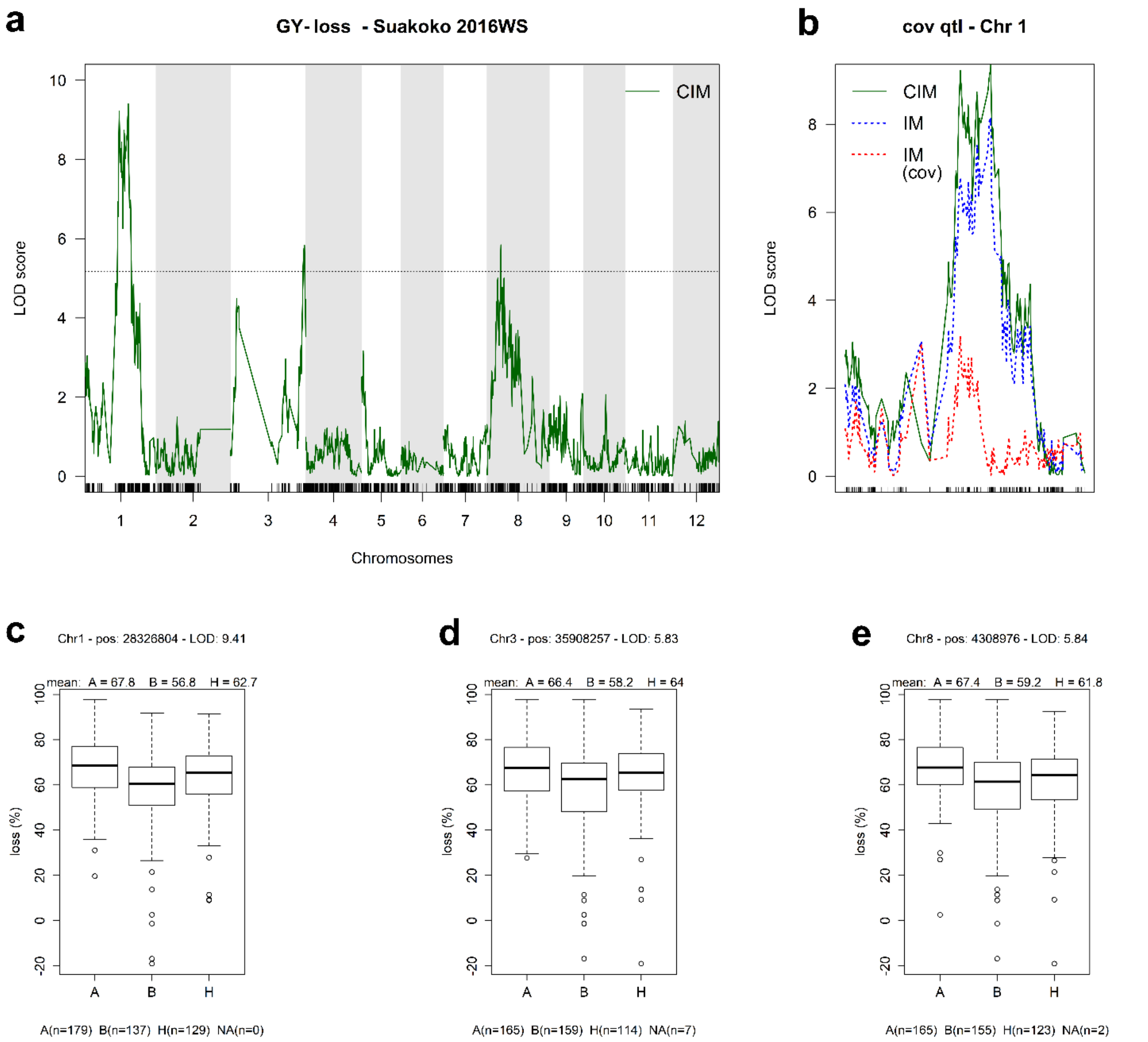

Supplementary Fig. S17. QTL mapping results of GY loss for the N-L-19 x IR64-Sub1 population in Suakoko (HIA stress site) 2016WS. (a) LOD curve graph (green line) of composite interval mapping (CIM). Black-dotted line: significance LOD threshold (based on 1,000 permutations). (b) LOD curve graph comparison between CIM (green line), interval mapping (IM, blue line) and interval mapping by fixing the most significant marker (IM cov, red line) for the significant QTL on chromosome 1. (c-e) Boxplots representing the GY loss performance of the lines carrying IR64-Sub1 (A), NERICA (B) or heterozygous (H) alleles at the locus (SNP-pos) of the most significant (LOD) marker of the chromosome 1, 3, and 8 QTLs. Number of lines carrying A, B, H and missing (NA) alleles for the marker are reported under each boxplot graph. mean: mean phenotypic value of the A, B and H allelic groups.

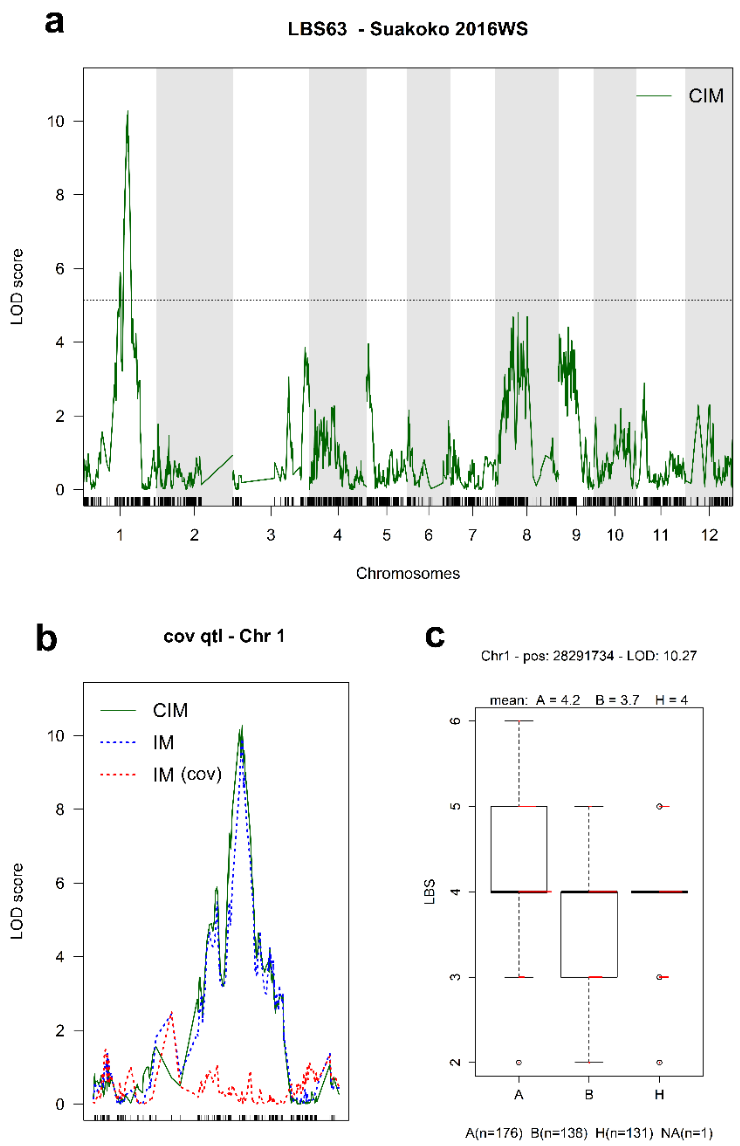

Supplementary Fig. S18. QTL mapping results of LBS63 for the N-L-19 x IR64-Sub1 population in Suakoko (HIA stress site) 2016WS. (a) LOD curve graph (green line) of composite interval mapping (CIM). Black-dotted line: significance LOD threshold (based on 1,000 permutations). (b) LOD curve graph comparison between CIM (green line), interval mapping (IM, blue line) and interval mapping by fixing the most significant marker (IM cov, red line) for the significant QTL on chromosome 1. (c) Boxplots representing the LBS63 performance of the lines carrying IR64-Sub1 (A), NERICA (B) or heterozygous (H) alleles at the locus (SNP-pos) of the most significant (LOD) marker of the chromosome 1 QTL. Red overlay on the boxplots represents the number of accessions in each class of this categorical trait. Number of lines carrying A, B, H and missing (NA) alleles for the marker are reported under the boxplot graph. mean: mean phenotypic value of the A, B and H allelic groups.

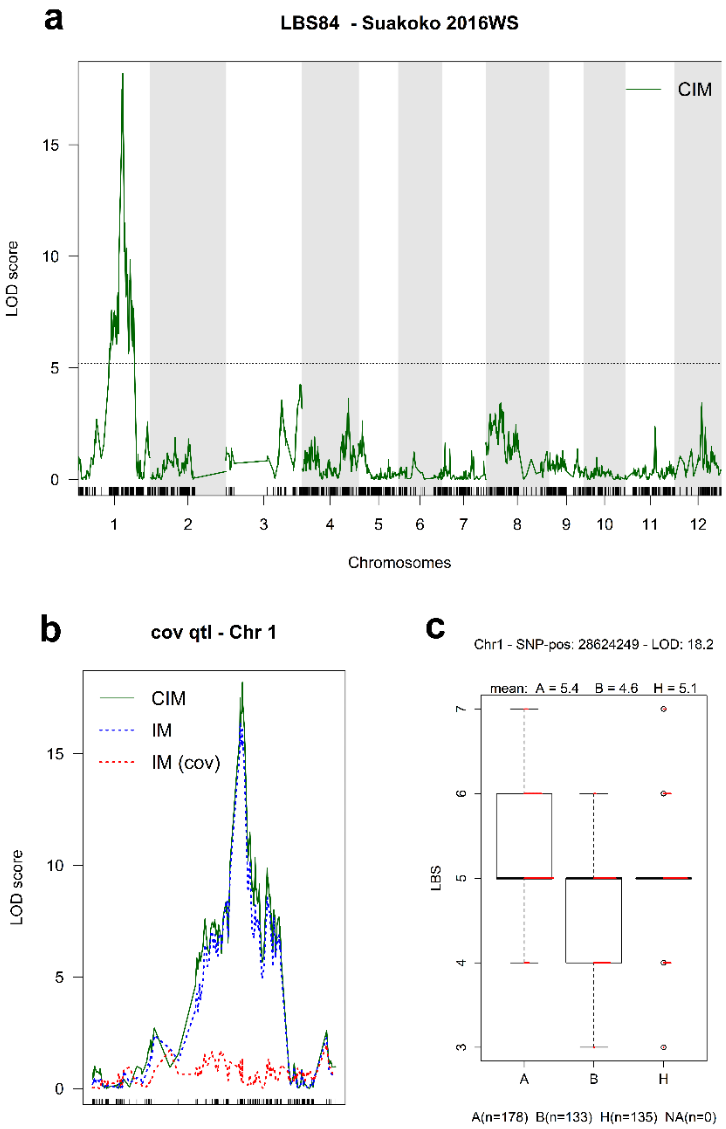

Supplementary Fig. S19. QTL mapping results of LBS84 for the N-L-19 x IR64-Sub1 population in Suakoko (HIA stress site) 2016WS. (a) LOD curve graph (green line) of composite interval mapping (CIM). Black-dotted line: significance LOD threshold (based on 1,000 permutations). (b) LOD curve graph comparison between CIM (green line), interval mapping (IM, blue line) and interval mapping by fixing the most significant marker (IM cov, red line) for the significant QTL on chromosome 1. (c) Boxplots representing the LBS84 performance of the lines carrying IR64-Sub1 (A), NERICA (B) or heterozygous (H) alleles at the locus (SNP-pos) of the most significant (LOD) marker of the chromosome 1 QTL. Red overlay on the boxplots represents the number of accessions in each class of this categorical trait. Number of lines carrying A, B, H and missing (NA) alleles for the marker are reported under the boxplot graph. mean: mean phenotypic value of the A, B and H allelic groups.

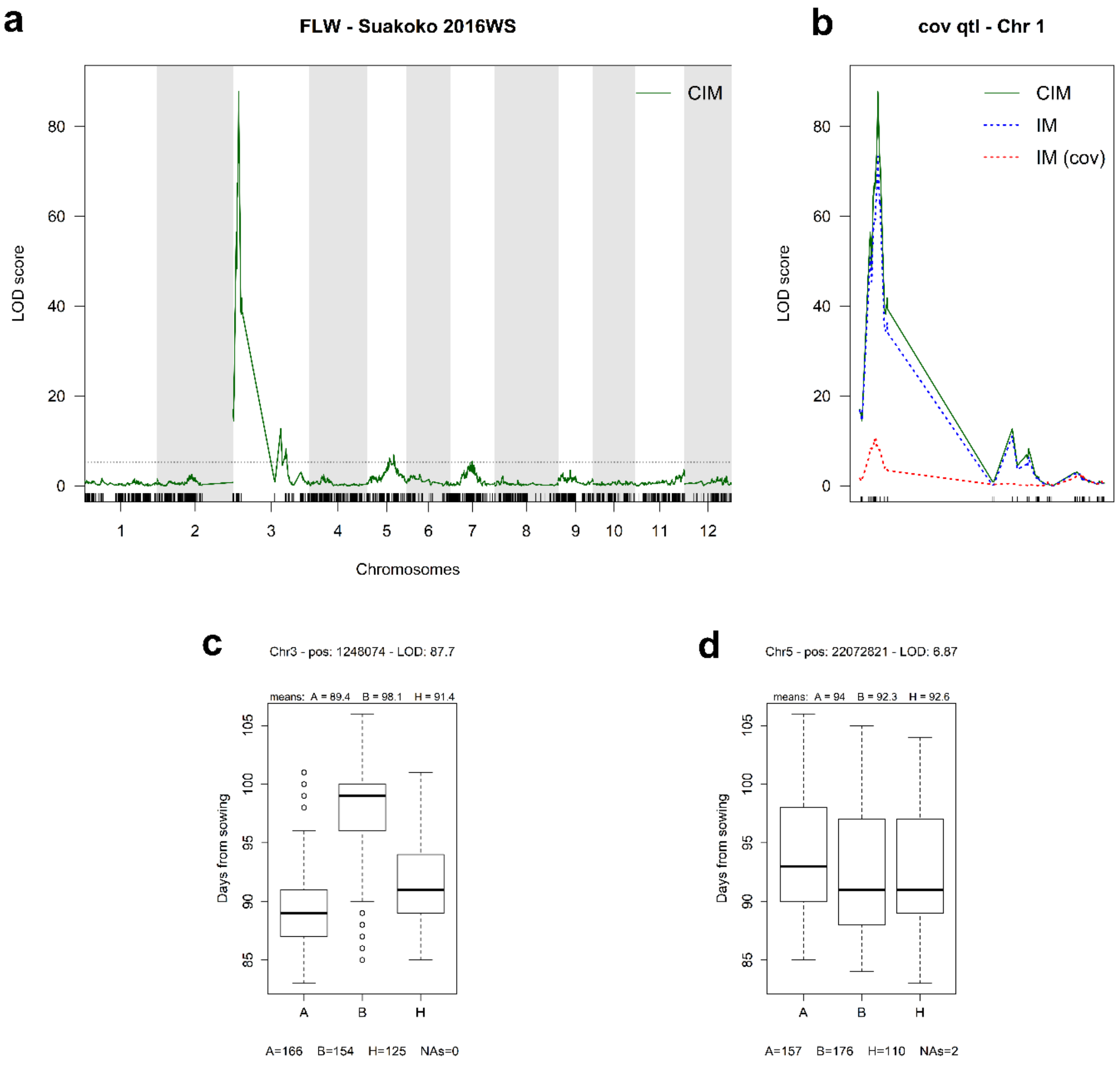

Supplementary Fig. S20. QTL mapping results of FLW for the N-L-19 x IR64-Sub1 population in Suakoko (HIA stress site) 2016WS. (a) LOD curve graph (green line) of composite interval mapping (CIM). Black-dotted line: significance LOD threshold (based on 1,000 permutations). (b) LOD curve graph comparison between CIM (green line), interval mapping (IM, blue line) and interval mapping by fixing the most significant marker (IM cov, red line) for the significant QTL on chromosome 3. (c-d) Boxplots representing the FLW performance of the lines carrying IR64-Sub1 (A), NERICA (B) or heterozygous (H) alleles at the locus (SNP-pos) of the most significant (LOD) marker of the chromosome 3 and 5 QTLs. Number of lines carrying A, B, H and missing (NA) alleles for the marker are reported under each boxplot graph. mean: mean phenotypic value of the A, B and H allelic groups.

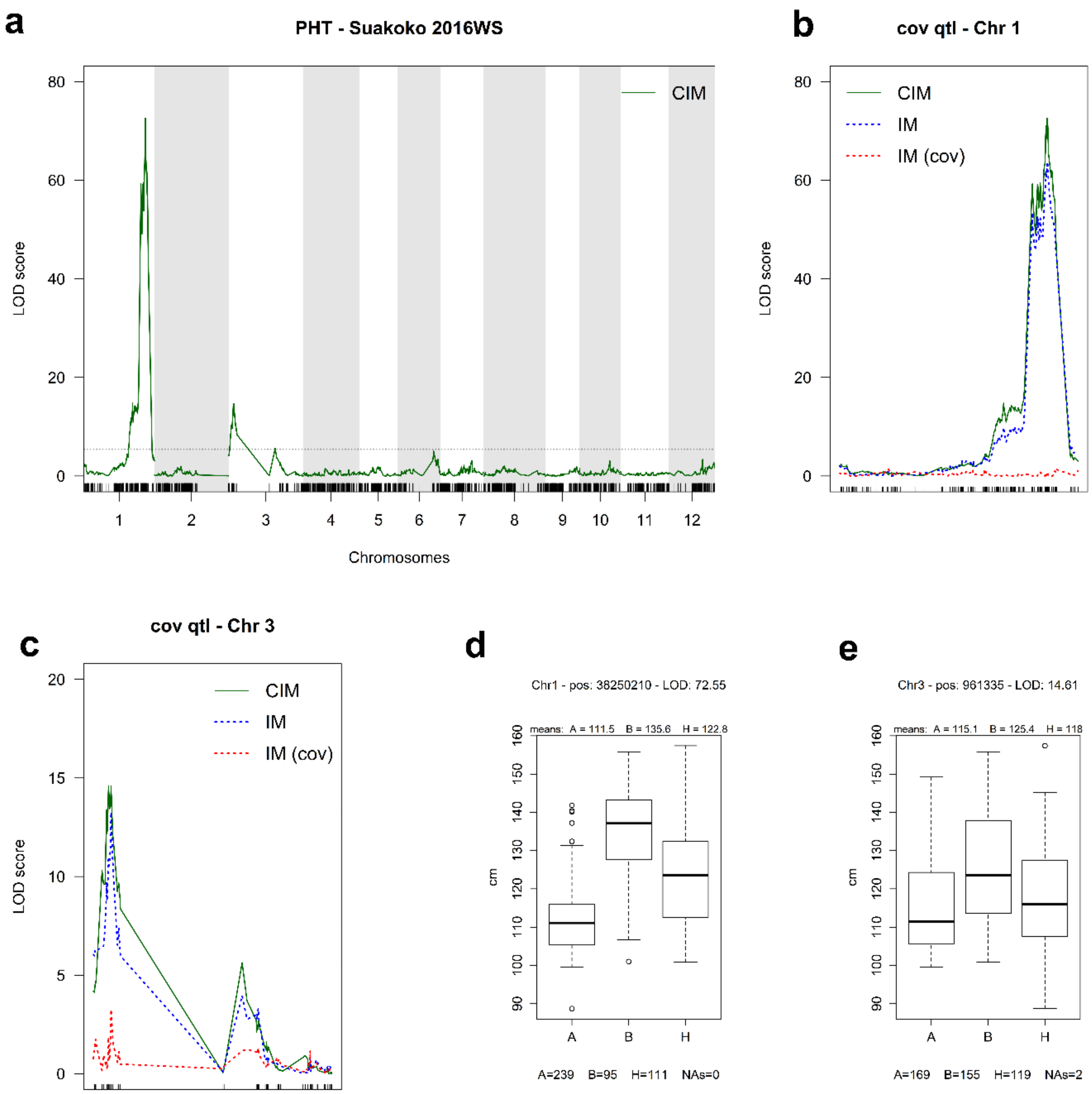

Supplementary Fig. S21. QTL mapping results of PHT for the N-L-19 x IR64-Sub1 population in Suakoko (HIA stress site) 2016WS. (a) LOD curve graph (green line) of composite interval mapping (CIM). Black-dotted line: significance LOD threshold (based on 1,000 permutations). (b-c) LOD curve graph comparison between CIM (green line), interval mapping (IM, blue line) and interval mapping by fixing the most significant marker (IM cov, red line) for the significant QTL on chromosome 1 and 3. (d-e) Boxplots representing the PHT performance of the lines carrying IR64-Sub1 (A), NERICA (B) or heterozygous (H) alleles at the locus (SNP-pos) of the most significant (LOD) marker of the chromosome 1 and 3 QTLs. Number of lines carrying A, B, H and missing (NA) alleles for the marker are reported under each boxplot graph. mean: mean phenotypic value of the A, B and H allelic groups.

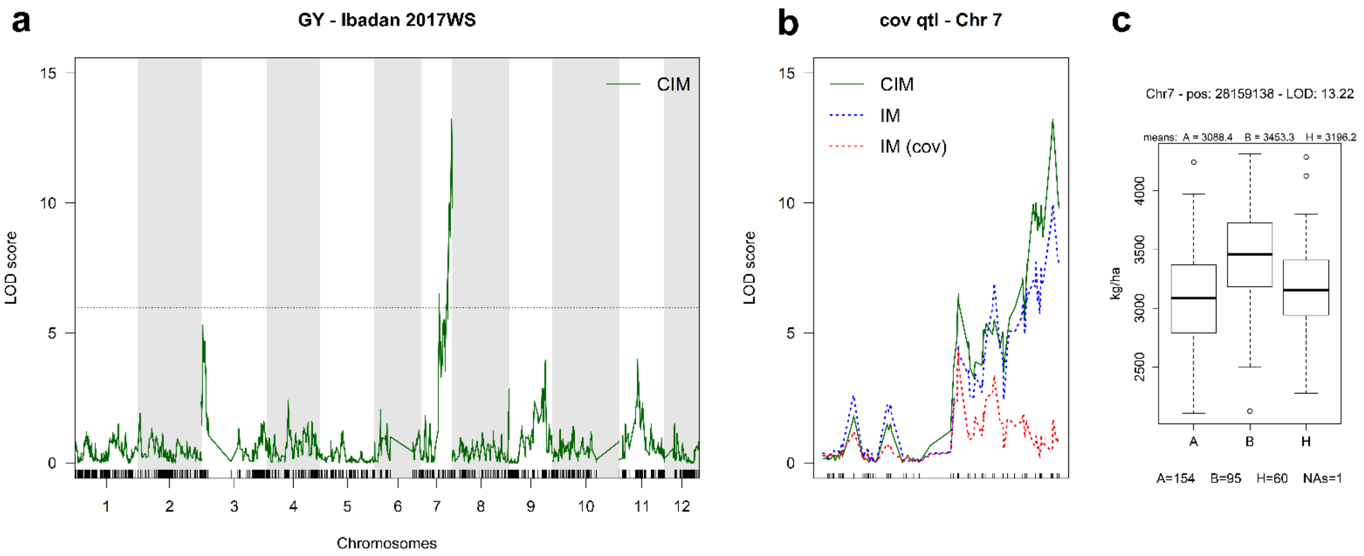

Supplementary Fig. S22. QTL mapping results of GY for the N-L-43 x IR64-Sub1 population in Ibadan (control) 2017WS. (a) LOD curve graph (green line) of composite interval mapping (CIM). Black-dotted line: significance LOD threshold (based on 1,000 permutations). (b) LOD curve graph comparison between CIM (green line), interval mapping (IM, blue line) and interval mapping by fixing the most significant marker (IM cov, red line) for the significant QTL on chromosome 7. (c) Boxplots representing the GY performance of lines carrying IR64-Sub1 (A), NERICA (B) or heterozygous (H) alleles at the locus (SNP-pos) of the most significant (LOD) marker of the chromosome 7 QTL. Number of lines carrying A, B, H and missing (NA) alleles for the marker are reported under the boxplot graph. mean: mean phenotypic value of the A, B and H allelic groups.

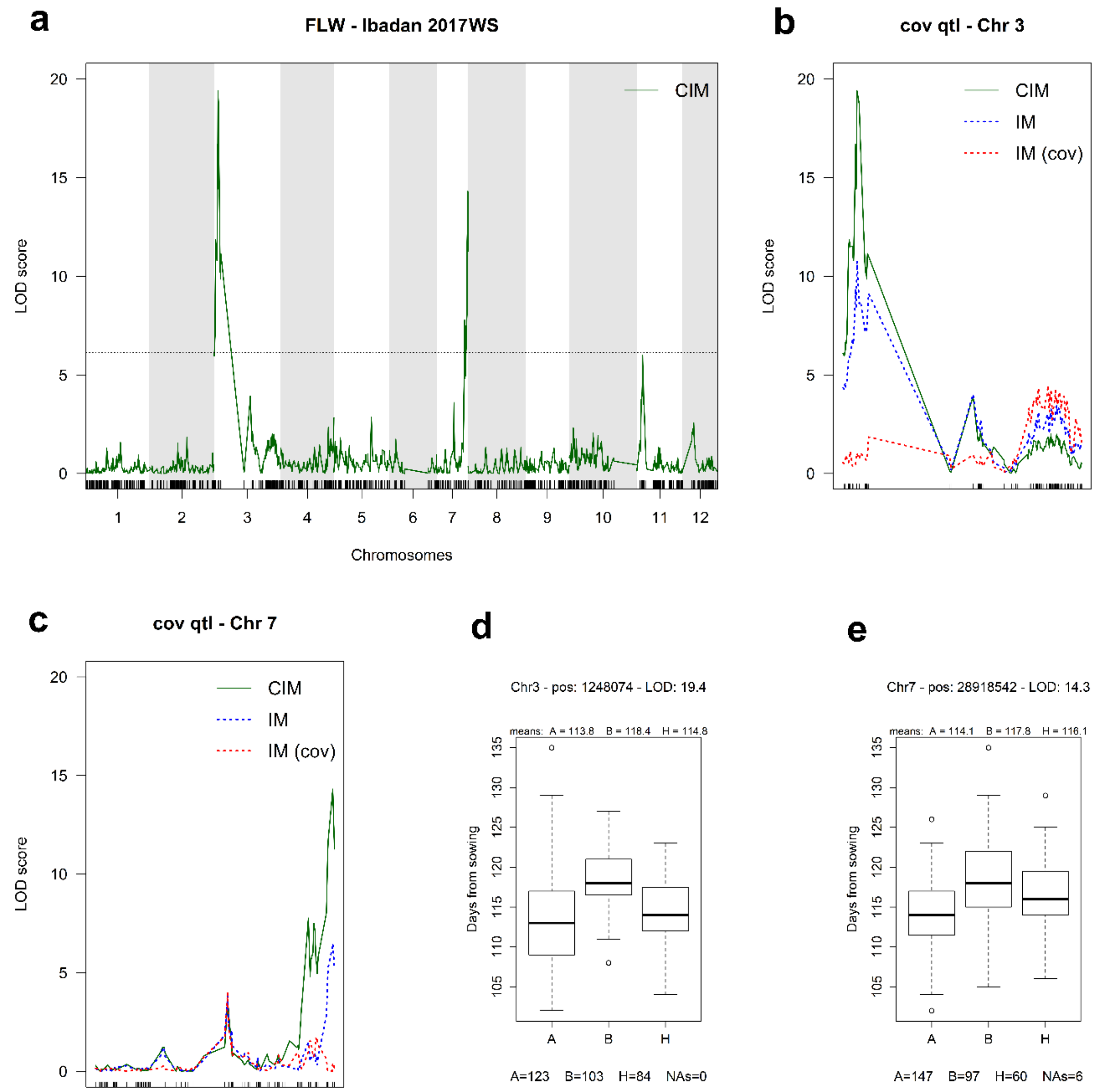

Supplementary Fig. S23. QTL mapping results of FLW for the N-L-43 x IR64-Sub1 population in Ibadan (control) 2017WS. (a) LOD curve graph (green line) of composite interval mapping (CIM). Black-dotted line: significance LOD threshold (based on 1,000 permutations). (b-c) LOD curve graph comparison between CIM (green line), interval mapping (IM, blue line) and interval mapping by fixing the most significant marker (IM cov, red line) for the significant QTL on chromosome 3 and 7. (d-e) Boxplots representing the FLW performance of the lines carrying IR64-Sub1 (A), NERICA (B) or heterozygous (H) alleles at the locus (SNP-pos) of the most significant (LOD) marker of the chromosome 3 and 7 QTLs. Number of lines carrying A, B, H and missing (NA) alleles for the marker are reported under each boxplot graph. mean: mean phenotypic value of the A, B and H allelic groups.

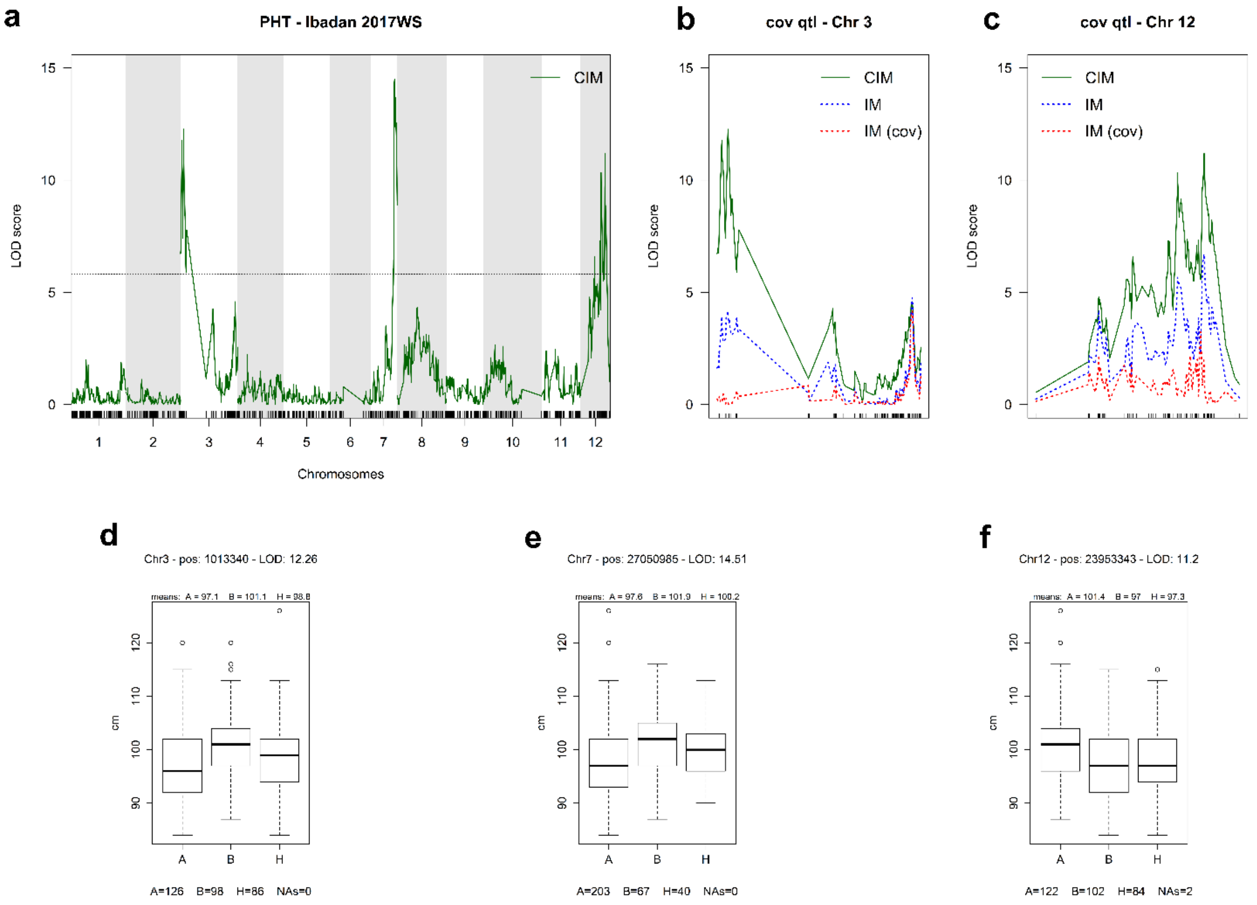

Supplementary Fig. S24. QTL mapping results of PHT for the N-L-43 x IR64-Sub1 population in Ibadan (control) 2017WS. (a) LOD curve graph (green line) of composite interval mapping (CIM). Black-dotted line: significance LOD threshold (based on 1,000 permutations). (b-c) LOD curve graph comparison between CIM (green line), interval mapping (IM, blue line) and interval mapping by fixing the most significant marker (IM cov, red line) for the significant QTL on chromosome 3 and 12. (d-f) Boxplots representing the PHT performance of the lines carrying IR64-Sub1 (A), NERICA (B) or heterozygous (H) alleles at the locus (SNP-pos) of the most significant (LOD) marker of the chromosome 3, 7, and 12 QTLs. Number of lines carrying A, B, H and missing (NA) alleles for the marker are reported under each boxplot graph. mean: mean phenotypic value of the A, B and H allelic groups.

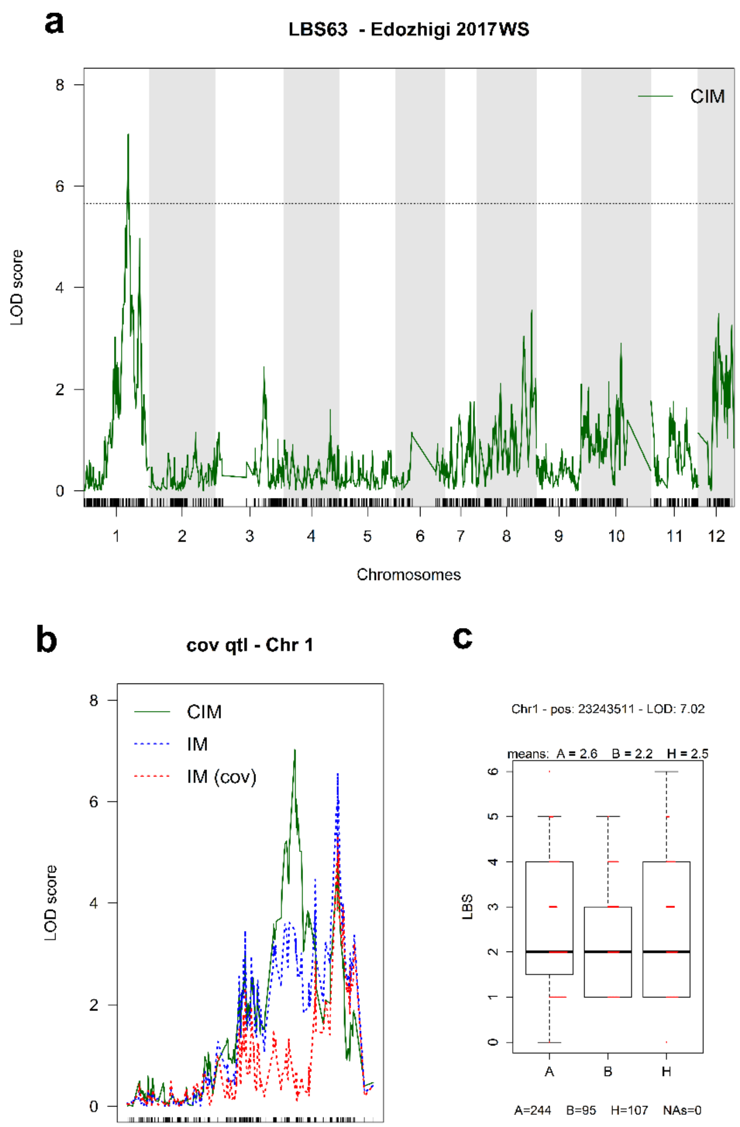

Supplementary Fig. S25. QTL mapping results of LBS63 for the N-L-43 x IR64-Sub1 population in Edozhigi (HIA stress site) 2017WS. (a) LOD curve graph (green line) of composite interval mapping (CIM). Black-dotted line: significance LOD threshold (based on 1,000 permutations). (b) LOD curve graph comparison between CIM (green line), interval mapping (IM, blue line) and interval mapping by fixing the most significant marker (IM cov, red line) for the significant QTL on chromosome 1. (c) Boxplots representing the LBS63 performance of the lines carrying IR64-Sub1 (A), NERICA (B) or heterozygous (H) alleles at the locus (SNP-pos) of the most significant (LOD) marker of the chromosome 1 QTL. Red overlay on the boxplots represents the number of accessions in each class of this categorical trait. Number of lines carrying A, B, H and missing (NA) alleles for the marker are reported under the boxplot graph. mean: mean phenotypic value of the A, B and H allelic groups.

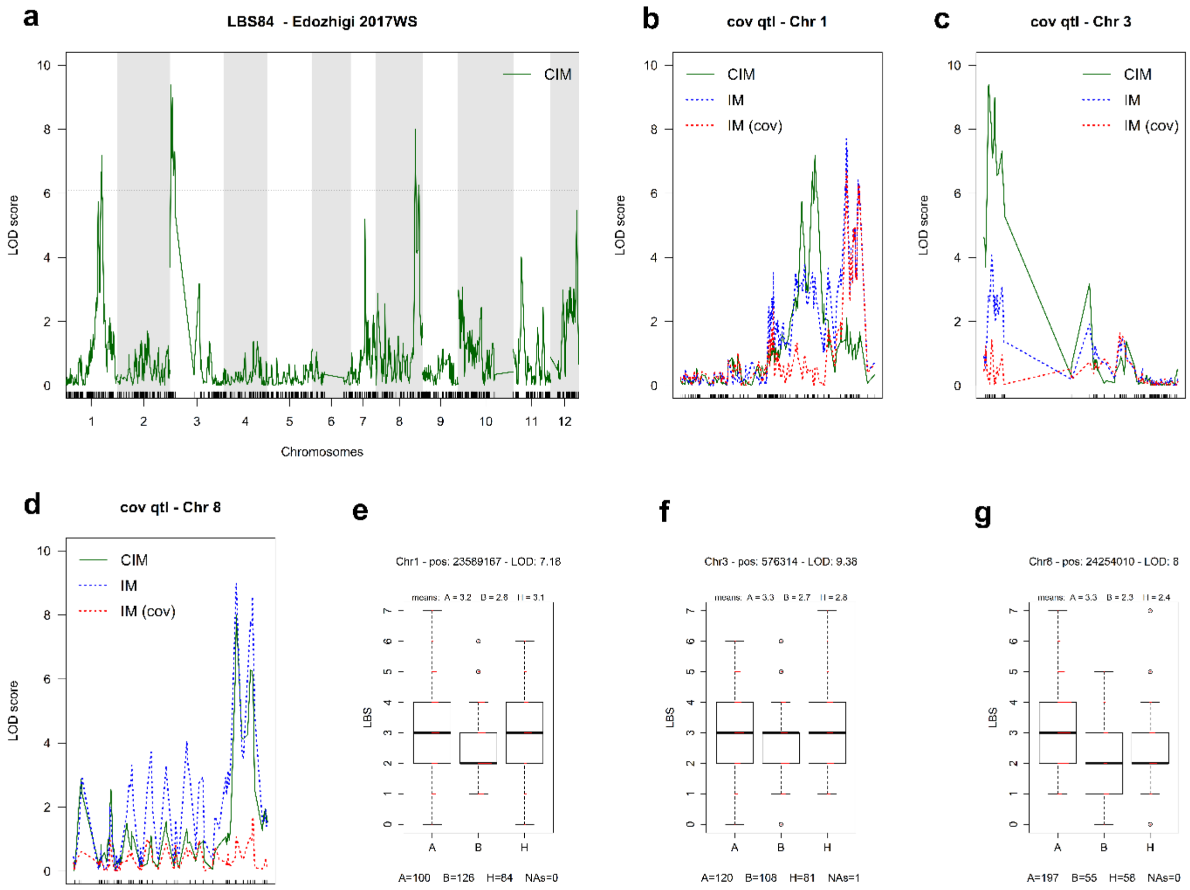

Supplementary Fig. S26. QTL mapping results of LBS84 for the N-L-43 x IR64-Sub1 population in Edozhigi (HIA stress site) 2017WS. (a) LOD curve graph (green line) of composite interval mapping (CIM). Black-dotted line: significance LOD threshold (based on 1,000 permutations). (b-d) LOD curve graph comparison between CIM (green line), interval mapping (IM, blue line) and interval mapping by fixing the most significant marker (IM cov, red line) for the significant QTL on chromosome 1, 3, and 8. (e-g) Boxplots representing the LBS84 performance of the lines carrying IR64-Sub1 (A), NERICA (B) or heterozygous (H) alleles at the locus (SNP-pos) of the most significant (LOD) marker of the chromosome 1, 3, and 8 QTLs. Red overlay on the boxplots represents the number of accessions in each class of this categorical trait. Number of lines carrying A, B, H and missing (NA) alleles for the marker are reported under the boxplot graph. mean: mean phenotypic value of the A, B and H allelic groups.

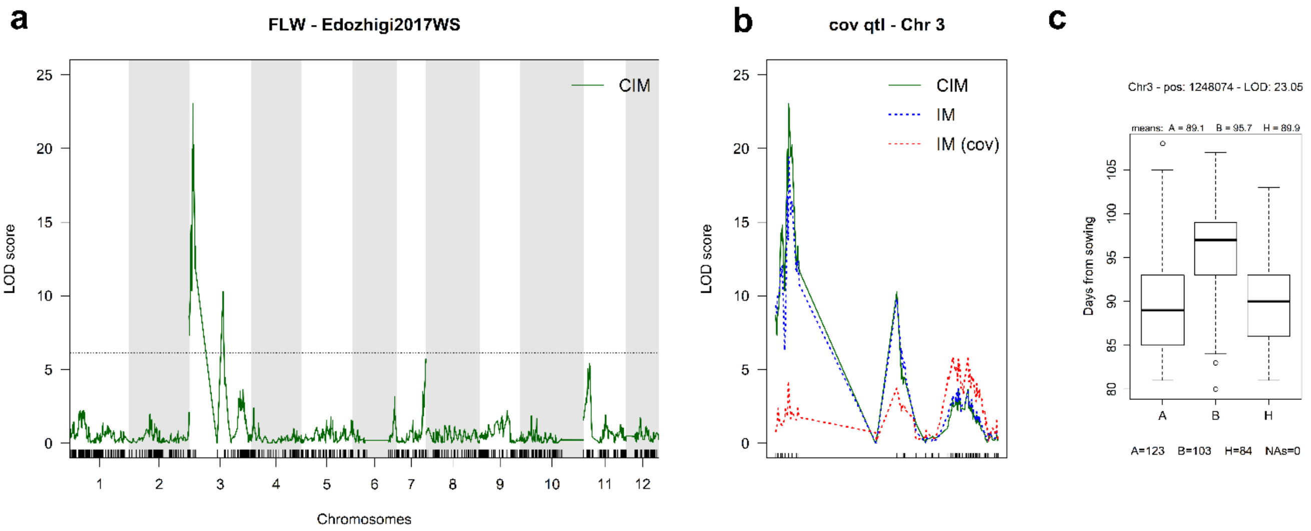

Supplementary Fig. S27. QTL mapping results of FLW for the N-L-43 x IR64-Sub1 population in Edozhigi (HIA stress site) 2017WS. (a) LOD curve graph (green line) of composite interval mapping (CIM). Black-dotted line: significance LOD threshold (based on 1,000 permutations). (b) LOD curve graph comparison between CIM (green line), interval mapping (IM, blue line) and interval mapping by fixing the most significant marker (IM cov, red line) for the significant QTL on chromosome 3. (c-d) Boxplots representing the FLW performance of the lines carrying IR64-Sub1 (A), NERICA (B) or heterozygous (H) alleles at the locus (SNP-pos) of the most significant (LOD) marker of the chromosome 3 QTL. Number of lines carrying A, B, H and missing (NA) alleles for the marker are reported under each boxplot graph. mean: mean phenotypic value of the A, B and H allelic groups.

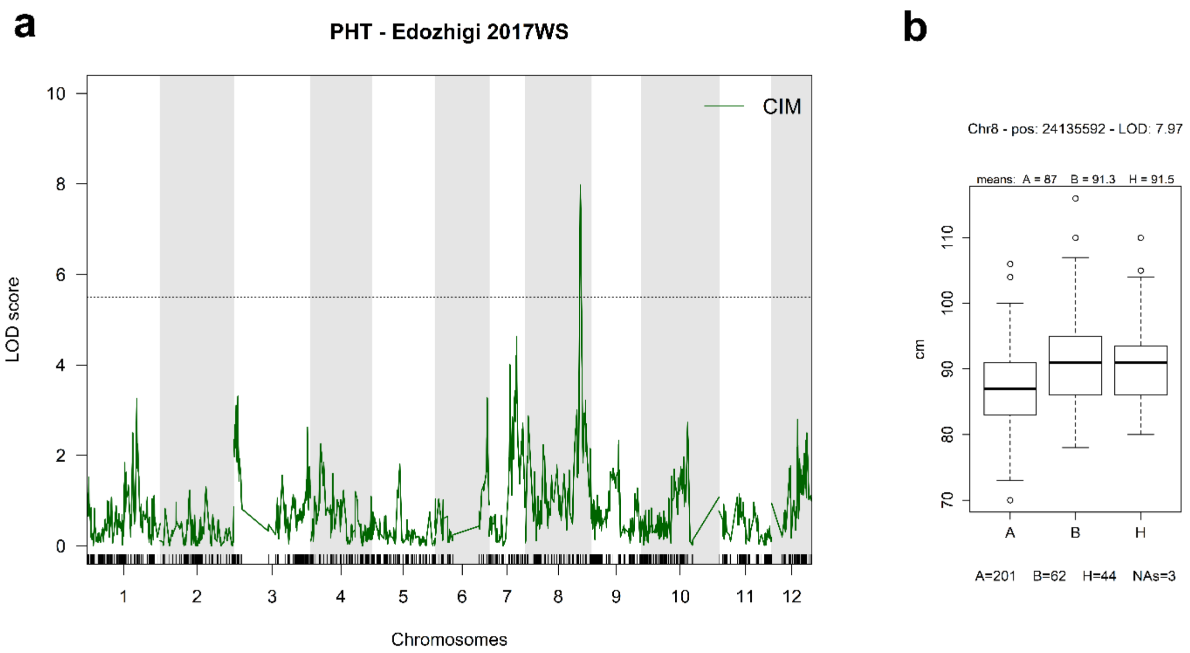

Supplementary Fig. S28. QTL mapping results of PHT for the N-L-43 x IR64-Sub1 population in Edozhigi (HIA stress site) 2017WS. (a) LOD curve graph (green line) of composite interval mapping (CIM). Black-dotted line: significance LOD threshold (based on 1,000 permutations). (b) Boxplots representing the PHT performance of the lines carrying IR64-Sub1 (A), NERICA (B) or heterozygous (H) alleles at the locus (SNP-pos) of the most significant (LOD) marker of the chromosome 8 QTL. Number of lines carrying A, B, H and missing (NA) alleles for the marker are reported under each boxplot graph. mean: mean phenotypic value of the A, B and H allelic groups.

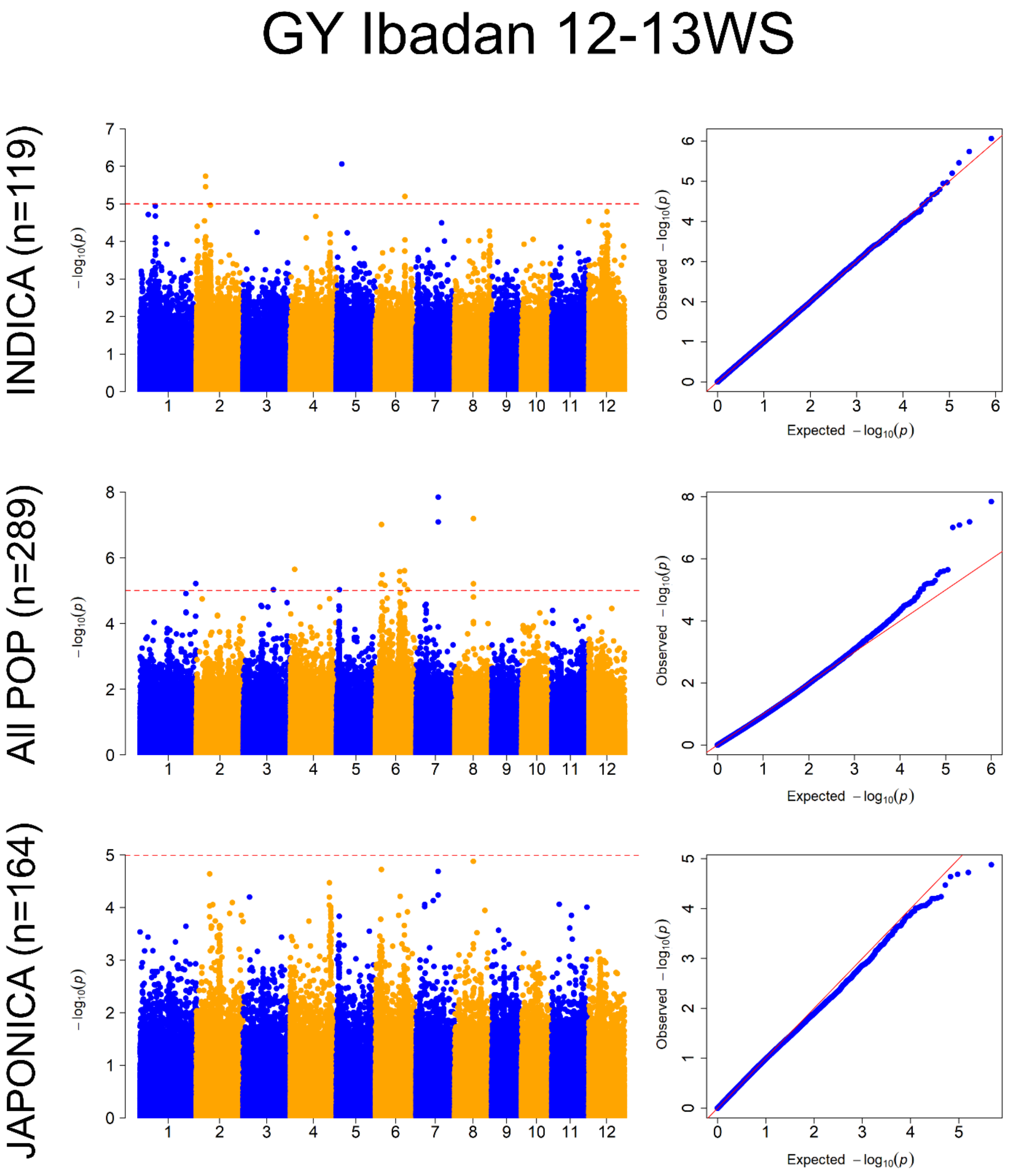

Supplementary Fig. S29. Manhattan plots (left) and Quantile-Quantile plots (right) of the GWA mapping results for grain yield (GY) in Ibadan (Nigeria) during the 2012-2013 wet seasons (12-13WS) in *AllPOP* (middle), *INDICA* (top) and *JAPONICA* (bottom) varietal groups of the RDP1 panel. n=number of accessions present in the varietal group. The red dashed line indicates the genome-wide threshold for significant associated markers ($-\log_{10} p > 5.0$).

GY Suakoko 13WS

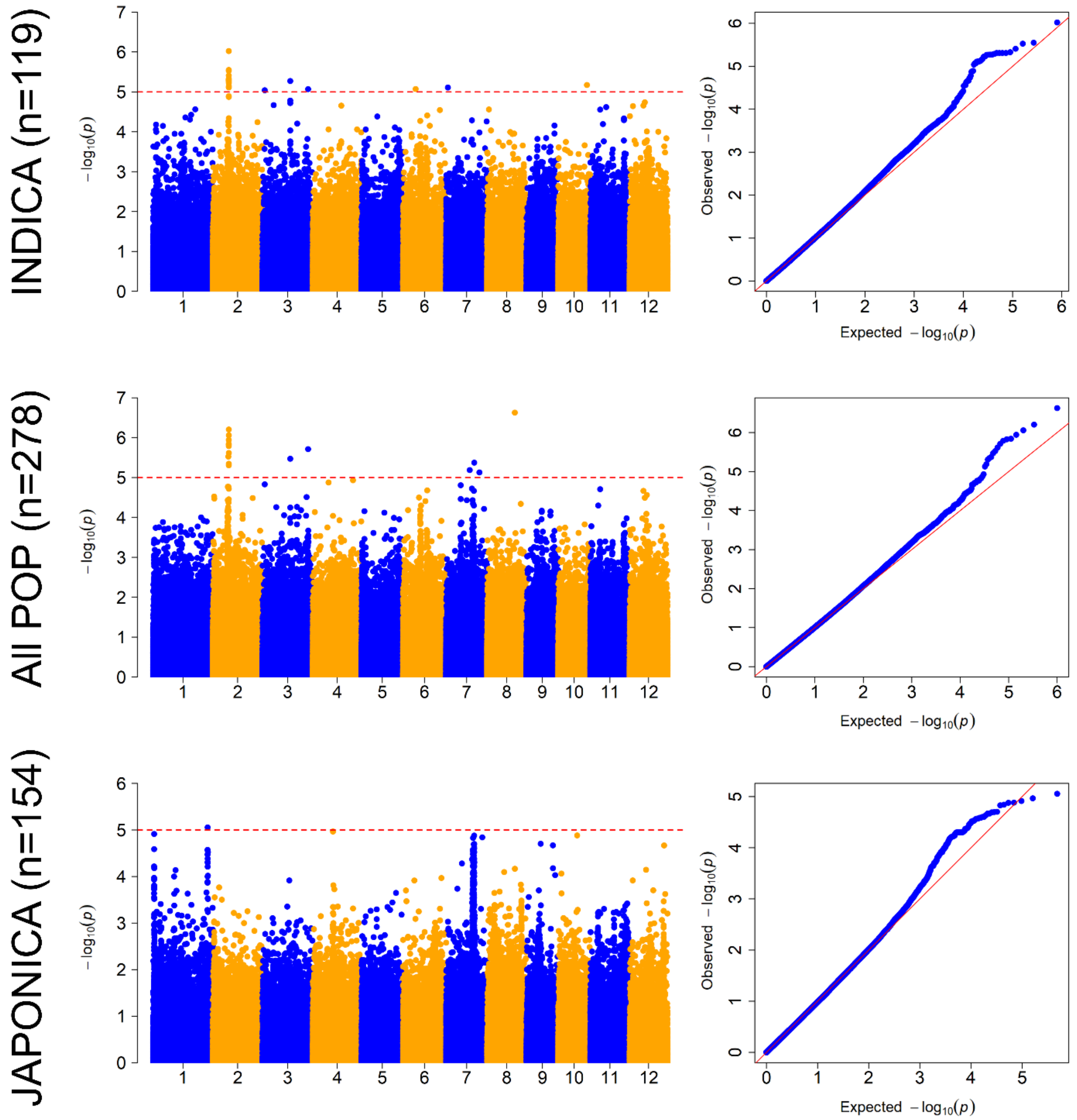

Supplementary Fig. S30. Manhattan plots (left) and Quantile-Quantile plots (right) of the GWA mapping results for grain yield (GY) in Suakoko (Liberia) during the 2013 wet season (13WS) in *AllPOP* (middle), *INDICA* (top) and *JAPONICA* (bottom) varietal groups of the RDP1 panel. n=number of accessions present in the varietal group. The red dashed line indicates the genome-wide threshold for significant associated markers ($-\log_{10} p > 5.0$).

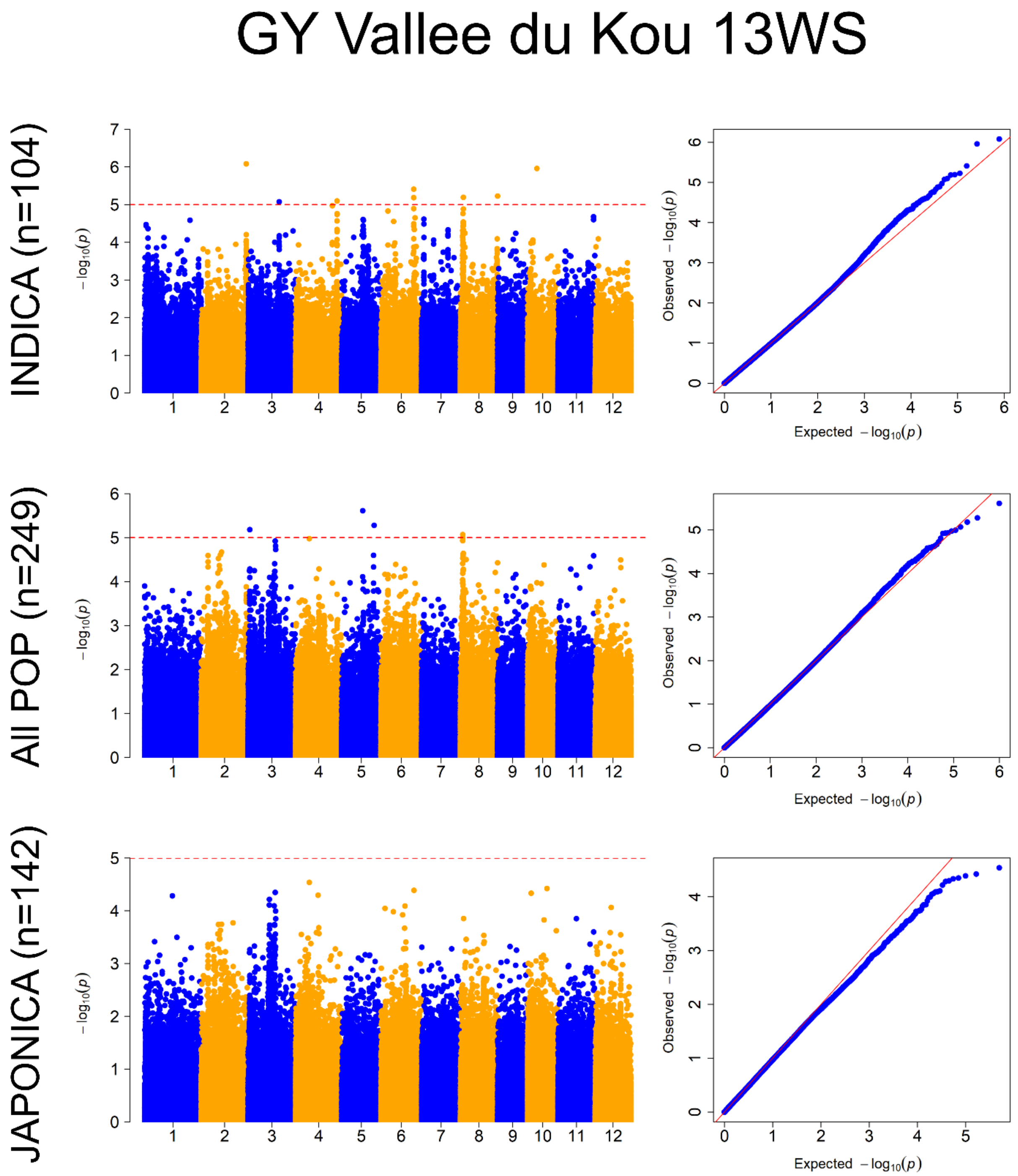

Supplementary Fig. S31. Manhattan plots (left) and Quantile-Quantile plots (right) of the GWA mapping results for grain yield (GY) in Vallee du Kou (Burkina Faso) during the 2013 wet season (13WS) in *AllPOP* (middle), *INDICA* (top) and *JAPONICA* (bottom) varietal groups of the RDP1 panel. n=number of accessions present in the varietal group. The red dashed line indicates the genome-wide threshold for significant associated markers ($-\log_{10} p > 5.0$).

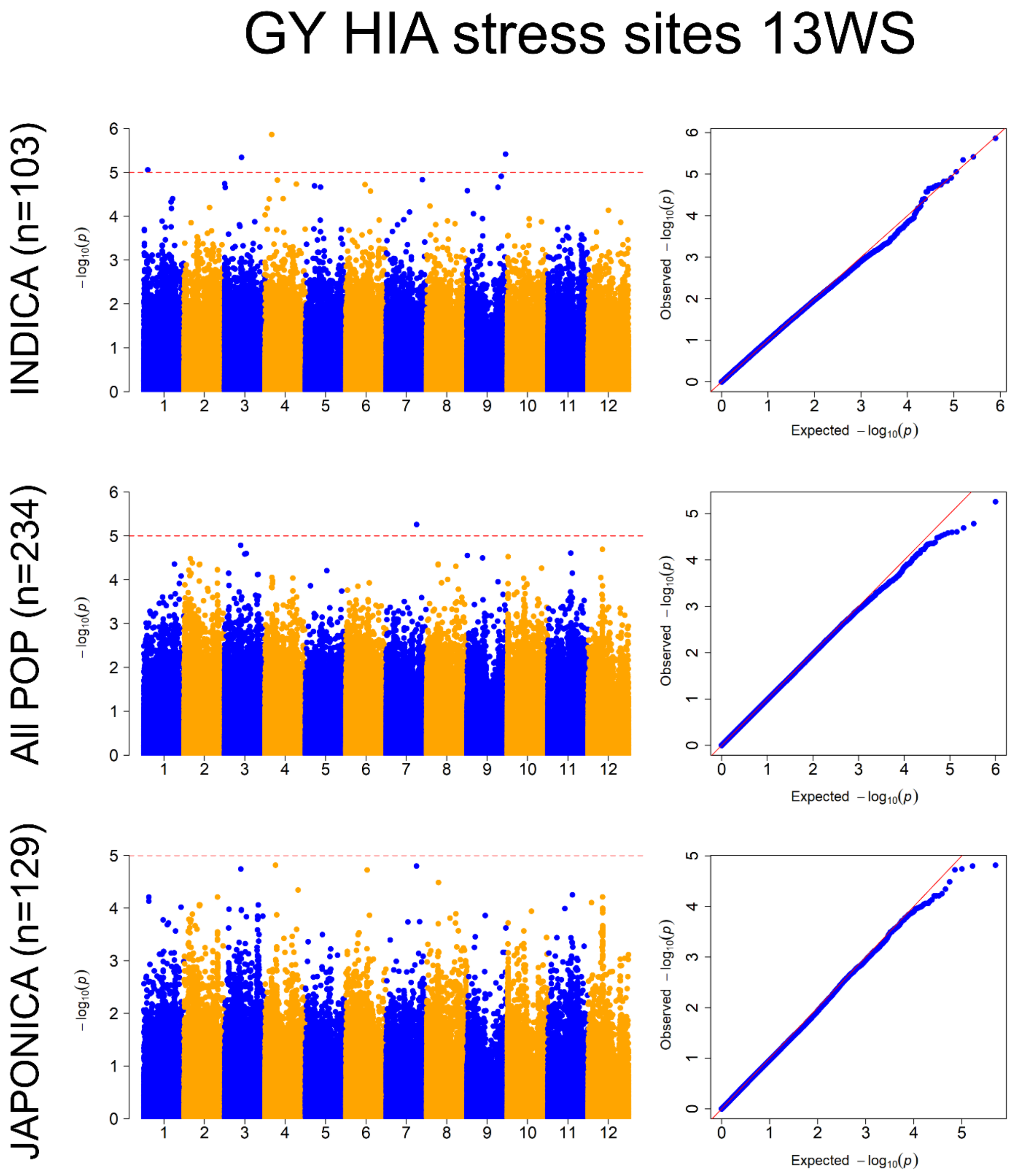

Supplementary Fig. S32. Manhattan plots (left) and Quantile-Quantile plots (right) of the GWA mapping results for grain yield (GY) in all the HIA stress sites (Suakoko and Vallee du Kou) during the 2013 wet season (13WS) in *AllPOP* (middle), *INDICA* (top) and *JAPONICA* (bottom) varietal groups of the RDP1 panel. n=number of accessions present in the varietal group. The red dashed line indicates the genome-wide threshold for significant associated markers ($-\log_{10} p > 5.0$).

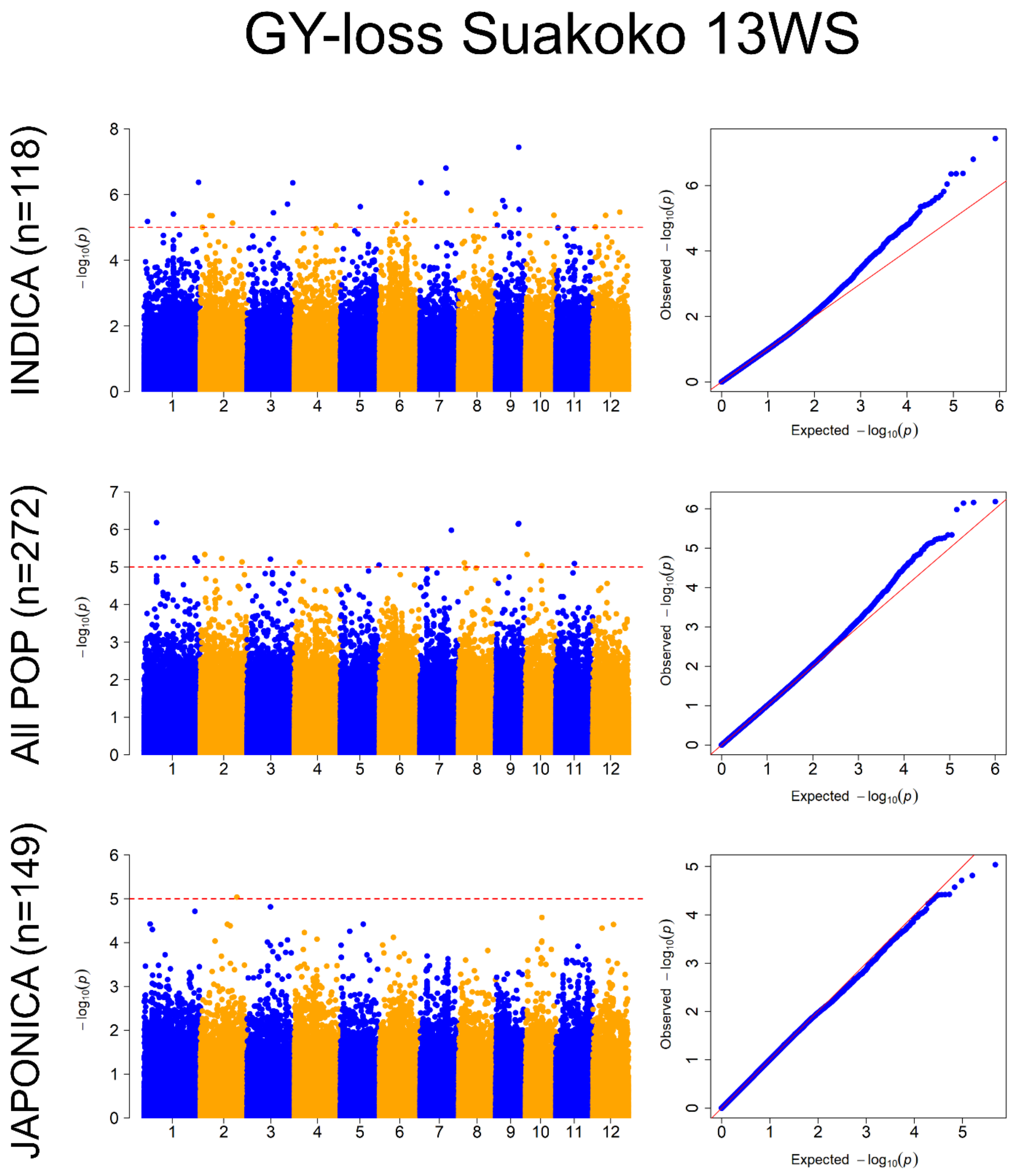

Supplementary Fig. S33. Manhattan plots (left) and Quantile-Quantile plots (right) of the GWA mapping results for grain yield loss (GY-loss) in Suakoko (Liberia) during the 2013 wet season (13WS) in *AllPOP* (middle), *INDICA* (top) and *JAPONICA* (bottom) varietal groups of the RDP1 panel. n=number of accessions present in the varietal group. The red dashed line indicates the genome-wide threshold for significant associated markers ($-\log_{10} p > 5.0$).

GY-loss Vallee du Kou 13WS

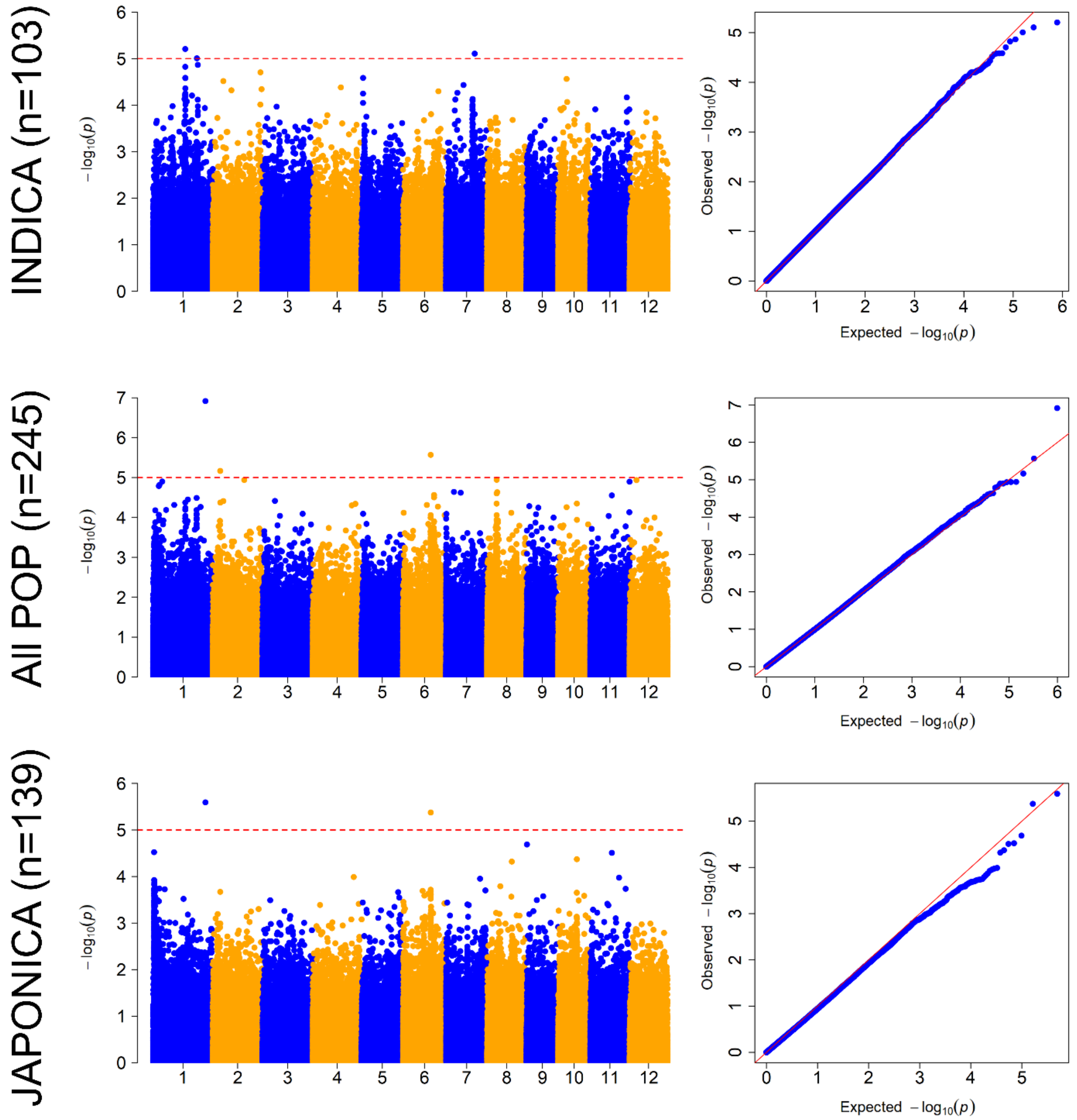

Supplementary Fig. S34. Manhattan plots (left) and Quantile-Quantile plots (right) of the GWA mapping results for grain yield loss (GY-loss) in Vallee du Kou (Burkina Faso) during the 2013 wet season (13WS) in *AllPOP* (middle), *INDICA* (top) and *JAPONICA* (bottom) varietal groups of the RDP1 panel. n=number of accessions present in the varietal group. The red dashed line indicates the genome-wide threshold for significant associated markers ($-\log_{10} p > 5.0$).

GY-loss HIA stress sites 13WS

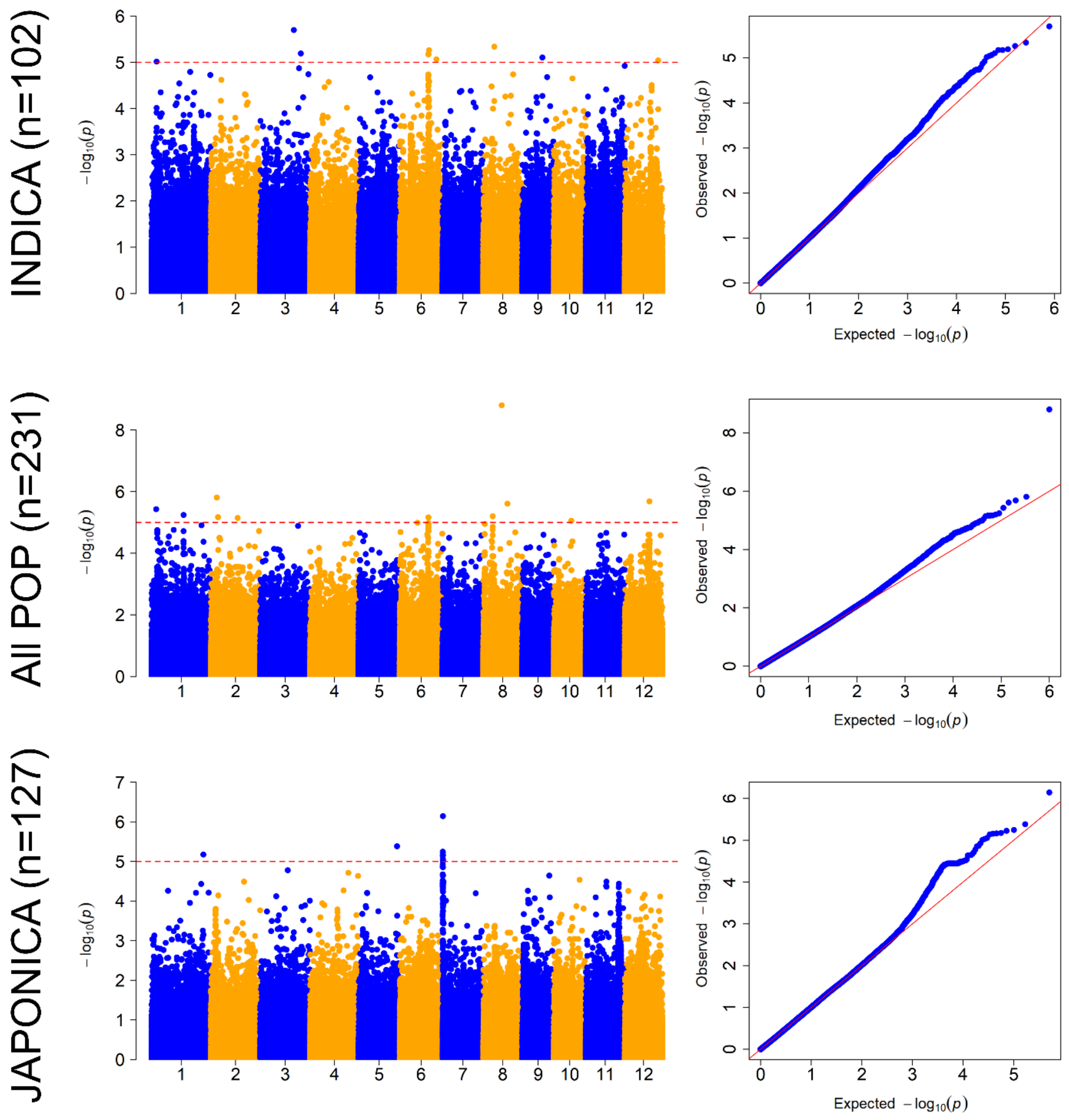

Supplementary Fig. S35. Manhattan plots (left) and Quantile-Quantile plots (right) of the GWA mapping results for grain yield loss (GY-loss) in all the HIA stress sites (Suakoko and Vallee du Kou) during the 2013 wet season (13WS) in *AllPOP* (middle), *INDICA* (top) and *JAPONICA* (bottom) varietal groups of the RDP1 panel. n=number of accessions present in the varietal group. The red dashed line indicates the genome-wide threshold for significant associated markers ($-\log_{10} p > 5.0$).

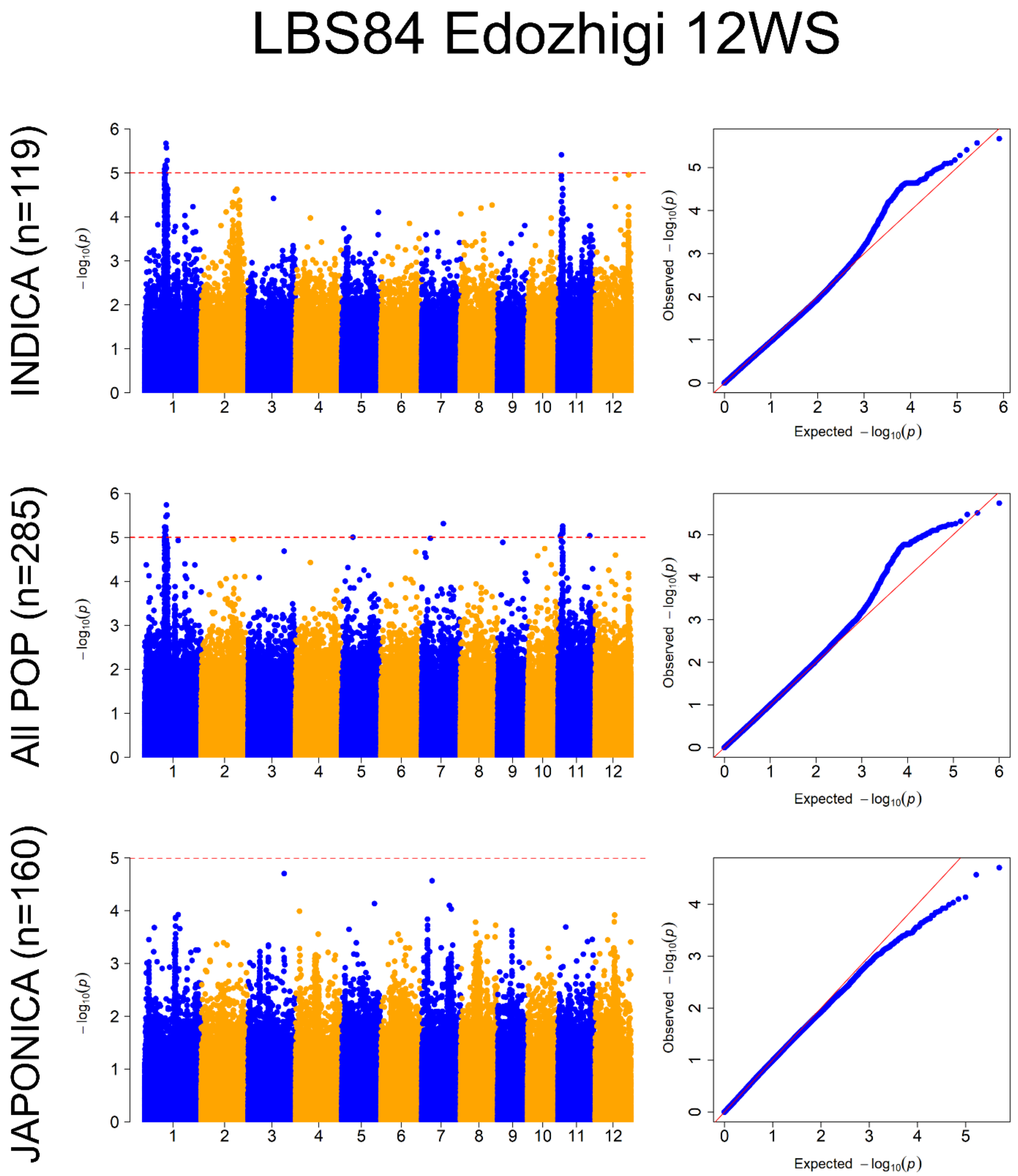

Supplementary Fig. S36. Manhattan plots (left) and Quantile-Quantile plots (right) of the GWA mapping results for leaf bronzing scores 84 days after seeding (LBS84) in Edozhigi (Nigeria) during the 2012 wet season (12WS) in *AllPOP* (middle), *INDICA* (top) and *JAPONICA* (bottom) varietal groups of the RDP1 panel. n=number of accessions present in the varietal group. The red dashed line indicates the genome-wide threshold for significant associated markers ($-\log_{10} p > 5.0$).

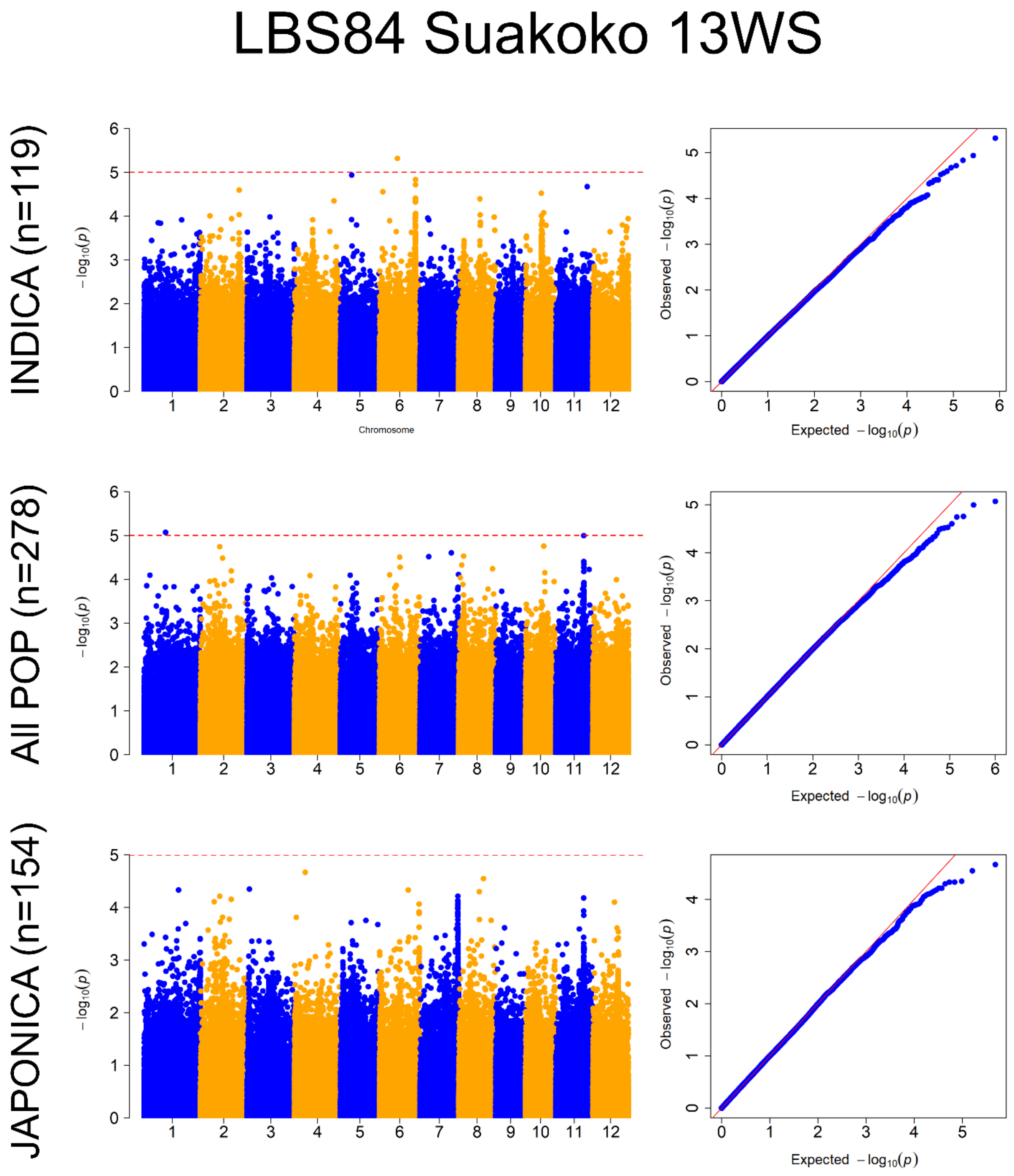

Supplementary Fig. S37. Manhattan plots (left) and Quantile-Quantile plots (right) of the GWA mapping results for leaf bronzing scores 84 days after seeding (LBS84) in Suakoko (Liberia) during the 2013 wet season (13WS) in *AllPOP* (middle), *INDICA* (top) and *JAPONICA* (bottom) varietal groups of the RDP1 panel. n=number of accessions present in the varietal group. The red dashed line indicates the genome-wide threshold for significant associated markers ($-\log_{10} p > 5.0$).

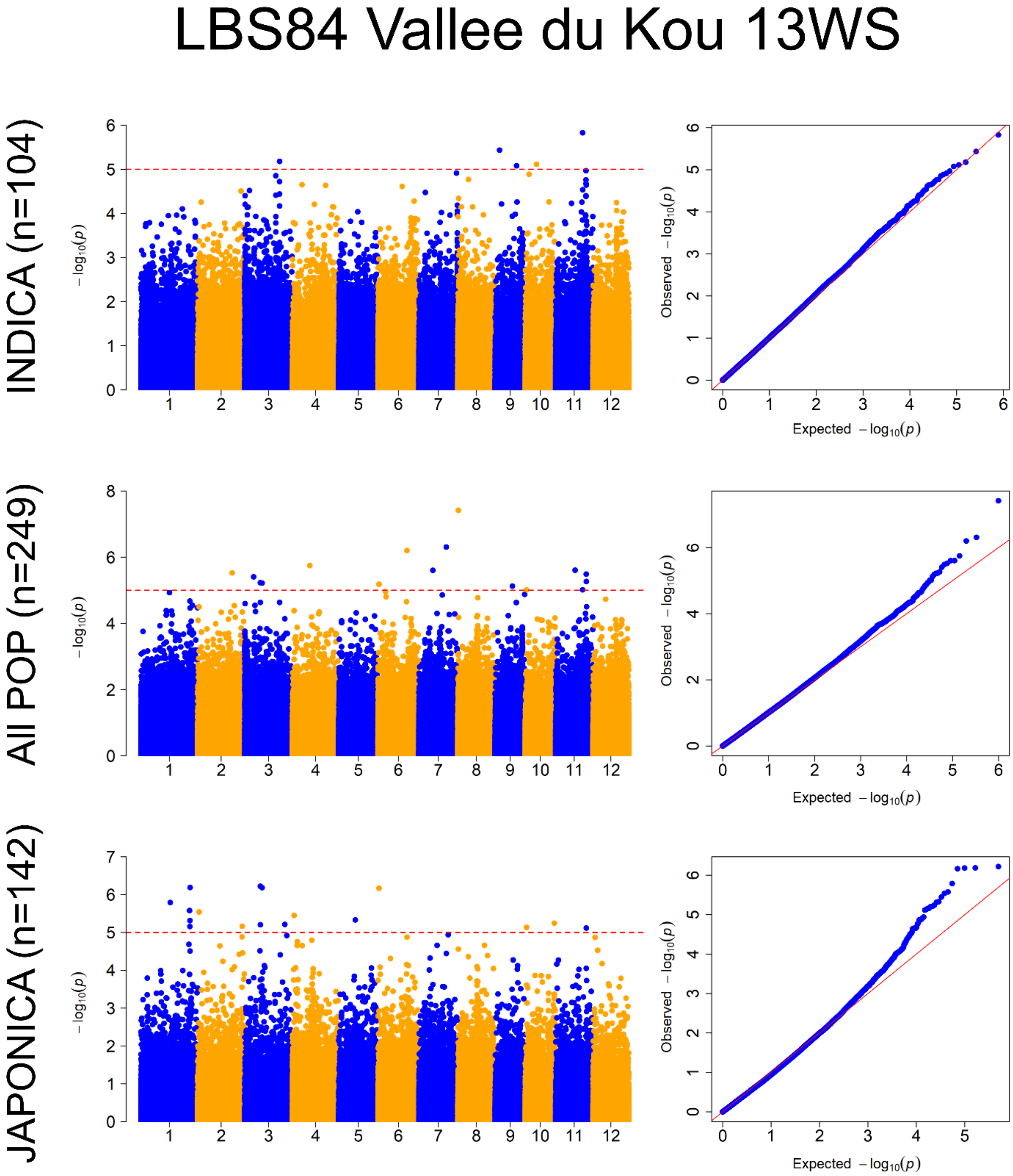

Supplementary Fig. S38. Manhattan plots (left) and Quantile-Quantile plots (right) of the GWA mapping results for leaf bronzing scores 84 days after seeding (LBS84) in Vallee du Kou (Burkina Faso) during the 2013 wet season (13WS) in *AllPOP* (middle), *INDICA* (top) and *JAPONICA* (bottom) varietal groups of the RDP1 panel. n=number of accessions present in the varietal group. The red dashed line indicates the genome-wide threshold for significant associated markers ($-\log_{10} p > 5.0$).

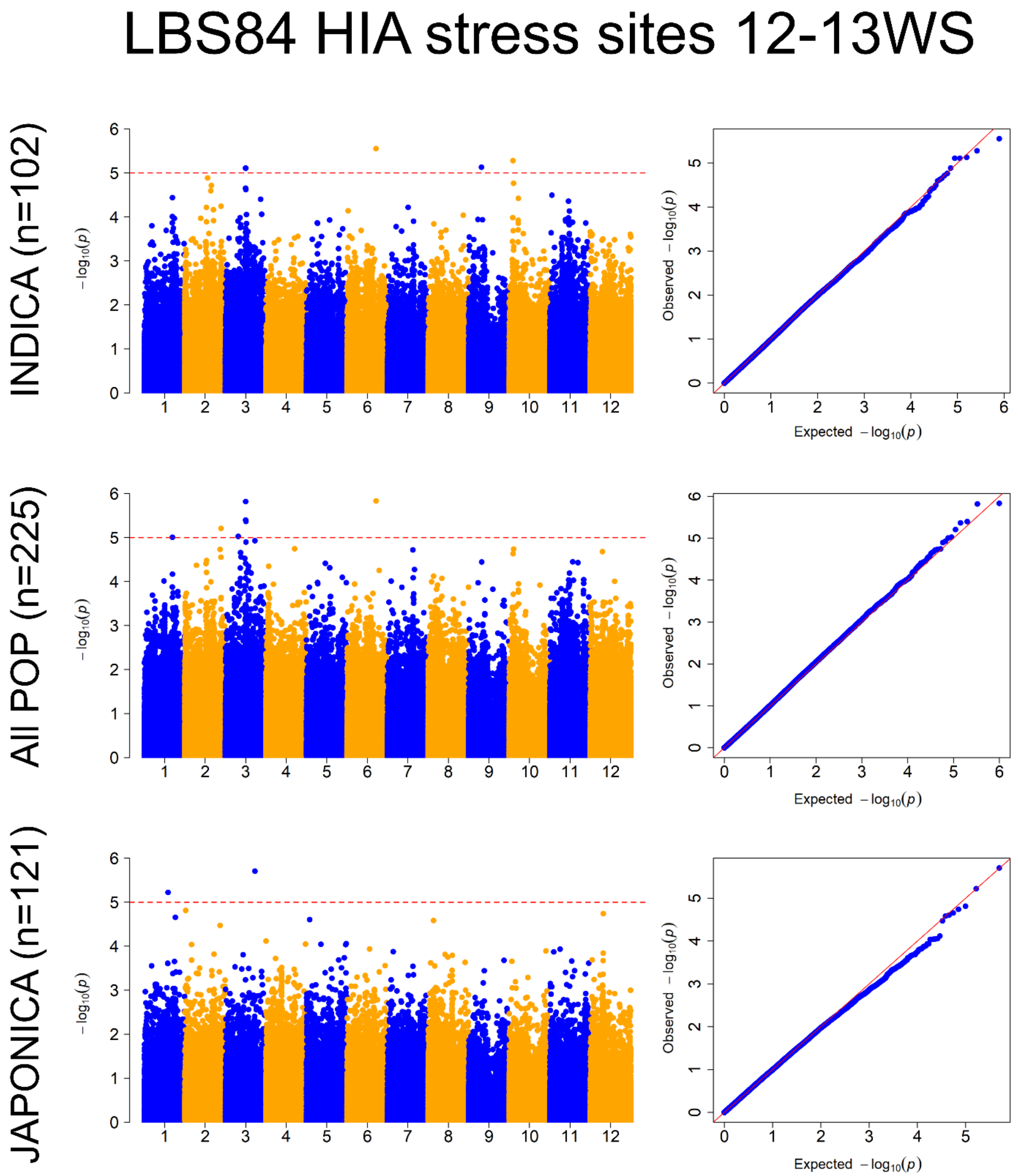

Supplementary Fig. S39. Manhattan plots (left) and Quantile-Quantile plots (right) of the GWA mapping results for leaf bronzing scores 84 days after seeding (LBS84) in all the HIA stress sites (Edozhigi, Suakoko and Vallee du Kou) during the 2012-2013 wet seasons (12-13WS) in *AllPOP* (middle), *INDICA* (top) and *JAPONICA* (bottom) varietal groups of the RDP1 panel. n=number of accessions present in the varietal group. The red dashed line indicates the genome-wide threshold for significant associated markers ($-\log_{10} p > 5.0$).

GY (Iba) - INDICA

ind
aus
adm-ind

Chr2 – pos:7,176,097 – A/T

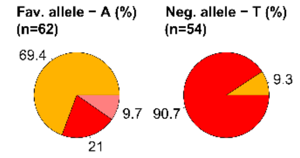

	A (%)	T (%)
ind	11.2	42.2
aus	37.1	4.3
adm-ind	5.2	0.0

Chr6 – pos:22,812,712 – C/A

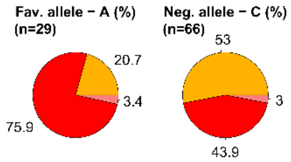

	A (%)	C (%)
ind	23.2	30.5
aus	6.3	36.8
adm-ind	1.1	2.1

GY (Iba) - AllPOP

ind
aus
adm-ind
tej
trj
adm-jap
aro
adm

Chr5 – pos:2,239,377 – T/C

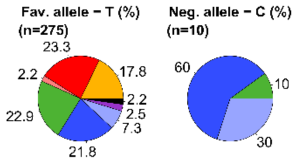

	T (%)	C (%)
ind	17.2	0.0
aus	22.5	0.0
adm-ind	2.1	0.0
tej	21.1	2.1
trj	22.1	0.4
adm-jap	7.0	1.1
aro	2.5	0.0
adm	2.1	0.0

Chr6 – pos:4,693,540 – G/A

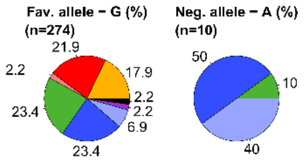

	G (%)	A (%)
ind	21.1	0.0
aus	17.3	0.0
adm-ind	2.1	0.0
tej	22.5	1.8
trj	22.5	0.4
adm-jap	6.7	1.4
aro	2.1	0.0
adm	2.1	0.0

Chr6 – pos:5,207,971 – C/T

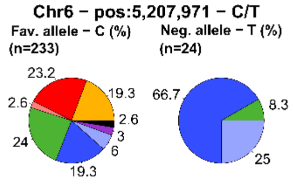

	C (%)	T (%)
ind	21.1	0.0
aus	17.5	0.0
adm-ind	2.3	0.0
tej	17.5	6.2
trj	21.8	0.8
adm-jap	5.4	2.3
aro	2.7	0.0
adm	2.3	0.0

Chr6 – pos:18,716,682 – T/C

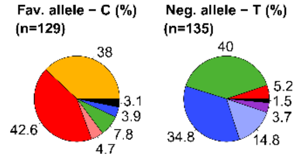

	C (%)	T (%)
ind	20.8	2.7
aus	18.6	0.0
adm-ind	2.3	0.0
tej	1.9	17.8
trj	3.8	20.5
adm-jap	0.0	7.6
aro	0.0	1.9
adm	1.5	0.8

Chr6 – pos:22,396,008 – C/T

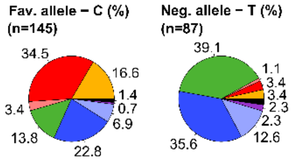

	C (%)	T (%)
ind	21.6	1.3
aus	10.3	1.3
adm-ind	2.2	0.4
tej	14.2	13.4
trj	8.6	14.7
adm-jap	4.3	4.7
aro	0.4	0.9
adm	0.9	0.9

Chr7 – pos:17,039,592 – A/G

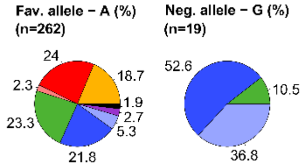

	A (%)	G (%)
ind	22.4	0.0
aus	17.4	0.0
adm-ind	2.1	0.0
tej	20.3	3.6
trj	21.7	0.7
adm-jap	5.0	2.5
aro	2.5	0.0
adm	1.8	0.0

Chr8 – pos:14,370,157 – T/C

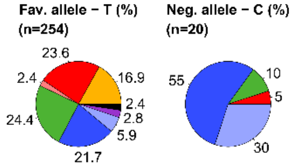

	T (%)	C (%)
ind	21.9	0.4
aus	15.7	0.0
adm-ind	2.2	0.0
tej	20.1	4.0
trj	22.6	0.7
adm-jap	5.5	2.2
aro	2.6	0.0
adm	2.2	0.0

Supplementary Fig. S40. Frequencies (%) of the favorable (Fav.) and negative (Neg.) alleles for the most significant SNPs (msSNPs) of the QTLs (see Supplementary Table S4) identified using grain yield (GY) scored in Ibadan (Iba) in *INDICA* (top) and *AllPOP* (bottom). The number of accessions carrying the two alleles (n) is indicated in brackets. Pie charts and tables show the frequencies of the Fav. and Neg. alleles in the different subpopulations. Subpopulation abbreviations: *ind=indica; aus=aus;* adm-*ind*=admixed-*indica; tej=temperate-japonica; trj=tropical-japonica;* adm-*jap*=admixed-*japonica; aro=aromatic;* adm=admixed.

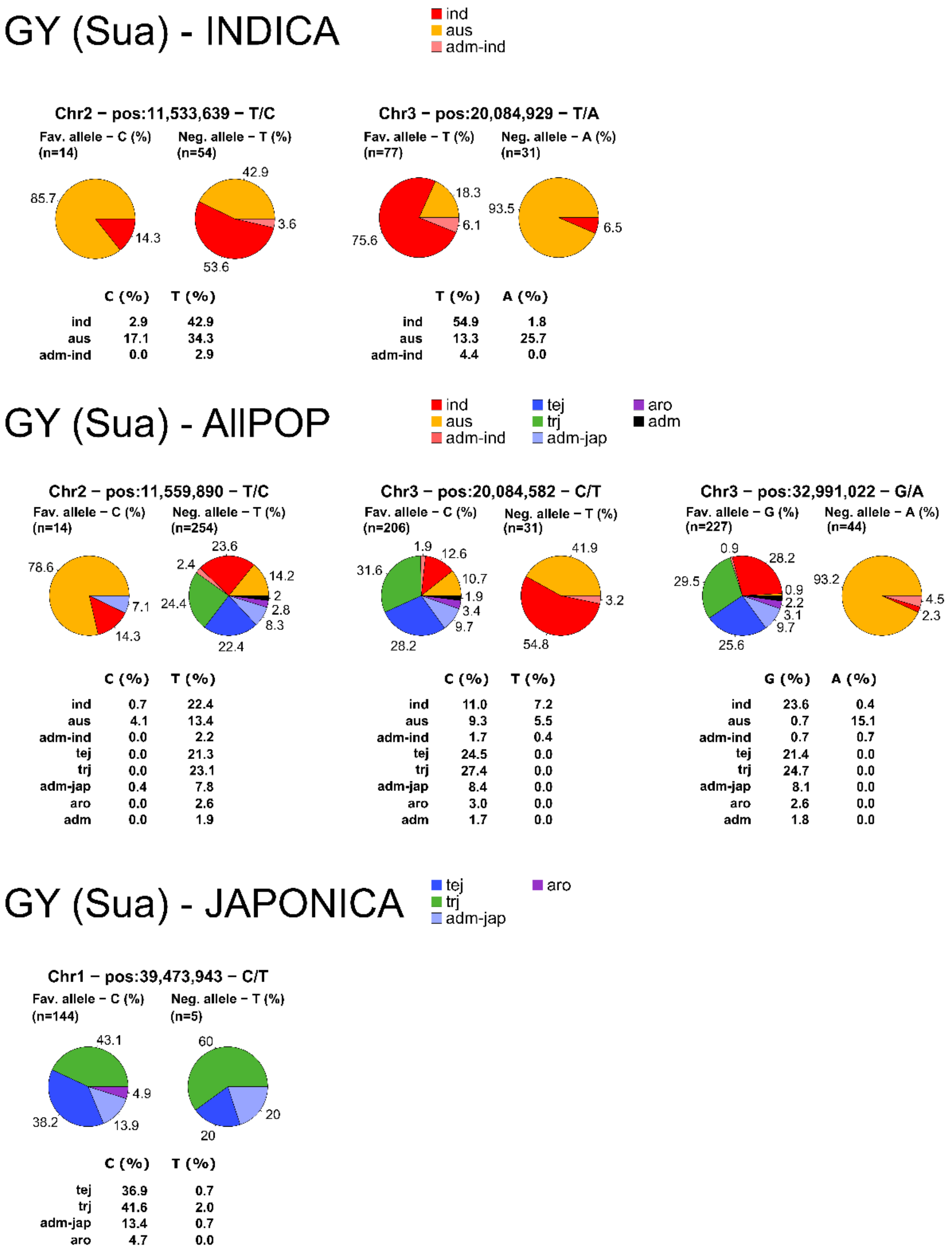

GY (Sua) - INDICA, Chr2 – pos:11,533,639 – T/C

	C (%)	T (%)
ind	2.9	42.9
aus	17.1	34.3
adm-ind	0.0	2.9

GY (Sua) - INDICA, Chr3 – pos:20,084,929 – T/A

	T (%)	A (%)
ind	54.9	1.8
aus	13.3	25.7
adm-ind	4.4	0.0

GY (Sua) - AllPOP, Chr2 – pos:11,559,890 – T/C

	C (%)	T (%)
ind	0.7	22.4
aus	4.1	13.4
adm-ind	0.0	2.2
tej	0.0	21.3
trj	0.0	23.1
adm-jap	0.4	7.8
aro	0.0	2.6
adm	0.0	1.9

GY (Sua) - AllPOP, Chr3 – pos:20,084,582 – C/T

	C (%)	T (%)
ind	11.0	7.2
aus	9.3	5.5
adm-ind	1.7	0.4
tej	24.5	0.0
trj	27.4	0.0
adm-jap	8.4	0.0
aro	3.0	0.0
adm	1.7	0.0

GY (Sua) - AllPOP, Chr3 – pos:32,991,022 – G/A

	G (%)	A (%)
ind	23.6	0.4
aus	0.7	15.1
adm-ind	0.7	0.7
tej	21.4	0.0
trj	24.7	0.0
adm-jap	8.1	0.0
aro	2.6	0.0
adm	1.8	0.0

GY (Sua) - JAPONICA, Chr1 – pos:39,473,943 – C/T

	C (%)	T (%)
tej	36.9	0.7
trj	41.6	2.0
adm-jap	13.4	0.7
aro	4.7	0.0

Supplementary Fig. S41. Frequencies (%) of the favorable (Fav.) and negative (Neg.) alleles for the most significant SNPs (msSNPs) of the QTLs (see Supplementary Table S4) identified using grain yield (GY) scored in Suakoko (Sua) in *INDICA* (top), *AllPOP* (middle) and *JAPONICA* (bottom). The number of accessions carrying the two alleles (n) is indicated in brackets. Pie charts and tables show the frequencies of the Fav. and Neg. alleles in the different subpopulations. Subpopulation abbreviations: *ind=indica; aus=aus;* adm-*ind=admixed-indica; tej=temperate-japonica; trj=tropical-japonica;* adm-*jap*=admixed-*japonica; aro=aromatic;* adm=admixed.

GY (VdK) - INDICA

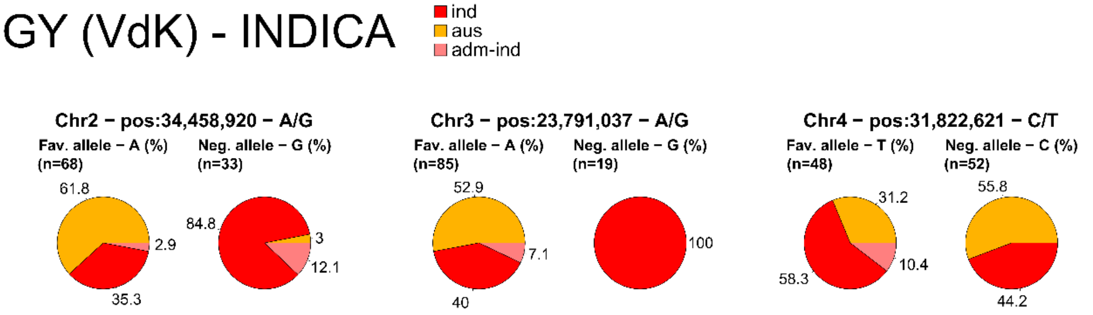

	A (%)	G (%)
ind	23.8	27.7
aus	41.6	1.0
adm-ind	2.0	4.0

	A (%)	G (%)
ind	32.7	18.3
aus	43.3	0.0
adm-ind	5.8	0.0

	T (%)	C (%)
ind	28.0	23.0
aus	15.0	29.0
adm-ind	5.0	0.0

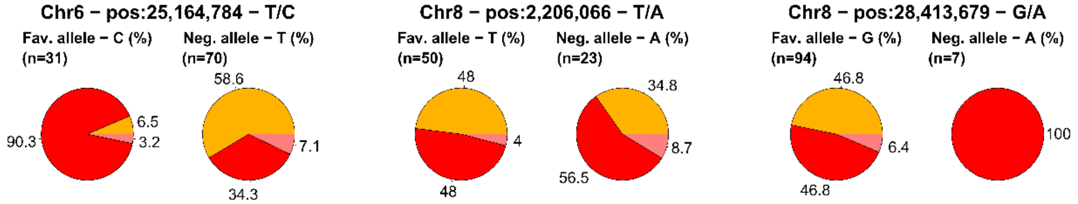

	C (%)	T (%)
ind	27.7	23.8
aus	2.0	40.6
adm-ind	1.0	5.0

	T (%)	A (%)
ind	32.9	17.8
aus	32.9	11.0
adm-ind	2.7	2.7

	G (%)	A (%)
ind	43.6	6.9
aus	43.6	0.0
adm-ind	5.9	0.0

GY (VdK) - AllPOP

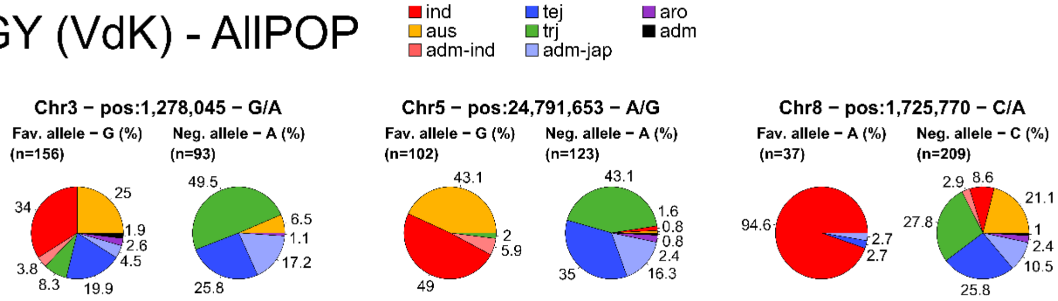

	G (%)	A (%)
ind	21.3	0.0
aus	15.7	2.4
adm-ind	2.4	0.0
tej	12.4	9.6
trj	5.2	18.5
adm-jap	2.8	6.4
aro	1.6	0.4
adm	1.2	0.0

	G (%)	A (%)
ind	22.2	0.9
aus	19.6	0.4
adm-ind	2.7	0.0
tej	0.0	19.1
trj	0.9	23.6
adm-jap	0.0	8.9
aro	0.0	1.3
adm	0.0	0.4

	A (%)	C (%)
ind	14.2	7.3
aus	0.0	17.9
adm-ind	0.0	2.4
tej	0.4	22.0
trj	0.0	23.6
adm-jap	0.4	8.9
aro	0.0	2.0
adm	0.0	0.8

Supplementary Fig. S42. Frequencies (%) of the favorable (Fav.) and negative (Neg.) alleles for the most significant SNPs (msSNPs) of the QTLs (see Supplementary Table S4) identified using grain yield (GY) scored in Vallee du Kou (VdK) in *INDICA* (top) and *AllPOP* (bottom). The number of accessions carrying the two alleles (n) is indicated in brackets. Pie charts and tables show the frequencies of the Fav. and Neg. alleles in the different subpopulations Subpopulation abbreviations: *ind=indica; aus=aus;* adm-*ind*=admixed-*indica; tej=temperate-japonica; trj=tropical-japonica;* adm-*jap*=admixed-*japonica; aro=aromatic*; adm=admixed.

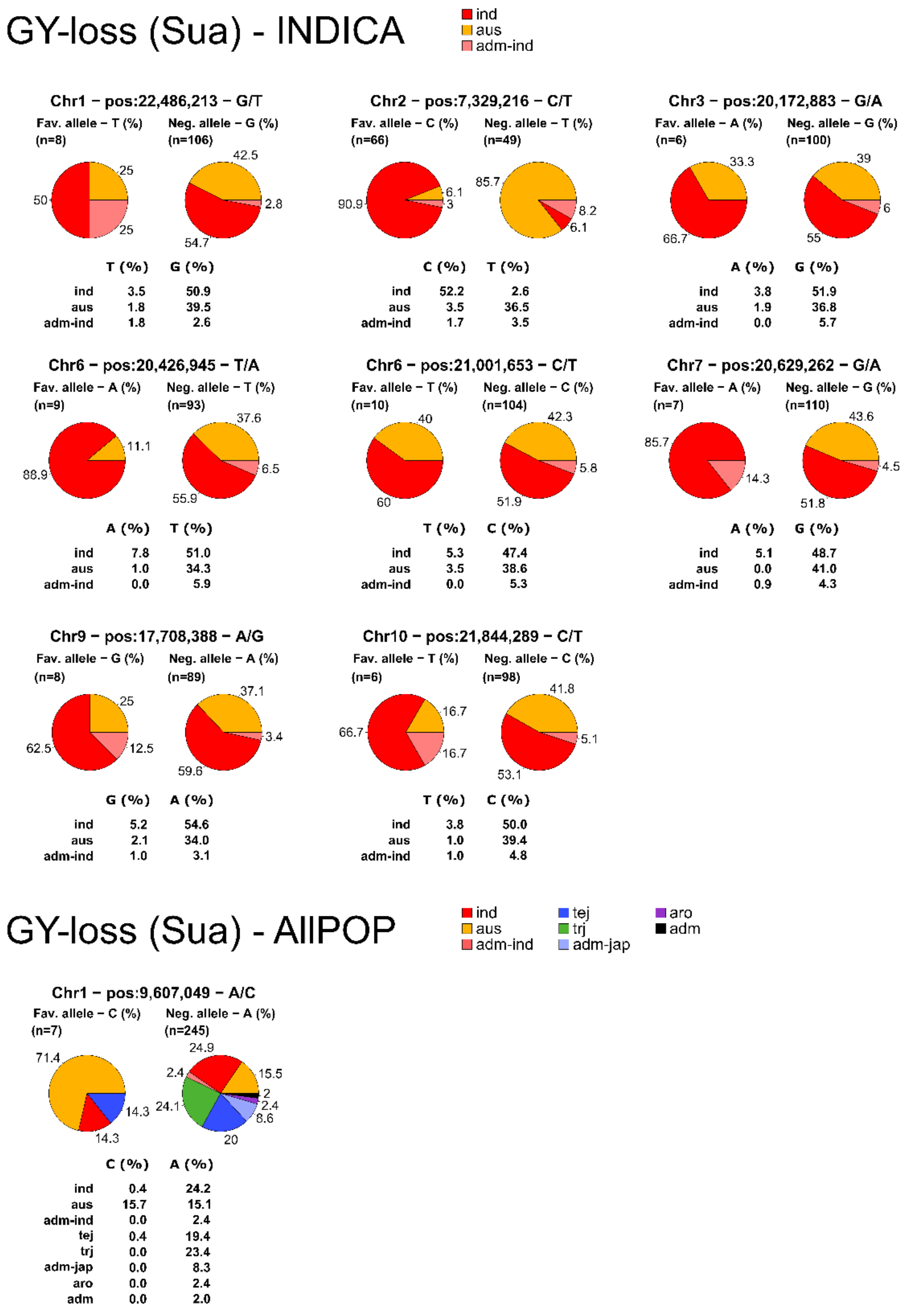

Supplementary Fig. S43. Frequencies (%) of the favorable (Fav.) and negative (Neg.) alleles for the most significant SNPs (msSNPs) of the QTLs (see Supplementary Table S4) identified using grain yield loss (GY-loss) scored in Suakoko (Sua) in *INDICA* (top) and *AllPOP* (bottom). The number of accessions carrying the two alleles (n) is indicated in brackets. Pie charts and tables show the frequencies of the Fav. and Neg. alleles in the different subpopulations Subpopulation abbreviations: *ind=indica; aus=aus;* adm-*ind*=admixed-*indica; tej=temperate-japonica; trj=tropical-japonica;* adm-*jap*=admixed-*japonica; aro=aromatic*; adm=admixed.

GY-loss (VdK) - INDICA

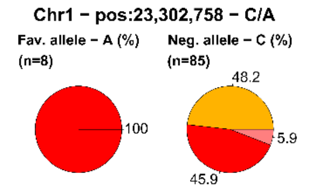

	A (%)	C (%)
ind	8.6	41.9
aus	0.0	44.1
adm-ind	0.0	5.4

GY-loss (VdK) - AllPOP

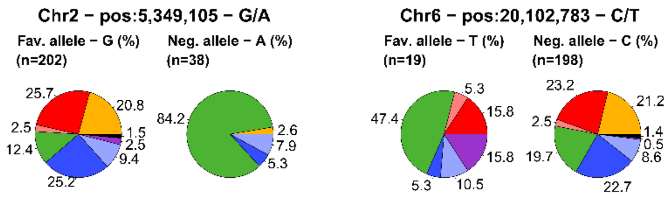

	G (%)	A (%)
ind	21.7	0.0
aus	17.5	0.4
adm-ind	2.1	0.0
tej	21.3	0.8
trj	10.4	13.3
adm-jap	7.9	1.3
aro	2.1	0.0
adm	1.3	0.0

	T (%)	C (%)
ind	1.4	21.2
aus	0.0	19.4
adm-ind	0.5	2.3
tej	0.5	20.7
trj	4.1	18.0
adm-jap	0.9	7.8
aro	1.4	0.5
adm	0.0	1.4

GY-loss (VdK) - JAPONICA

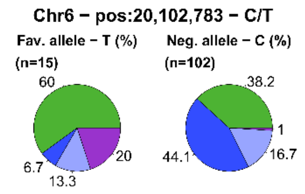

	T (%)	C (%)
tej	0.9	38.5
trj	7.7	33.3
adm-jap	1.7	14.5
aro	2.6	0.9

Supplementary Fig. S44. Frequencies (%) of the favorable (Fav.) and negative (Neg.) alleles for the most significant SNPs (msSNPs) of the QTLs (see Supplementary Table S4) identified using grain yield loss (GY-loss) scored in Vallee du Kou (VdK) in *INDICA* (top), *AllPOP* (middle) and *JAPONICA* (bottom). The number of accessions carrying the two alleles (n) is indicated in brackets. Pie charts and tables show the frequencies of the Fav. and Neg. alleles in the different subpopulations Subpopulation abbreviations: *ind=indica; aus=aus;* adm-*ind*=admixed-*indica; tej=temperate-japonica; trj=tropical-japonica;* adm-*jap*=admixed-*japonica; aro=aromatic*; adm=admixed.

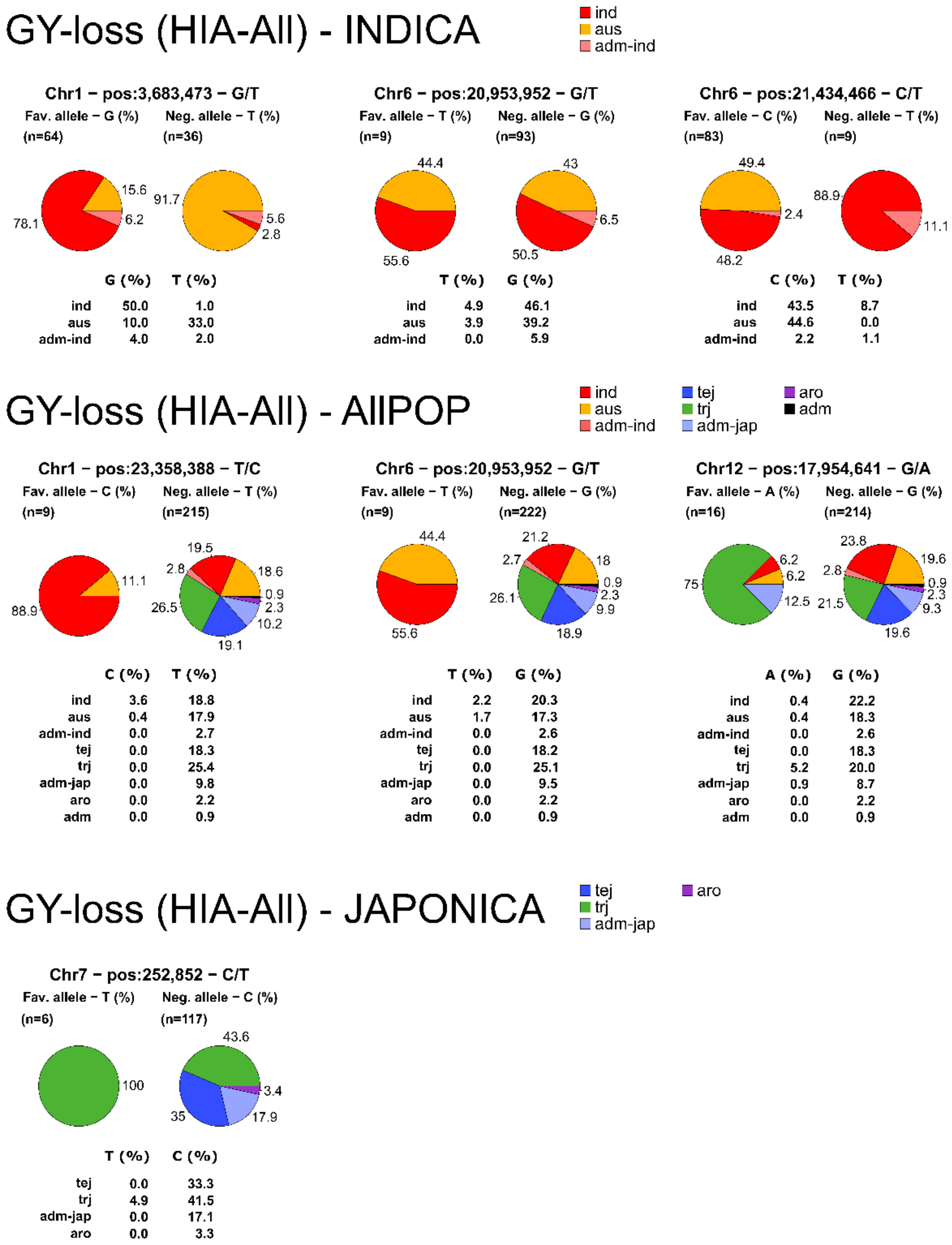

Supplementary Fig. S45. Frequencies (%) of the favorable (Fav.) and negative (Neg.) alleles for the most significant SNPs (msSNPs) of the QTLs (see Supplementary Table S4) identified using grain yield loss (GY-loss) scored in all the HIA stress sites (HIA-All) in *INDICA* (top), *AllPOP* (middle) and *JAPONICA* (bottom). The number of accessions carrying the two alleles (n) is indicated in brackets. Pie charts and tables show the frequencies of the Fav. and Neg. alleles in the different subpopulations Subpopulation abbreviations: *ind=indica; aus=aus;* adm-*ind*=admixed-*indica; tej=temperate-japonica; trj=tropical-japonica;* adm-*jap*=admixed-*japonica; aro=aromatic*; adm=admixed.

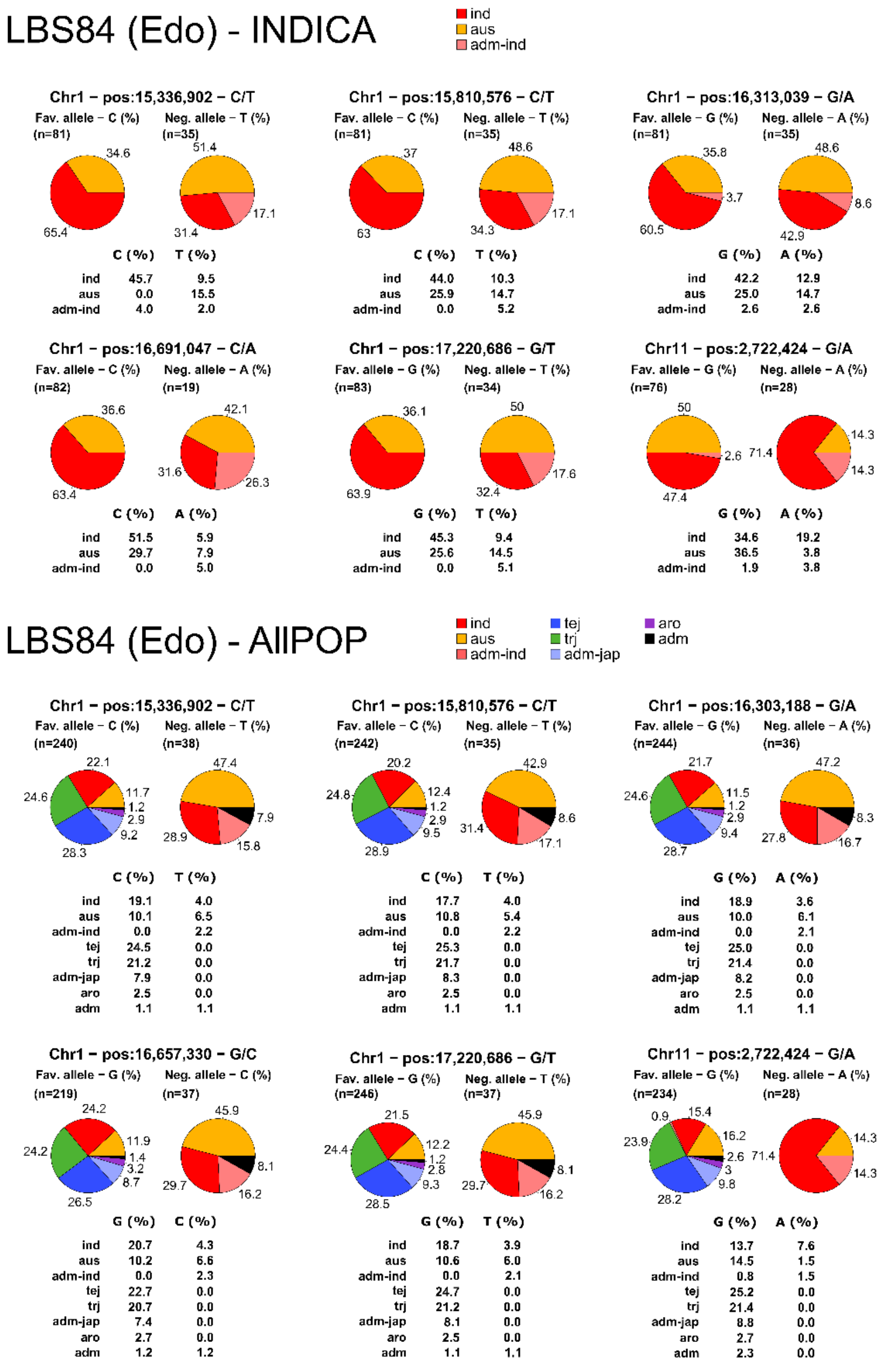

Supplementary Fig. S46. Frequencies (%) of the favorable (Fav.) and negative (Neg.) alleles for the most significant SNPs (msSNPs) of the QTLs (see Supplementary Table S4) identified using leaf bronzing scores 84 days after seeding (LBS84) in Edozhigi (Edo) in *INDICA* (top) and *AllPOP* (bottom). The number of accessions carrying the two alleles (n) is indicated in brackets. Pie charts and tables show the frequencies of the Fav. and Neg. alleles in the different subpopulations Subpopulation abbreviations: *ind=indica; aus=aus;* adm-*ind*=admixed-*indica; tej=temperate-japonica; trj=tropical-japonica;* adm-*jap*=admixed-*japonica; aro=aromatic*; adm=admixed.

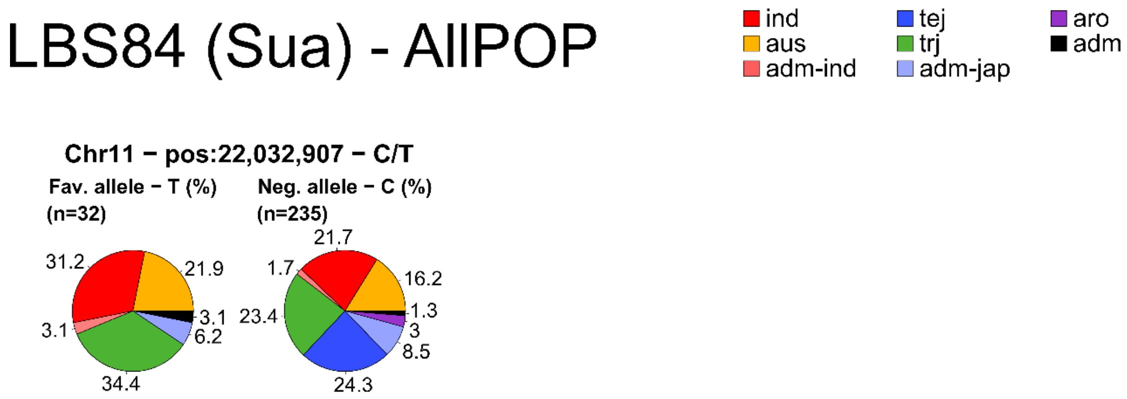

	T (%)	C (%)
ind	3.7	19.1
aus	2.6	14.2
adm-ind	0.4	1.5
tej	0.0	21.3
trj	4.1	20.6
adm-jap	0.7	7.5
aro	0.0	2.6
adm	0.4	1.1

Supplementary Fig. S47. Frequencies (%) of the favorable (Fav.) and negative (Neg.) alleles for the most significant SNP (msSNP) of the QTL (see Supplementary Table S4) identified using leaf bronzing scores 84 days after seeding (LBS84) in Suakoko (Sua) in *AllPOP*. The number of accessions carrying the two alleles (n) is indicated in brackets. Pie charts and tables show the frequencies of the Fav. and Neg. alleles in the different subpopulations Subpopulation abbreviations: *ind=indica; aus=aus;* adm-*ind*=admixed-*indica; tej=temperate-japonica; trj=tropical-japonica;* adm-*jap*=admixed-*japonica; aro=aromatic*; adm=admixed.

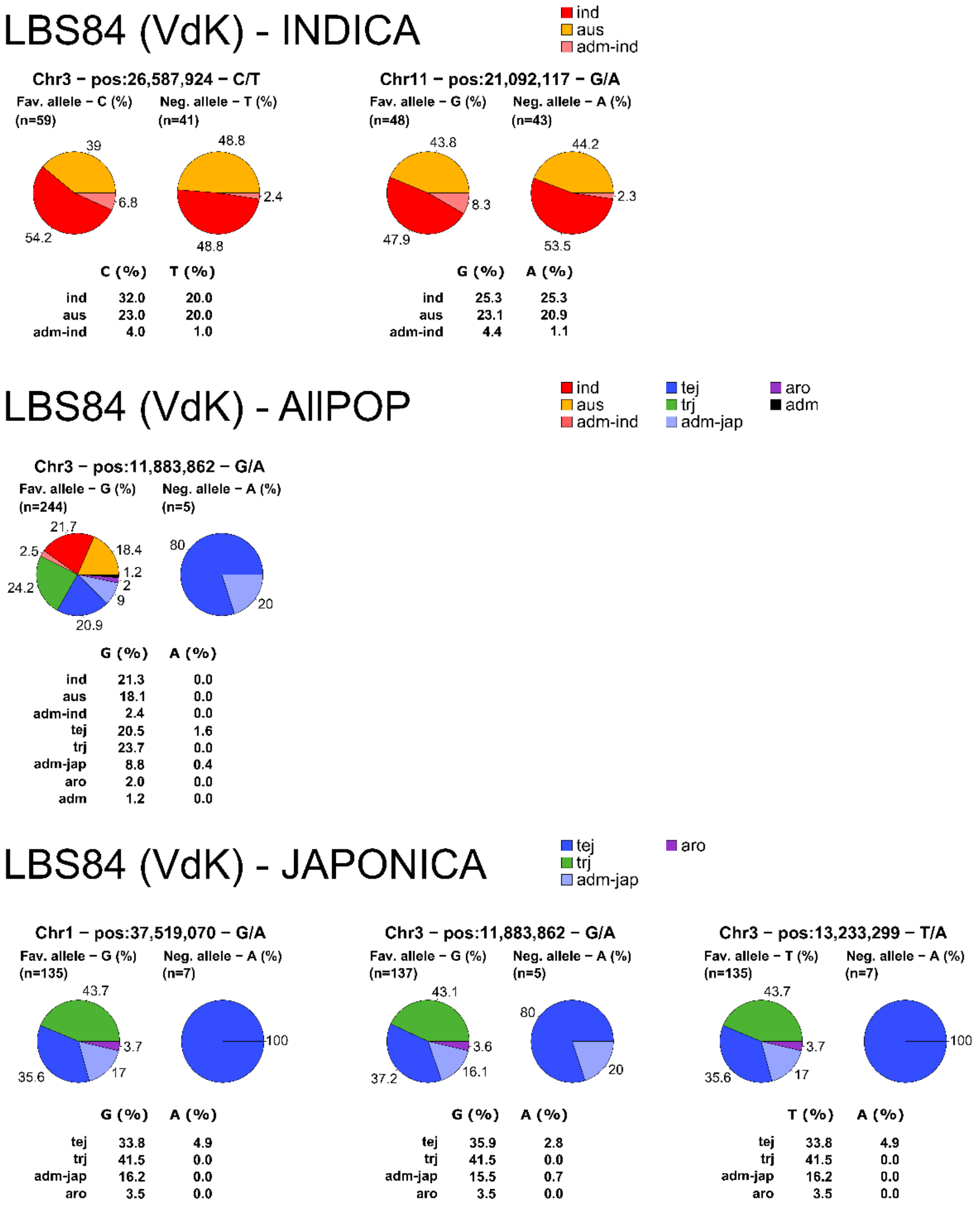

Supplementary Fig. S48. Frequencies (%) of the favorable (Fav.) and negative (Neg.) alleles for the most significant SNPs (msSNPs) of the QTLs (see Supplementary Table S4) identified using leaf bronzing scores 84 days after seeding (LBS84) in Vallee du Kou (VdK) in *INDICA* (top), *AllPOP* (middle) and *JAPONICA* (bottom). The number of accessions carrying the two alleles (n) is indicated in brackets. Pie charts and tables show the frequencies of the Fav. and Neg. alleles in the different subpopulations Subpopulation abbreviations: *ind=indica; aus=aus;* adm-*ind*=admixed-*indica; tej=temperate-japonica; trj=tropical-japonica;* adm-*jap*=admixed-*japonica; aro=aromatic*; adm=admixed.

LBS84 (HIA-All) - INDICA

Chr3 – pos:23,709,502 – T/G
Fav. allele – G (%) (n=9) | Neg. allele – T (%) (n=93)

Chr10 – pos:12,672,492 – C/T
Fav. allele – T (%) (n=11) | Neg. allele – C (%) (n=79)

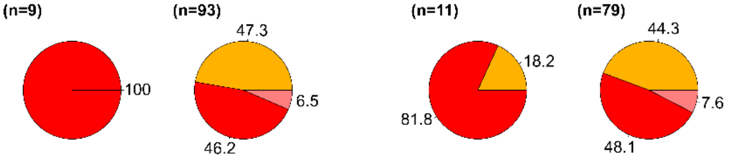

	G (%)	T (%)
ind	8.8	42.2
aus	0.0	43.1
adm-ind	0.0	5.9

	T (%)	C (%)
ind	10.0	42.2
aus	2.2	38.9
adm-ind	0.0	6.7

LBS84 (HIA-All) - AllPOP

Chr1 – pos:31,798,662 – A/G
Fav. allele – G (%) (n=12) | Neg. allele – A (%) (n=198)

Chr2 – pos:7,100,508 – T/A
Fav. allele – A (%) (n=12) | Neg. allele – T (%) (n=211)

Chr3 – pos:23,709,502 – T/G
Fav. allele – G (%) (n=9) | Neg. allele – T (%) (n=216)

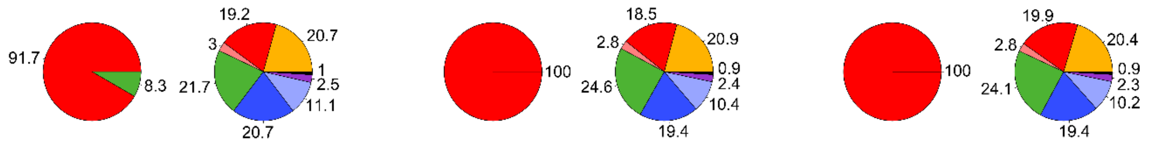

	G (%)	A (%)
ind	5.2	18.1
aus	0.0	19.5
adm-ind	0.0	2.9
tej	0.0	19.5
trj	0.5	20.5
adm-jap	0.0	10.5
aro	0.0	2.4
adm	0.0	1.0

	A (%)	T (%)
ind	5.4	17.5
aus	0.0	19.7
adm-ind	0.0	2.7
tej	0.0	18.4
trj	0.0	23.3
adm-jap	0.0	9.9
aro	0.0	2.2
adm	0.0	0.9

	G (%)	T (%)
ind	4.0	19.1
aus	0.0	19.6
adm-ind	0.0	2.7
tej	0.0	18.7
trj	0.0	23.1
adm-jap	0.0	9.8
aro	0.0	2.2
adm	0.0	0.9

Supplementary Fig. S49. Frequencies (%) of the favorable (Fav.) and negative (Neg.) alleles for the most significant SNPs (msSNPs) of the QTLs (see Supplementary Table S4) identified using leaf bronzing scores 84 days after seeding (LBS84) in all the HIA stress sites (Fe-All) in *INDICA* (top) and *AllPOP* (bottom). The number of accessions carrying the two alleles (n) is indicated in brackets. Pie charts and tables show the frequencies of the Fav. and Neg. alleles in the different subpopulations Subpopulation abbreviations: *ind=indica; aus=aus;* adm-*ind*=admixed-*indica; tej=temperate-japonica; trj=tropical-japonica;* adm-*jap*=admixed-*japonica; aro=aromatic*; adm=admixed.

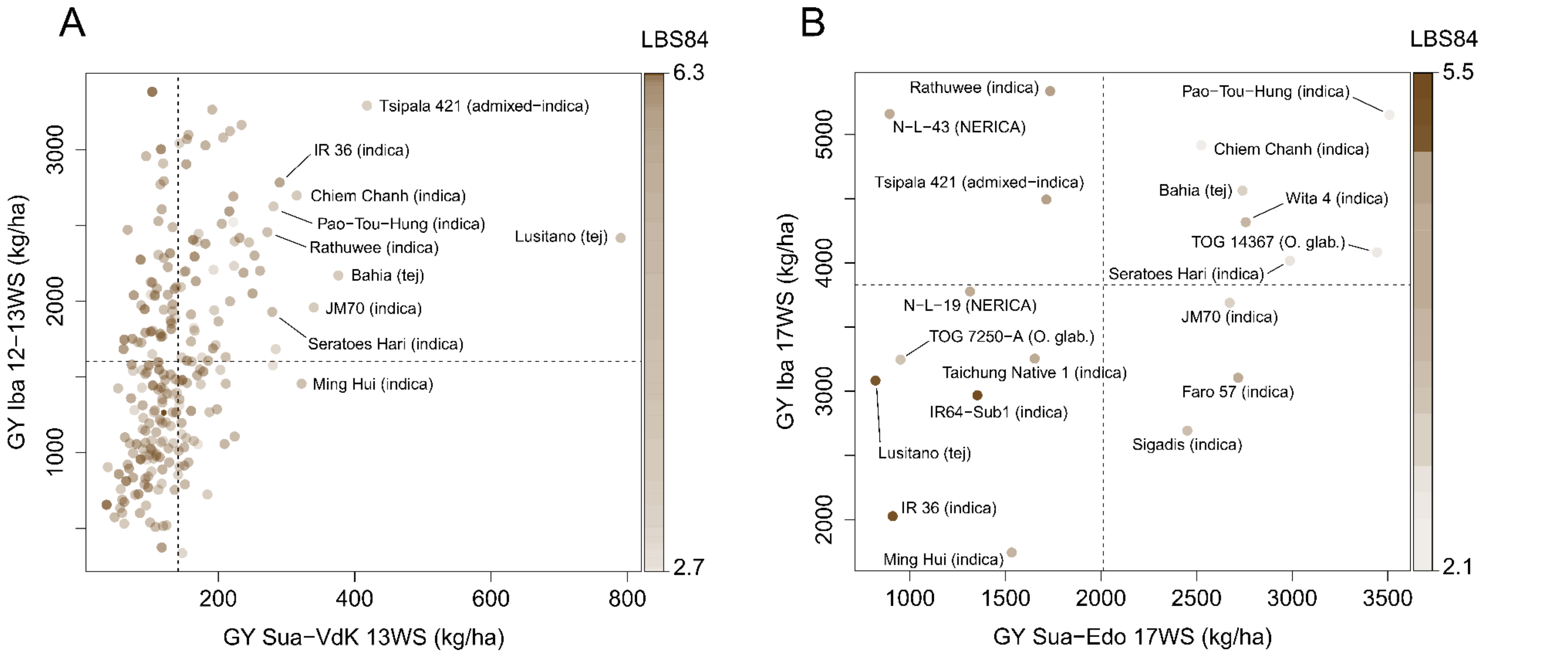

Supplementary Fig. S50. Re-evaluation of high yielding RDP1 accessions. Grain yield (GY) performance of RDP1 accessions in the field trials conducted in Ibadan (control), Suakoko and Valle du Kou (HIA stress sites) during the 2012-2013 WS (A-left). Grain yield (GY) performance of the highest-yielding RDP1 accessions (highlighted in A) and of AfricaRice elite accessions in the field trials conducted in Ibadan (control), Suakoko and Edozhigi (HIA stress sites) during the 2017 WS (B-right). Each accession is colored based on the mean LBS84 value determined in the Fe-toxic sites. LBS84 scales for A and B are displayed on the side of each plot and show the minimum and maximum value (used to rescale the accessions values between 0 and 1). Dotted lines indicate the mean GY value of the accessions on each axis.

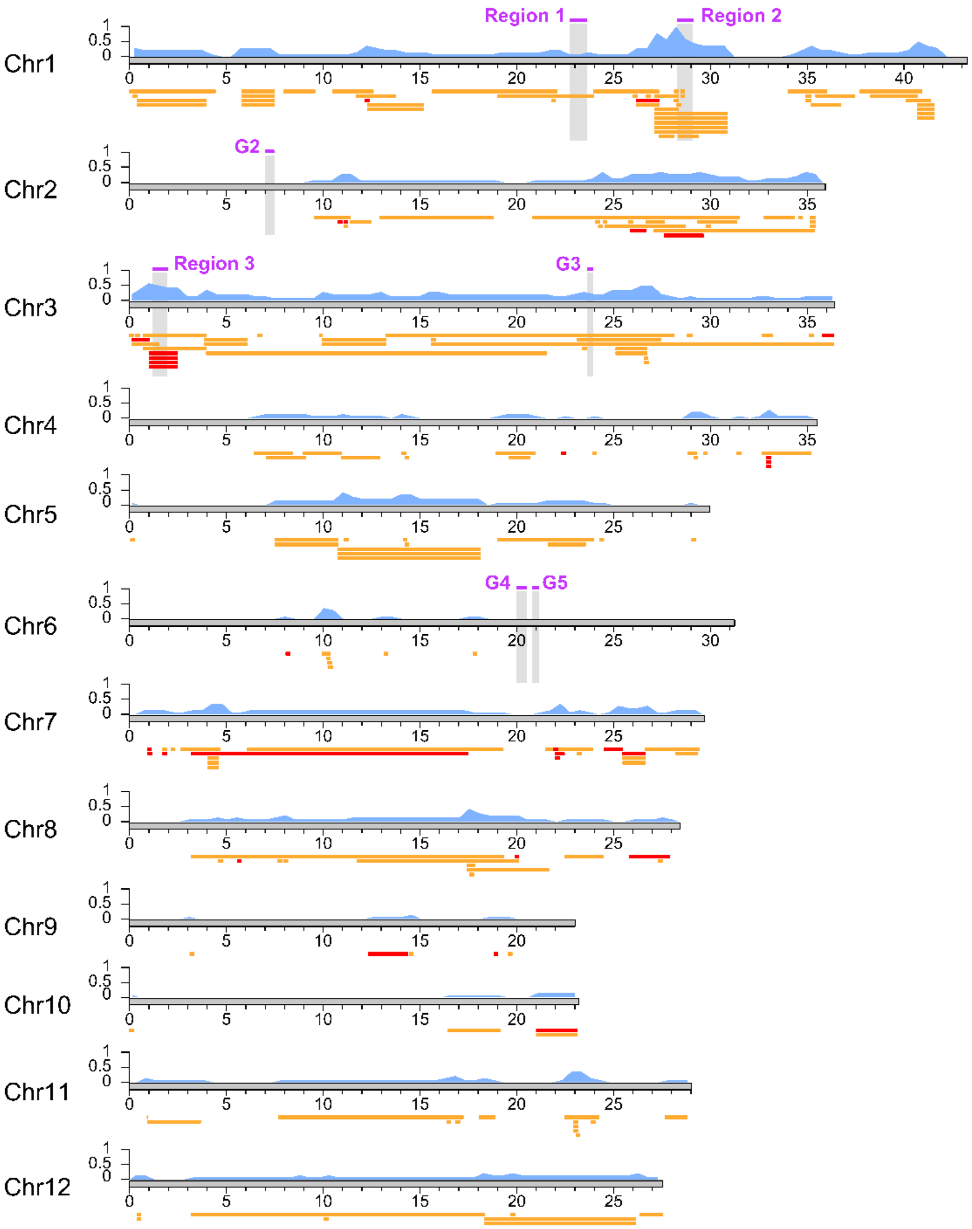

Supplementary Fig. S51. Inventory of QTLs for HIA stress tolerance in rice reported in the literature. QTLs determined in studies conducted in controlled environments (n=178) are represented in orange while QTLs determined in field experiments are represented in red (n=34). The genome-wide QTL density function (min=0; max=1) is represented by the blue curve on the top of each chromosome. The high-density QTL regions (bi-parental Regions 1-3 and GWAS regions G2-G5) determined in the current study are represented in purple above the density curves with their projection (in gray) over the QTLs from the literature. The minimum size of the represented QTLs was set to 200 kbp (even when the size was smaller) to make them visible in the plot. See material and methods for the full list of papers (13) used for the inventory and Supplementary Table S8 for further details on each QTL.
