# Supplementary material for: Multiple Small-Effect Alleles of Indica Origin Enhance High Iron-Associated Stress Tolerance in Rice Under Field Conditions in West Africa
Source: Front Plant Sci. 2021 Jan 15;11:604938. doi: 10.3389/fpls.2020.604938 (PMC7874229; doi:10.3389/fpls.2020.604938)
Supplement: Supplementary Table 2 — Number of SNPs per chromosome of the N-L-19 × IR64-Sub1 and N-L-43 × IR64-Sub1 SNP maps. [file Table_2.docx]

| Chromosome | **N-L-19 x IR64-Sub1** | **N-L-43 x IR64-Sub1** | **Shared SNPs** |
| --- | --- | --- | --- |
| 1 | 231 | 287 | 87 |
| 2 | 193 | 173 | 81 |
| 3 | 98 | 140 | 39 |
| 4 | 197 | 176 | 55 |
| 5 | 145 | 158 | 89 |
| 6 | 96 | 85 | 6 |
| 7 | 167 | 125 | 66 |
| 8 | 160 | 137 | 113 |
| 9 | 118 | 185 | 94 |
| 10 | 126 | 151 | 57 |
| 11 | 150 | 160 | 40 |
| 12 | 86 | 120 | 58 |
| Total | 1,767 | 1,897 | 785 |

**Supplementary Table S2.** Number of SNP (DArTseq™) markers per chromosome of the N-L-19 x IR64-Sub1 and N-L-43 x IR64-Sub1 SNP maps (for visual representation of marker distribution, see Supplemental Figures S1). The number of shared SNPs (SNPs with the same physical position) per chromosome is reported in the last column.
