# Supplementary material for: Multiple Small-Effect Alleles of Indica Origin Enhance High Iron-Associated Stress Tolerance in Rice Under Field Conditions in West Africa
Source: Front Plant Sci. 2021 Jan 15;11:604938. doi: 10.3389/fpls.2020.604938 (PMC7874229; doi:10.3389/fpls.2020.604938)
Supplement: Supplementary Table 3 — Summary information about 32 sequenced AfricaRice breeding lines. [file Table_3.docx]

**Supplementary Table S3.** Summary information about 32 sequenced AfricaRice breeding lines.

| Nr. | **Line name** | **Species (and subpop.)** | **Sequencing depth** | **Trait** |
| --- | --- | --- | --- | --- |
| 1 | BODIAN OUADEO | *O. glaberrima* | 6.0x | HIA stress |
| 2 | CG 14 | *O. glaberrima* | 4.9x | Other |
| 3 | Gervex 2674 | *O. glaberrima* | 5.3x | Flooding |
| 4 | IG 133 | *O. glaberrima* | 4.8x | Flooding |
| 5 | IG 48 | *O. glaberrima* | 6.5x | Flooding |
| 6 | TOG 14367 | *O. glaberrima* | 8.4x | HIA stress |
| 7 | TOG 16704 | *O. glaberrima* | 6.0x | Flooding |
| 8 | TOG 5485 | *O. glaberrima* | 3.8x | Flooding |
| 9 | TOG 5505-A | *O. glaberrima* | 5.9x | Flooding |
| 10 | TOG 5681 | *O. glaberrima* | 39.8x | Other |
| 11 | TOG 5980-A | *O. glaberrima* | 9.7x | Flooding |
| 12 | TOG 6218-B | *O. glaberrima* | 4.7x | HIA stress |
| 13 | TOG 6519-A | *O. glaberrima* | 5.2x | Drought |
| 14 | TOG 6520 | *O. glaberrima* | 3.7x | Drought |
| 15 | TOG 7148 | *O. glaberrima* | 4.4x | Flooding |
| 16 | TOG 7206 | *O. glaberrima* | 6.4x | HIA stress |
| 17 | TOG 7250-A | *O. glaberrima* | 3.0x | HIA stress |
| 18 | TOG 7252-A | *O. glaberrima* | 4.1x | Flooding |
| 19 | TOG 7400 | *O. glaberrima* | 5.4x | Drought |
| 20 | TOG 7442-B | *O. glaberrima* | 8.1x | Drought |
| 21 | TOG 8347 | *O. glaberrima* | 6.1x | Flooding |
| 22 | FARO 44 | *O. sativa* subpop. *indica* | 5.6x | Other |
| 23 | BOUAKE189 | *O. sativa* subpop. *indica* | 4.6x | Other |
| 24 | Taichung Native 1 | *O. sativa* subpop. *indica* | 4.2x | HIA stress |
| 25 | FARO 57 | *O. sativa* subpop. *indica* | 7.5x | Other |
| 26 | IR64-SUB1 | *O. sativa* subpop. *indica* | 5.2x | Other |
| 27 | NERICA-L-19 | *O. sativa* subpop. *indica* | 24.1x | HIA stress |
| 28 | Tchibanga | *O. sativa* subpop. *indica* | 4.5x | HIA stress |
| 29 | WITA 12 | *O. sativa* subpop. *indica* | 6.3x | Other |
| 30 | WITA 4 | *O. sativa* subpop. *indica* | 6.1x | HIA stress |
| 31 | TOS 6455 | *O. sativa* subpop. *indica* | 6.2x | Flooding |
| 32 | TOS 6454 | *O. sativa* subpop. *aus* | 4.1x | Flooding |
