# Supplementary material for: Multiple Small-Effect Alleles of Indica Origin Enhance High Iron-Associated Stress Tolerance in Rice Under Field Conditions in West Africa
Source: Front Plant Sci. 2021 Jan 15;11:604938. doi: 10.3389/fpls.2020.604938 (PMC7874229; doi:10.3389/fpls.2020.604938)
Supplement: Supplementary Table 4 — Significant GWA-QTLs identified for the traits scored in RDP1. [file Table_4.docx]

**Supplementary Table S4.** Significant GWA-QTLs identified for the traits scored in RDP1.

| **Trait** | **Varietal group** | **Site** | **Marker-based heritability (*h^2^*)^A^** | **Color code**  **and QTL name (as in Fig. 5)** | **Chr** | **msSNP (bp)^B^** | **-log_10_ (*P*-value)^C^** | **major/minor allele** | **MAF^D^** | **Mean allele effect (trait)^E^** | **Fav.**  **allele^F^** | **Del.**  **allele^F^** |
| --- | --- | --- | --- | --- | --- | --- | --- | --- | --- | --- | --- | --- |
| **GY** | INDICA | Ibadan | 0.47 | █ GY (Iba) | 2 | 7,176,097 | 5.74 | A/T | 0.47 | -20.00 | major | minor |
| (grams/plot) |  |  |  | █ GY (Iba) | 6 | 22,812,712 | 5.20 | C/A | 0.31 | +60.37 | minor | major |
|  | AllPOP | Ibadan | n/a | █ GY (Iba) | 5 | 2,239,377 | 5.03 | T/C | 0.05 | -92.62 | major | minor |
|  |  |  |  | █ GY (Iba) | 6 | 4,693,540 | 7.01 | G/A | 0.05 | -103.21 | major | minor |
|  |  |  |  | █ GY (Iba) | 6 | 5,207,971 | 5.49 | C/T | 0.10 | -75.43 | major | minor |
|  |  |  |  | █ GY (Iba) | 6 | 18,716,682 | 5.58 | T/C | 0.49 | +55.64 | minor | minor |
|  |  |  |  | █ GY (Iba) | 6 | 22,396,008 | 5.61 | C/T | 0.38 | -72.78 | major | minor |
|  |  |  |  | █ GY (Iba) | 7 | 17,039,592 | 7.84 | A/G | 0.07 | -89.40 | major | minor |
|  |  |  |  | █ GY (Iba) | 8 | 14,370,157 | 7.19 | T/C | 0.07 | -79.87 | major | minor |
|  | INDICA | Suakoko | 0.57 | █ GY (Sua) | 2 | 11,533,639 | 6.02 | T/C | 0.26 | +6.29 | minor | major |
|  |  |  |  | █ GY (Sua) | 3 | 20,084,929 | 5.27 | T/A | 0.27 | -1.19 | major | minor |
|  | AllPOP | Suakoko |  | █ GY (Sua) | 2 | 11,559,890 | 6.20 | T/C | 0.06 | +5.98 | minor | major |
|  |  |  | n/a | █ GY (Sua) | 3 | 20,084,582 | 5.47 | C/T | 0.13 | -1.56 | major | minor |
|  |  |  |  | █ GY (Sua) | 3 | 32,991,022 | 5.71 | G/A | 0.17 | -0.89 | major | minor |
|  | JAPONICA | Suakoko | 0.12 | █ GY (Sua) | 1 | 39,473,943 | 5.06 | C/T | 0.05 | -4.26 | major | minor |
|  | INDICA | Vallee du Kou | 0.96 | █ GY (VdK) | 2 | 34,458,920 | 6.08 | A/G | 0.33 | -4.78 | major | minor |
|  |  |  |  | █ GY (VdK) | 3 | 23,791,037 | 5.07 | A/G | 0.18 | -5.05 | major | minor |
|  |  |  |  | █ GY (VdK) | 4 | 31,822,621 | 5.10 | C/T | 0.48 | +7.53 | minor | major |
|  |  |  |  | █ GY (VdK) | 6 | 25,164,784 | 5.41 | T/C | 0.31 | +9.06 | minor | major |
|  |  |  |  | █ GY (VdK) | 8 | 2,206,066 | 5.19 | T/A | 0.32 | -8.63 | major | minor |
|  |  |  |  | █ GY (VdK) | 8 | 28,413,679 | 5.23 | G/A | 0.07 | -10.56 | major | minor |
|  | AllPOP | Vallee du Kou |  | █ GY (VdK) | 3 | 1,278,045 | 5.18 | G/A | 0.39 | -6.52 | major | minor |
|  |  |  | n/a | █ GY (VdK) | 5 | 24,791,653 | 5.28 | A/G | 0.45 | 4.36 | minor | major |
|  |  |  |  | █ GY (VdK) | 8 | 1,725,770 | 5.07 | C/A | 0.15 | 10.16 | minor | major |
| **GY-loss** | INDICA | Suakoko | 0.56 | █ GY-loss (Sua) | 1 | 22,486,213 | 5.41 | G/T | 0.07 | -5.41 | minor | major |
| (%) |  |  |  | █ GY-loss (Sua) | 2 | 7,329,216 | 5.36 | C/T | 0.43 | 3.48 | major | minor |
|  |  |  |  | █ GY-loss (Sua) | 3 | 20,172,883 | 5.44 | G/A | 0.06 | -5.20 | minor | major |
|  |  |  |  | █ GY-loss (Sua) | 6 | 20,426,945 | 5.16 | T/A | 0.11 | -4.39 | minor | major |
|  |  |  |  | █ GY-loss (Sua) | 6 | 21,001,653 | 5.42 | C/T | 0.09 | -3.32 | minor | major |
|  |  |  |  | █ GY-loss (Sua) | 7 | 20,629,262 | 6.05 | G/A | 0.06 | -5.94 | minor | major |
|  |  |  |  | █ GY-loss (Sua) | 9 | 17,708,388 | 7.44 | A/G | 0.08 | -4.87 | minor | major |
|  |  |  |  | █ GY-loss (Sua) | 10 | 21,844,289 | 5.37 | C/T | 0.06 | -5.54 | minor | major |
|  | AllPOP | Suakoko | n/a | █ GY-loss (Sua) | 1 | 9,607,049 | 6.18 | A/C | 0.05 | -5.47 | minor | major |
|  | INDICA | Vallee du Kou | 0.77 | █ GY-loss (VdK) | 1 | 23,302,758 | 5.21 | C/A | 0.09 | -8.81 | minor | major |
|  | AllPOP | Vallee du Kou | n/a | █ GY-loss (VdK) | 2 | 5,349,105 | 5.17 | G/A | 0.16 | +0.41 | major | minor |
|  |  |  |  | █ GY-loss (VdK) | 6 | 20,102,783 | 5.57 | C/T | 0.09 | -7.09 | minor | major |
|  | JAPONICA | Vallee du Kou | 0.13 | █ GY-loss (VdK) | 6 | 20,102,783 | 5.38 | C/T | 0.13 | -7.91 | minor | major |
|  | INDICA | All HIA stress sites | 0.34 | █ GY-loss (HIA-All) | 1 | 3,683,473 | 5.02 | G/T | 0.36 | +2.93 | major | minor |
|  |  |  |  | █ GY-loss (HIA-All) | 6 | 20,953,952 | 5.17 | G/T | 0.09 | -4.63 | minor | major |
|  |  |  |  | █ GY-loss (HIA-All) | 6 | 21,434,466 | 5.26 | C/T | 0.11 | +3.28 | major | minor |
|  | AllPOP | All HIA stress sites | n/a | █ GY-loss (HIA-All) | 1 | 23,358,388 | 5.24 | T/C | 0.05 | -4.53 | minor | major |
|  |  |  |  | █ GY-loss (HIA-All) | 6 | 20,953,952 | 5.16 | G/T | 0.05 | -4.02 | minor | major |
|  |  |  |  | █ GY-loss (HIA-All) | 12 | 17,954,641 | 5.68 | G/A | 0.07 | -5.72 | minor | major |
|  | JAPONICA | All HIA stress sites | 0.22 | █ GY-loss (HIA-All) | 7 | 252,852 | 6.14 | C/T | 0.05 | -10.10 | minor | major |
| **LBS84** | INDICA | Edozhigi | 0.06 | █ LBS84 (Edo) | 1 | 15,336,902 | 5.09 | C/T | 0.30 | 0.19 | major | minor |
| (bronzing |  |  |  | █ LBS84 (Edo) | 1 | 15,810,576 | 5.17 | C/T | 0.29 | 0.20 | major | minor |
| score) |  |  |  | █ LBS84 (Edo) | 1 | 16,313,039 | 5.67 | G/A | 0.24 | 0.23 | major | minor |
|  |  |  |  | █ LBS84 (Edo) | 1 | 16,691,047 | 5.57 | C/A | 0.17 | 0.26 | major | minor |
|  |  |  |  | █ LBS84 (Edo) | 1 | 17,220,686 | 5.28 | G/T | 0.29 | 0.20 | major | minor |
|  |  |  |  | █ LBS84 (Edo) | 11 | 2,722,424 | 5.41 | G/A | 0.28 | 0.24 | major | minor |
|  | AllPOP | Edozhigi | n/a | █ LBS84 (Edo) | 1 | 15,336,902 | 5.23 | C/T | 0.14 | 0.21 | major | minor |
|  |  |  |  | █ LBS84 (Edo) | 1 | 15,810,576 | 5.19 | C/T | 0.13 | 0.22 | major | minor |
|  |  |  |  | █ LBS84 (Edo) | 1 | 16,303,188 | 5.47 | G/A | 0.13 | 0.21 | major | minor |
|  |  |  |  | █ LBS84 (Edo) | 1 | 16,657,330 | 5.74 | G/C | 0.15 | 0.22 | major | minor |
|  |  |  |  | █ LBS84 (Edo) | 1 | 17,220,686 | 5.51 | G/T | 0.13 | 0.22 | major | minor |
|  |  |  |  | █ LBS84 (Edo) | 11 | 2,722,424 | 5.08 | G/A | 0.11 | 0.24 | major | minor |
|  | AllPOP | Suakoko | n/a | █ LBS84 (Sua) | 11 | 22,032,907 | 5.00 | C/T | 0.12 | -0.50 | minor | major |
|  | INDICA | Vallee du Kou | 0.92 | █ LBS84 (VdK) | 3 | 26,587,924 | 5.18 | C/T | 0.41 | +0.51 | major | minor |
|  |  |  |  | █ LBS84 (VdK) | 11 | 21,092,117 | 5.83 | G/A | 0.47 | +0.52 | major | minor |
|  | AllPOP | Vallee du Kou | n/a | █ LBS84 (VdK) | 3 | 11,883,862 | 5.23 | G/A | 0.05 | +0.84 | major | minor |
|  | JAPONICA | Vallee du Kou | 0.02 | █ LBS84 (VdK) | 1 | 37,519,070 | 6.19 | G/A | 0.05 | +0.82 | major | minor |
|  |  |  |  | █ LBS84 (VdK) | 3 | 11,883,862 | 6.22 | G/A | 0.05 | +0.99 | major | minor |
|  |  |  |  | █ LBS84 (VdK) | 3 | 13,233,299 | 6.18 | T/A | 0.05 | +0.82 | major | minor |
|  | INDICA | All HIA stress sites | 0.89 | █ LBS84 (HIA-All) | 3 | 23,709,502 | 5.11 | T/G | 0.09 | -0.52 | minor | major |
|  |  |  |  | █ LBS84 (HIA-All) | 10 | 12,672,492 | 5.28 | C/T | 0.12 | -0.45 | minor | major |
|  | AllPOP | All HIA stress sites | n/a | █ LBS84 (HIA-All) | 1 | 31,798,662 | 5.01 | A/G | 0.07 | -0.30 | minor | major |
|  |  |  |  | █ LBS84 (HIA-All) | 2 | 7,100,508 | 5.21 | T/A | 0.05 | -0.35 | minor | major |
|  |  |  |  | █ LBS84 (HIA-All) | 3 | 23,709,502 | 5.82 | T/G | 0.05 | -0.42 | minor | major |

^A^ Phenotypic variance explained by the IBS matrix (or pseudo-heritability, *h^2^*)

^B^ Chromosomal position (bp) of the most significant SNP (msSNP) in the QTL region.

^C^ Significance (-log_10_*P*) of the msSNP in the QTL region.

^D^ Minor allele frequency (MAF) at the msSNP.

^E^ Allele effect on trait of the msSNP calculated as [mean trait value in accessions carrying the major allele] - [mean trait value in accessions carrying the minor allele].

^F^ The favorable (fav.) allele is associated to higher GY and lower GY-loss and LBS84. The deleterious (del.) allele has the opposite effect of the favorable one.
