# Supplementary material for: Multiple Small-Effect Alleles of Indica Origin Enhance High Iron-Associated Stress Tolerance in Rice Under Field Conditions in West Africa
Source: Front Plant Sci. 2021 Jan 15;11:604938. doi: 10.3389/fpls.2020.604938 (PMC7874229; doi:10.3389/fpls.2020.604938)
Supplement: Supplementary Analysis 1 — In-depth examination of DTH3 variation among the AfricaRice and RDP1 INDICA accessions. [file Data_Sheet_4.docx]

**Supplementary Analysis S1:**

The majority of bi-parental QTLs detected in Region 3 were associated with flowering time under both control and HIA stress conditions (Fig. 2). The most significant marker of these flowering time QTLs was the same in both bi-parental Pop1 and Pop 2 (SNP with pos: 1,248,074), was highly significant (LOD =102.2 and 87.7 in the control and HIA stress sites in Pop1; LOD =19.4 and 23.1 in the control and HIA stress sites in Pop2) and was associated with a difference of 9.7-8.7 and 5.0-6.6 days to flowering, respectively, when the populations were grown in control or HIA stress environments, respectively (Table 3). This msSNP is localized ~22 kb downstream from the *DAYS TO HEADING 3* (*DTH3/OsMADS50*) gene in Region 3 (Os03g01122600; pos: 1,270,320-1,300,273 bp). *DTH3* is involved in flowering induction in both Asian and African rice (Lee et al., 2004; Bian et al., 2011). Given the importance of flowering time on yield performance, especially under stress (Fig. 1), it is intriguing that the msSNP (pos: 1,278,045) for the GY QTL in Valle du Kou is located within a large intron of the *DTH3* gene. To determine whether variation within the flowering time gene itself is more predictive of grain yield under stress in Valle du Kou than was the extended haplotype, we undertook a more in-depth examination of *DTH3* variation among the *INDICA* RDP1 accessions.

For this purpose, we used a high resolution SNP dataset (4.8M SNPs) for the RDP1 referred to as the RICE-RP dataset (Wang et al., 2018). The integration of the RICE-RP with the sequencing data generated for the AfricaRice breeding lines made it possible to identify four main *DTH3* gene haplotypes among the AfricaRice and *INDICA* RDP1 accessions (Figure A, below). N-L-19 carried a gene haplotype (GH1) that was common among RDP1 *indica* accessions (82%) and also found in 86% of *aus* accessions. IR64-Sub1 carried a gene haplotype (GH2) that was found in 11% of RDP1 *indica* and 8% of *aus* accessions (Supplementary Fig. S50). As previously described, we then used GH1 (NL-L-19) and GH2 (IR64-Sub1) to define genotypic groups within RDP1 *INDICA* accessions and compared GY for the two groups observed in Valle du Kou. As with the extended haplotypes (EH1 and EH2), we again found no significant difference (t-test, *p*>0.05). We therefore conclude that while *DTH3* haplotypes are strongly associated with FLW across locations in the bi-parental populations, and with GY in Suakoko, they are not predictive of GY in RDP1 *INDICA* accessions evaluated in Valle du Kou.

The *DTH3* gene-based haplotype analysis in Region 3 provided further insight into the ancestry of the NERICA parents. We observed a single, *O. glaberrima-*specific extended haplotype (EH4; Figure A, below) among the 21 *O. glaberrima* lines of the AfricaRice panel, but these same lines contained two clearly differentiated gene haplotypes (GH3 and GH4) across *DTH3* (Figure A, below). The haplotypes seen in N-L-19 (EH1 and GH1) and IR64-Sub1 (EH2 and GH2) were clearly different from the *O. glaberrima* haplotypes (EH4, GH3 and GH4) in Region 3. This observation further supports the conclusion that neither of the NERICA parents in the bi-parental populations (TOG5681 and TOG5674) carried *O. glaberrima* alleles across the three QTL regions examined here. Indeed, as previously mentioned, the N-L-19 parent was shown to have inherited this region of its genome from an *indica-*like ancestor.


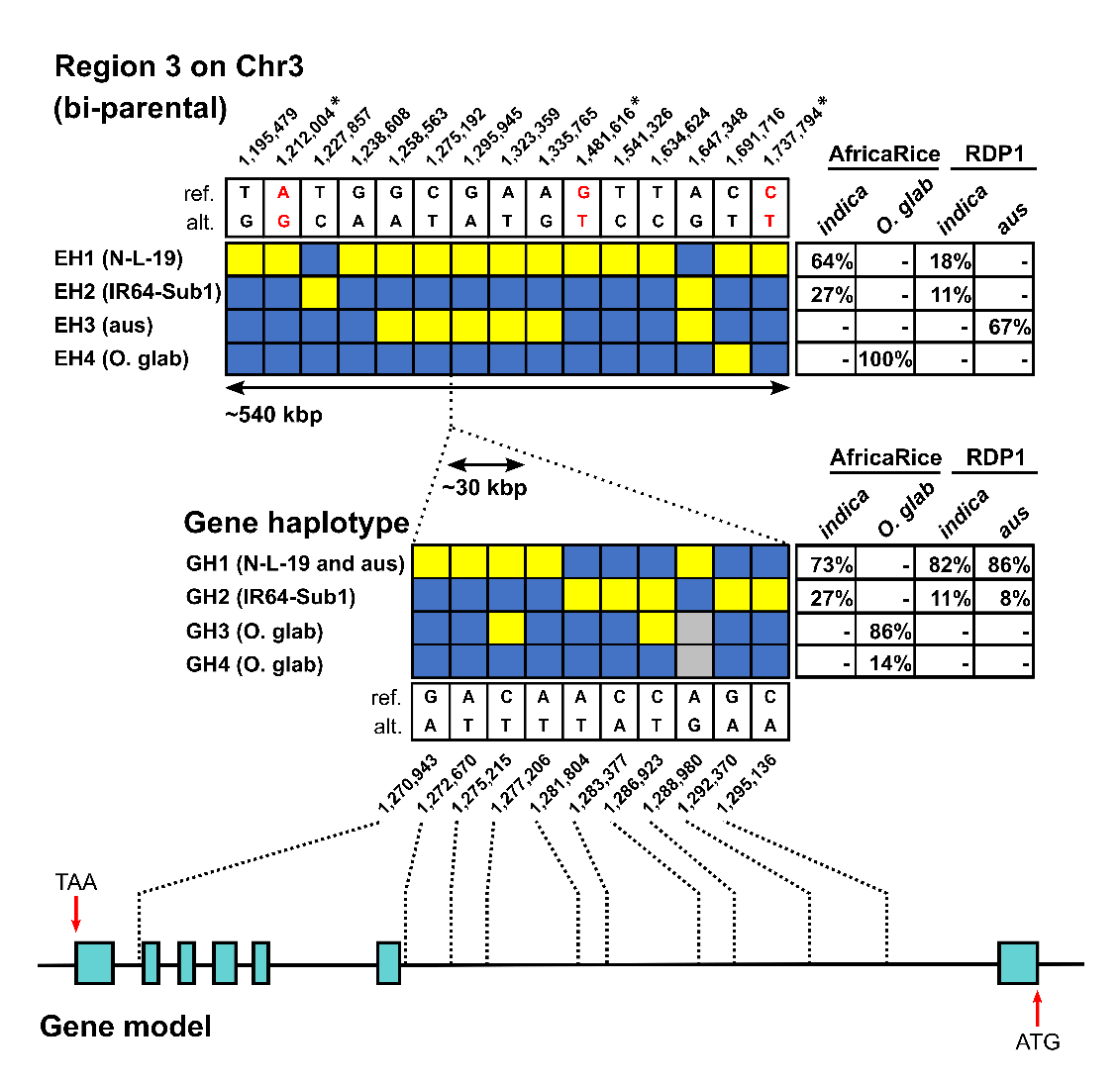


**Supplementary Analysis Figure A.** Main haplotypes found in the high-density bi-parental QTL Region 3 (already shown and described in Fig. 4) and zoom-in the gene haplotype of *DTH3*/*OsMADS50* (Os03g0122600); The gene haplotype is constructed based on a set of 10 SNPs; blue rectangles=reference (cv Nipponbare) allele; yellow=alternate allele; grey=missing data. SNP positions (bp) shown next to reference (ref.) and alternative (alt.) alleles; SNPs predicted to cause non-synonymous substitutions highlighted in red and indicated with an asterisk (*). In Regions 3, the main ‘extended’ haplotypes found are named EH1= N-L-19 (female parent), EH2=IR64-Sub1 (male parent), EH3=*aus*-like, EH4=*O. glaberrima*-like. For the gene, the main ‘gene’ haplotypes found are named GH1= N-L-19 (female parent) and *aus*-like, GH2=IR64-Sub1 (male parent), GH3=*O. glaberrima*-like. Frequencies of each haplotype in different groups of accessions are indicated in tables where *indica* - AfricaRice group= 10 *indica* and 1 NERICA*; indica* - RDP1 group= 65 *indica* and 6 *admixed-indica*; *O. glab* – AfricaRice group= 21 *O. glaberrima*; *aus* - RDP1 group= 49 *aus* lines (see Supplementary Tables S1 and S3). The complete absence of a haplotype in a group is indicated by (-). The *DTH3* gene model (exons in blue) is reported at the bottom of the figure with the projection of the 10 SNPs on it. ATG: start codon; TAA: stop codon.

**References:**

Bian, X.F., Liu, X., Zhao, Z.G., Jiang, L., Gao, H., Zhang, Y.H., et al. (2011). Heading date gene, dth3 controlled late flowering in *O. Glaberrima Steud.* by down-regulating Ehd1. *Plant Cell Rep* 30(12)**,** 2243-2254. doi: 10.1007/s00299-011-1129-4.

Lee, S., Kim, J., Han, J.-J., Han, M.-J., and An, G. (2004). Functional analyses of the flowering time gene OsMADS50, the putative suppressor of overexpresison of CO 1/Agamous-Like 20 (SOC1/AGL20) ortholog in rice. *The Plant Journal* 38(5)**,** 754-764. doi: 10.1111/j.1365-313X.2004.02082.x.

Wang, D.R., Agosto-Perez, F.J., Chebotarov, D., Shi, Y., Marchini, J., Fitzgerald, M., et al. (2018). An imputation platform to enhance integration of rice genetic resources. *Nat Commun* 9(1)**,** 3519. doi: 10.1038/s41467-018-05538-1.
